# The use of 4,4,4-trifluorothreonine to stabilize extended peptide structures and mimic β-strands

**DOI:** 10.3762/bjoc.13.276

**Published:** 2017-12-21

**Authors:** Yaochun Xu, Isabelle Correia, Tap Ha-Duong, Nadjib Kihal, Jean-Louis Soulier, Julia Kaffy, Benoît Crousse, Olivier Lequin, Sandrine Ongeri

**Affiliations:** 1 Molécules Fluorées et Chimie Médicinale, BioCIS, Univ. Paris-Sud, CNRS, Université Paris Saclay, 5 rue Jean-Baptiste Clément, 92296 Châtenay-Malabry Cedex, France; 2 Sorbonne Universités, UPMC Univ Paris 06, Ecole Normale Supérieure, PSL Research University, CNRS, Laboratoire des Biomolécules, 4 place Jussieu, 75252 Paris Cedex 05, France

**Keywords:** aggregation, beta-sheet, fluorine, peptide, unnatural amino acid

## Abstract

Pentapeptides having the sequence R-HN-Ala-Val-**X**-Val-Leu-OMe, where the central residue **X** is L-serine, L-threonine, (2*S*,3*R*)-L-CF_3_-threonine and (2*S*,3*S*)-L-CF_3_-threonine were prepared. The capacity of (2*S*,3*S*)- and (2*S*,3*R*)-CF_3_-threonine analogues to stabilize an extended structure when introduced in the central position of pentapeptides is demonstrated by NMR conformational studies and molecular dynamics simulations. CF_3_-threonine containing pentapeptides are more prone to mimic β-strands than their natural Ser and Thr pentapeptide analogues. The proof of concept that these fluorinated β-strand mimics are able to disrupt protein–protein interactions involving β-sheet structures is provided. The CF_3_-threonine containing pentapeptides interact with the amyloid peptide Aβ_1-42_ in order to reduce the protein–protein interactions mediating its aggregation process.

## Introduction

It is estimated that 20% of administered drugs contain fluorine atoms or fluoroalkyl groups, representing 150 fluorinated molecules, and this trend is expected to increase to about 30% in the early future as a new generation of fluorinated compounds is currently in Phase II−III clinical trials [1]. In parallel, pharmaceutical peptides are attracting increasing interest as around 100 peptides are on the pharmaceutical market [2]. Peptide fluorination has appeared as a general and effective strategy to enhance the stability against enzymatic, chemical and thermal denaturation while generally retaining the original structure and biological activity [3–4]. Fluorinated amino acids can also be used as powerful ^19^F NMR probes for the study of protein–ligand interactions and enzymatic activities [5–8]. However, the development of fluorinated peptides as drug candidates seems to be largely under-exploited. Investigation on the influence of a fluorinated substituent incorporated in the side-chain of amino acids on peptide conformations has recently raised attention [9]. While the effect of fluorinated analogs of hydrophobic aliphatic and aromatic amino acids has been prominently studied, the influence of fluorinated polar amino acids has been rarely explored. To our knowledge, only one example of conformational studies of a peptide containing a (2*S*,3*S*)-CF_3_-threonine has been conducted by Kitamoto et al. [7,10]. These authors reported a significant conformational difference between an enkephalin-related hexapeptide derivative and its fluorinated analogue containing a (2*S*,3*S*)-CF_3_-threonine at its C-terminus. NMR studies demonstrated that the natural hexapeptide adopted a folded conformation while for the trifluoromethylated analogue an extended backbone conformation predominated.

**Figure 1:**
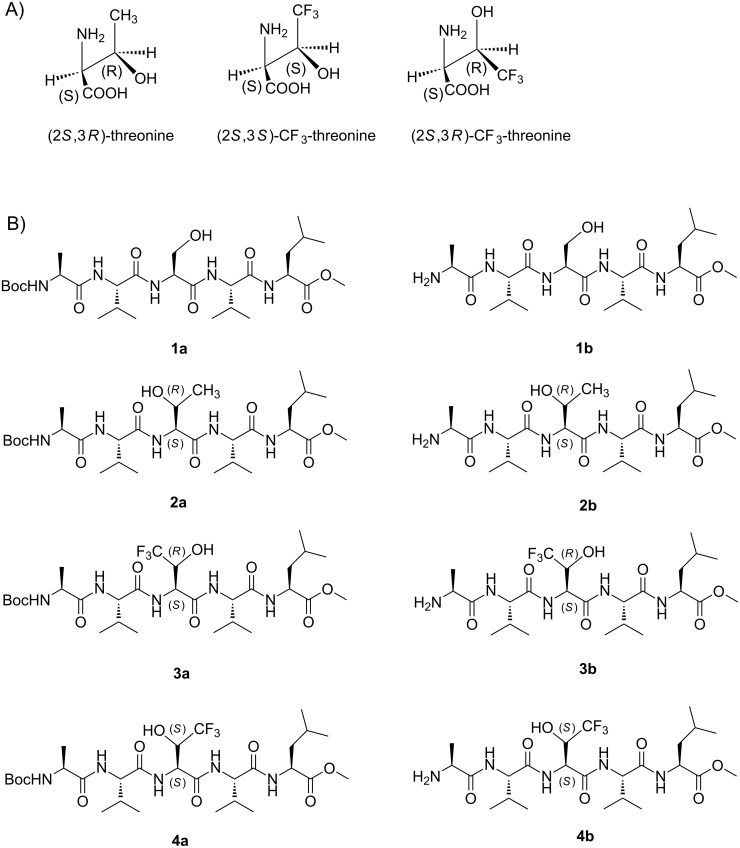
A) Natural threonine and its trifluoromethyl analogues sawhorse projections. B) Structure of Boc-protected pentapeptides **1a**–**4a** and free amine pentapeptides **1b**–**4b**.

In the present study, our objective was to evaluate the capacity of both (2*S*,3*S*)- and (2*S*,3*R*)-CF_3_-threonine analogues (the (2*S*,3*S*)- analogue being the exact analogue of the natural threonine residue, see Figure 1A) to stabilize an extended structure when introduced in the central position of pentapeptides, with the intent of designing inducer or stabilizer of β-strand mimics. Indeed, β-strand mimics have a particular interest as ligand of β-sheet structures and as potential inhibitors of protein–protein interactions involving β-sheet structures [11–13]. For example, β-strand mimics have been successfully introduced in inhibitors of amyloid proteins aggregation characterized by ordered β-sheet structure assemblies [14–15]. In this context, we synthesized and analyzed, by NMR and molecular modeling, the conformational preferences of eight pentapeptides, containing a L-serine, a L-threonine, a (2*S*,3*R*)-L-*allo*-CF_3_-threonine or a (2*S*,3*S*)-L-CF_3_-threonine in the third position. Both *N*-Boc protected (compounds **1a**–**4a**) and *N*-deprotected pentapeptides (**1b**–**4b**) were studied.

## Results and Discussion

**Scheme 1:**
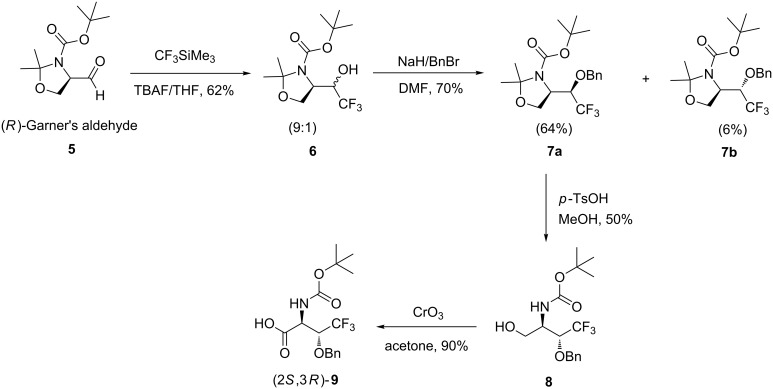
Synthesis of (2*S*,3*R*)-Boc-CF_3_-Thr(Bzl) **9**.

**Synthesis.** First, we synthesized the two (2*S*,3*R*)- and (2*S*,3*S*)-CF_3_-Thr analogues. An enantioselective synthesis of (2*S*,3*R*)-Boc-CF_3_-Thr was proposed in 2003 [16] from propargylic alcohol in ten steps, based on the trifluoromethylation key step of 1-(((*E*)-3-bromoallyloxy)methyl)benzene to obtain (*E*)-1-benzyloxy-4,4,4-trifluoro-2-butene. The sequence then involved Sharpless asymmetric dihydroxylation, nucleophilic opening of cyclic sulfate with NaN_3_, palladium-catalyzed selective hydrogenation, and oxidation. Zeng et al*.* described the synthesis of the enantiomer (2*R*,3*S*)-Boc-CF_3_-Thr(Bzl) in four steps from the (*S*)-Garner’s aldehyde [17–18]. The enantiomer (2*S*,3*R*)-Boc-CF_3_-Thr(Bzl) was not described by Zeng et al. However, we decided to follow this more straightforward methodology and we have adapted Zeng’s synthesis starting from the (*R*)-Garner’s aldehyde. (2*S*,3*R*)-Boc-CF_3_-Thr(Bzl) was obtained with satisfactory yields (Scheme 1). In this synthetic pathway, the key intermediate **6** was obtained, as a mixture of two diastereoisomers (9:1, evaluated by ^19^F NMR) via a nucleophilic trifluoromethylation reaction of Ruppert’s reagent on the (*R*)-Garner’s aldehyde **5** in THF and in the presence of a catalytic amount of TBAF. Benzylation of the alcohol of **6** was then performed to obtain the desired intermediate as two diastereoisomers **7a** and **7b** that were easily separated at this stage by column chromatography. The major diastereomer **7a** was used in the following steps. Hydrolysis of the oxazolidine, followed by Jones oxidation of the alcohol **8**, allowed us to recover the desired acid **9** in good yield (90%). The optical rotation of a solution of the product **9** (2*S*,3*R*), dissolved in MeOH was measured at 25 °C. The value obtained was equal to −13° and opposite to the value (+13°) described by Zeng et al. [17] for the enantiomer (2*R*,3*S*).

**Scheme 2:**
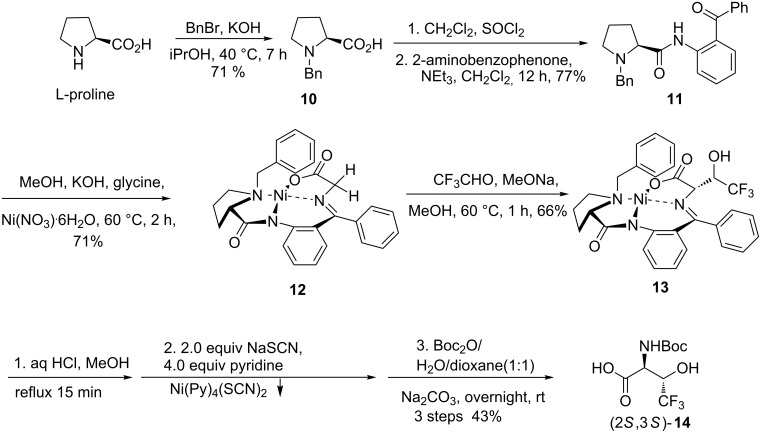
Synthesis of (2*S*,3*S*)-Boc-CF_3_-Thr **14**.

The synthesis of (2*S*,3*S*)-CF_3_-threonine has been described in several publications [7,10,19–22]. Among these approaches, we followed a general procedure to access to (2*S*,3*S*)-CF_3_-threonine through an aldol reaction of CF_3_CHO with the Ni(II) complex of the chiral Schiff base of glycine which was introduced by Belokon et al. [23–24]. The chiral auxiliary (*S*)-*N*-(2-benzoylphenyl)-1-benzylpyrrolidine-2-carboxamide (**11**) was obtained in good yield starting from the *N*-benzylation of L-proline in the presence of KOH, then activation of the carboxylic acid functionality of **10** using SOCl_2_ at low temperature, followed by condensation with 2-aminobenzophenone (Scheme 2). Complexation of **11** with nickel nitrate and glycine under basic conditions gave the nickel Schiff base complex **12** in 71% yield as red crystals. The nucleophilic glycine equivalent **12** went through the aldol reaction with trifluoroacetaldehyde to give complex **13** in moderate yield (66%). Further hydrolysis of complex **13** led to the recovery of the chiral auxiliary **11** and release of free (2*S*,3*S*)-CF_3_-threonine whose diastereoselectivity was determined to be about 96% by ^19^F NMR. Although in most of the reported cases, the free amino acid was released into the aqueous phase, and then purified by ion-exchange chromatography, we purified the free (2*S*,3*S*)-CF_3_-threonine by another way. We first managed to remove the Ni(II) by addition of 2.0 equivalents of NaSCN and 4.0 equivalents of pyridine to form the complex Ni(Py)_4_(SCN)_2_, which precipitated from the aqueous phase. After filtration, we protected the free amino acid using Boc_2_O under basic conditions. The Boc-(2*S*,3*S*)-CF_3_-threonine **14** was then purified by silica column chromatography and was obtained in 43% yield after three steps from **13** (Scheme 2).

**Scheme 3:**
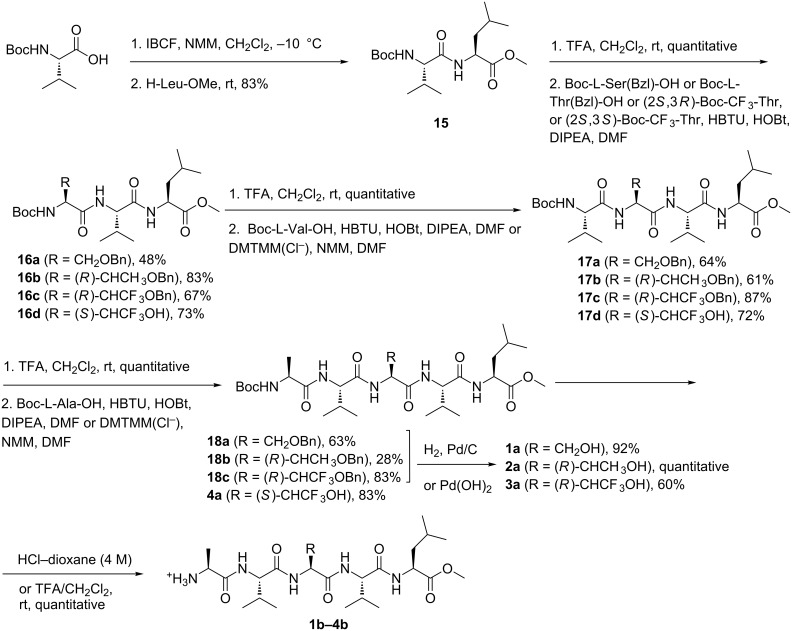
Synthesis of pentapeptides **1a**–**4a** and **1b**–**4b**.

Classical peptide synthesis in solution was used to prepare pentapeptides **1a**–**4a** (Scheme 3). Boc-L-Val-OH was activated by isobutylchloroformate (IBCF) and then coupled with L-Leu-OMe to afford dipeptide **15**. Acidic hydrolysis of **15** using TFA, and coupling with the third amino acid (Boc-L-Ser(Bzl)-OH, Boc-L-Thr(Bzl)-OH, (2*S*,3*R*)-Boc-CF_3_-Thr, (2*S*,3*S*)-Boc-CF_3_-Thr), using HBTU/HOBt in the presence of DIPEA in DMF, afforded tripeptides **16a**–**d** in good yields (48–83%). The tripeptides **16a**–**d** were deprotected using TFA, and the salt of the free amine was coupled to Boc-L-Val-OH using HBTU/HOBt/DIPEA or DMTMM(Cl^−^)/NMM to afford tetrapeptides **17a**–**c** and **17d** respectively, in satisfactory yields (61–87%). The pentapeptides **18a**–**c** and **4a** were obtained by deprotecting tetrapeptides **17a**–**d** with TFA and then performing the coupling reaction with Boc-L-Ala-OH in the presence of HBTU/HOBt/DIPEA or DMTMM(Cl^−^)/NMM. Catalytic hydrogenation, using 10% Pd/C or Pd(OH)_2_, under H_2_ atmosphere, gave pentapeptides **1a**–**3a** in moderate to quantitative yield. After acidic removal of the Boc group, the pentapeptide salts **1b**–**4b** were obtained in quantitative yield.

**Conformational studies.** The conformational properties of the eight pentapeptides (**1a**–**4a** and **1b**–**4b**) were examined by NMR analyses in a protic solvent, which is more challenging than in aprotic organic solvents for maintaining intramolecular hydrogen bond network. Methanol was used because of the limited solubility of these compounds in aqueous solutions. The ^1^H and ^13^C chemical shifts of these pentapeptides were assigned using 1D ^1^H, 2D ^1^H,^1^H-TOCSY, 2D ^1^H,^1^H-ROESY, 2D ^1^H,^13^C-HSQC, and 2D ^1^H,^13^C-HMBC spectra. The ^1^H and ^13^C chemical shift assignments of the 8 pentapeptides at 298 K are given in Tables S1–S8 (Supporting Information File 1). A single set of chemical shifts was observed for all deprotected pentapeptides **1b**–**4b**, whereas for the Boc-protected pentapeptides **1a**–**4a**, two chemical shift sets could be detected. This chemical shift heterogeneity involved in particular the *t-*Bu protons of the Boc group and the amide proton of the residue Ala^1^. The chemical shift set of weaker intensity was assigned more easily by cooling down to 271 K because of significant broadening near room temperature. Exchange peaks were observed on ROESY spectra at 271 K (300 ms mixing time), proving that the two forms interconvert in a slow exchange regime on the ^1^H NMR time scale. This equilibrium was ascribed to the existence of the *syn*- and *anti*-rotamers of the carbamate group. The more stable forms (about 85% population at 271 K) were assigned to *anti*-rotamers based on literature results [25].

Different NMR parameters were examined to analyze backbone conformational propensities, namely ^1^H_α_ and ^13^C_α_ chemical shift deviations (CSD), vicinal ^3^*J*_HN-Hα_ coupling constants, H^α^-HN ROE correlations and temperature coefficient (Δδ_HN_/Δ*T*) of the amide protons. The ^1^H^α^ and ^13^C^α^ chemical shift deviations (CSD) from random coil values provide information on backbone conformational space for each amino acid [26–31]. The terminal Ala^1^ and Leu^5^ residues were excluded from this CSD analysis because of the absence of a neighboring residue, as well as fluorinated Thr residues because of the absence of known random coil values. The analysis of ^1^H_α_ and ^13^C_α_ CSDs for residues Val^2^ and Val^4^ in all of the eight pentapeptides (Table 1 and Table 2) supports the predominance of extended conformations, as shown by downfield shifted Hα protons (positive CSD values between 0.09 and 0.22 ppm, Table 1) and upfield shifted Cα carbons (negative CSD values in the range of −2.5 to −1.6 ppm, Table 2).

**Table 1:** ^1^Hα chemical shift deviations (CSD) of residues in pentapeptides **1a**–**4a** and **1b**–**4b** in CD_3_OH (298 K).

Peptide	Boc-protected (**1a**–**4a**)					Non-protected (**1b**–**4b**)				

	Ala^1^	Val^2^	**X^3^**	Val^4^	Leu^5^	Ala^1^	Val^2^	**X^3^**	Val^4^	Leu^5^

R-Ala-Val-Ser-Val-Leu-OMe (**1**)	−0.23	0.09	**0.01**	0.15	0.09	−0.35	0.14	**0.04**	0.15	0.08
R-Ala-Val-Thr-Val-Leu-OMe (**2**)	−0.20	0.13	**0.04**	0.13	0.11	−0.41	0.14	**0.05**	0.12	0.07
R-Ala-Val-(2*S*,3*R*)-CF_3_-Thr-Val-Leu-OMe (**3**)	−0.22	0.09	**–**	0.17	0.07	−0.33	0.14	**–**	0.18	0.07
R-Ala-Val-(2*S*,3*S*)-CF_3_-Thr-Val-Leu-OMe (**4**)	−0.19	0.13	**–**	0.15	0.09	−0.35	0.22	**–**	0.17	0.08

**Table 2:** ^13^Cα chemical shift deviations (CSD) of residues in pentapeptides **1a**–**4a** and **1b**–**4b** in CD_3_OH (298 K).

Peptide	Boc-Protected (**1a**–**4a**)					Non-Protected (**1b**–**4b**)				

	Ala^1^	Val^2^	**X^3^**	Val^4^	Leu^5^	Ala^1^	Val^2^	**X^3^**	Val^4^	Leu^5^

R-Ala-Val-Ser-Val-Leu-OMe (**1**)	−0.6	-2.0	**−1.6**	−2.1	−2.8	−2.1	−1.7	**−1.8**	−2.2	−2.8
R-Ala-Val-Thr-Val-Leu-OMe (**2**)	−0.9	−2.0	**−1.8**	−2.2	−2.9	−2.1	−1.6	**−1.9**	−2.3	−2.9
R-Ala-Val-(2*S*,3*R*)-CF_3_-Thr-Val-Leu-OMe (**3**)	−0.8	−2.5	**−**	−2.4	−2.7	−2.2	−2.0	**−**	−2.4	−2.7
R-Ala-Val-(2*S*,3*S*)-CF_3_-Thr-Val-Leu-OMe (**4**)	−0.8	−1.6	**−**	−1.9	−2.9	−2.1	−2.0	**−**	−2.4	−2.8

The high propensity for exploring extended backbone conformations was further confirmed for these pentapeptides by the analysis of Hα–HN ROE correlations, showing that sequential Hα_i_–HN_i+1_ ROEs have much higher intensities than intra-residual Hα_i_–HN_i_ ROEs. Few sequential HN–HN ROEs with weak intensities could be observed, indicating that turn or helical conformers are sparsely populated.

Because of its Karplus dependence upon main chain φ dihedral angle, the vicinal ^3^*J*_HN-Hα_ coupling constant is also a valuable descriptor of peptide backbone conformations [32]. The coupling constants in all pentapeptides (Table 3) exhibit large values (6.8–9.2 Hz range), that are systematically higher than average values found in the coil library (6.1, 7.0, and 7.5 Hz for Ala, Leu and Val, respectively) [33]. This clearly reflects a preference of all backbone dihedral angles φ for values within the range of −160° to −110°, as expected for extended conformations. The three central residues presented higher ^3^*J*_HN-Hα_ coupling constants than terminal Ala^1^ and Leu^5^ residues, thus demonstrating stronger extended conformational propensities. Interestingly, the conformation of the central residue gets more extended upon substitution of Ser (^3^*J*_HN-Hα_ of 7.5 Hz) by the β-branched Thr residue (^3^*J*_HN-Hα_ 8.4 Hz) and trifluoromethylation of Thr further stabilizes extended conformations (^3^*J*_HN-Hα_ between 8.6 and 9.1 Hz).

**Table 3:** Coupling constants ^3^*J*_HN–Hα_ (Hz) of residues in pentapeptides **1a**–**4a** and **1b**–**4b** in CD_3_OH (271 K for most residues, * indicates values measured at 298 K).

Peptide	Boc-protected (**1a**–**4a**)					Non-protected (**1b**–**4b**)				

	Ala^1^	Val^2^	**X**^3^	Val^4^	Leu^5^	Ala^1^	Val^2^	**X**^3^	Val^4^	Leu^5^

R-Ala-Val-Ser-Val-Leu-OMe (**1**)	6.8	8.2	**7.5**	8.9	7.8	–	8.3	7.6*	8.6*	7.6*
R-Ala-Val-Thr-Val-Leu-OMe (**2**)	6.9*	8.3*	8.4*	8.8*	7.8*	–	broad peak	**8.4**	8.7	7.7
R-Ala-Val-(2*S*,3*R*)-CF_3_-Thr-Val-Leu-OMe (**3**)	7.1	8.9	**9.1**	9.2	7.6	–	8.7	**9.0**	8.2	7.4
R-Ala-Val-(2*S*,3*S*)-CF_3_-Thr-Val-Leu-OMe (**4**)	6.8	7.7	**8.6**	9.0	7.7	–	8.9	**9.0**	8.9	7.6

We next examined the values of vicinal ^3^*J*_Hα-Ηβ_ coupling constants which yield information on side-chain χ1 dihedral angle space (Table 4) [34]. Most residues exhibit average values that indicate conformational equilibria between different side-chain rotamers. Notably, the (2*S*,3*S*)-CF_3_-Thr residue in peptides **4a** and **4b** has a small coupling constant, indicating a *gauche* relationship between Hα and Hβ protons. The analysis of intraresidual and sequential Hβ-HN ROEs led to the identification of the χ1 *gauche*+ (+60°) conformation as the major side-chain rotamer. As the local conformational space appears to be more restricted for both backbone and side chain of (2*S*,3*S*)-CF_3_-Thr residue, we further characterized its conformation by recording ^1^H,^19^F heteronuclear NOEs in 1D ^1^H{^19^F} and 2D ^1^H,^19^F-HOESY experiments (Figures S19 and S20, Supporting Information File 1). Heteronuclear NOEs involving the CF_3_ group confirmed the previous assignment of χ1 rotamer and revealed an i/i+2 interaction with the Ala^1^ methyl group, as expected in peptides exploring major β-strand like conformations (Figure S21, Supporting Information File 1).

**Table 4:** ^3^*J*_Hα–Hβ_ coupling constants (Hz) for peptides **1a**–**4a** and **1b**–**4b** in CD_3_OH. Coupling constants were extracted from 1D ^1^H spectra on multiplets of Hβ protons for Ala or Hα protons for other residues.

Peptide	Boc-protected (**1a**–**4a**)					Non-protected (**1b**–**4b**)				

	Ala^1^	Val^2^	**X**^3^	Val^4^	Leu^5^	Ala^1^	Val^2^	**X**^3^	Val^4^	Leu^5^

R-Ala-Val-Ser-Val-Leu-OMe (**1**)	7.1	6.7	**6/6**	6.6	5.1/10.6	7.2	6.6	**7.5/7.5**	6.6	5.3/10.3
R-Ala-Val-Thr-Val-Leu-OMe (**2**)	7.2	7.3	**4.9**	6.9	5.0/10.6	7.2	7.1	**4.6**	7.1	5.6/10.0
R-Ala-Val-(2*S*,3*R*)-CF_3_-Thr-Val-Leu-OMe (**3**)	7.3	7.1	**6.4**	6.5	6/10	7.3	7.3	**6.7**	7.0	5.3/10.3
R-Ala-Val-(2*S*,3*S*)-CF_3_-Thr-Val-Leu-OMe (**4**)	7.0	7.0	**2.5**	7.1	5.0/10.5	7.2	8.2	**2.0**	7.5	5.4/10.5

The chemical shift of amide protons generally displays a temperature dependence [35–36] which can be used to get information on the presence and the stability of hydrogen bonds [37]. In aqueous and alcoholic solvents, small negative temperature coefficients (Δδ_HN_/Δ*T* > –4.5 ppb K^−1^) usually characterize amide protons that are engaged in intramolecular hydrogen bonds, while more negative values (Δδ_HN_/Δ*T* < –6 ppb K^−1^) rather indicate that they are exposed to solvent. The analysis of the temperature coefficient of the amide bond NH protons (Δδ_HN_/Δ*T*) reveals negative values in the range of −9.0 ppb/K to −5.0 ppb/K for most protons, which indicates that they are not engaged in stable intra- (or inter-)molecular hydrogen bonds with carbonyl groups (Table 5). Interestingly, residue Val^4^ displays the smallest temperature coefficient (around –4.5 ppb K^−1^) in pentapeptides **4a** and **4b** while residue Val^2^ shows intermediate values of −5.5 and −5.9 ppb K^−1^. This i/i+2 periodicity may reflect transient intermolecular β-strand contacts involving hydrogen bonding through Val^2^ and Val^4^ residues (Figure S16, Supporting Information File 1). However, no long-range HN/HN or Hα/Hα ROEs could be detected in the 8 pentapeptides, indicating that transient intermolecular association, if any, is too fast to be detected by ROE magnetization transfer.

**Table 5:** Temperature coefficients Δδ/Δ*T* (ppb K^−1^) for HN protons in pentapeptides **1a**–**4a** and **1b**–**4b** in CD_3_OH (298 K).

Peptide	Boc-protected (**1a**–**4a**)					Non-protected (**1b**–**4b**)				

	Ala^1^	Val^2^	**X**^3^	Val^4^	Leu^5^	Ala^1^	Val^2^	**X**^3^	Val^4^	Leu^5^

R-Ala-Val-Ser-Val-Leu-OMe (**1**)	−7.8	−6.4	**−7.7**	−8.0	−7.4	−	−6.1	**−7.8**	−7.8	−7.0
R-Ala-Val-Thr-Val-Leu-OMe (**2**)	−7.4	−5.8	**−7.5**	−7.6	−7.9	−	−5.5	**−8.1**	−8.0	−7.7
R-Ala-Val-(2*S*,3*R*)-CF_3_-Thr-Val-Leu-OMe (**3**)	−6.5	−5.3	**−7.4**	−7.4	−5.0	−	−6.6	**−8.5**	−9.0	−6.0
R-Ala-Val-(2*S*,3*S*)-CF_3_-Thr-Val-Leu-OMe (**4**)	−7.4	−5.9	**−8.9**	−4.5	−9.0	−	−5.5	**−8.3**	−4.6	−7.9

In summary, the NMR analysis shows that the pentapeptides with the sequence RNH-Ala-Val-**X**-Val-Leu-OMe (X = Ser, Thr, (2*S*,3*R*)-CF_3_-Thr and (2*S*,3*S*)-CF_3_-Thr) explore predominantly extended backbone conformations in CD_3_OH. No major difference could be observed between the Boc protected pentapeptides **1a**–**4a** and their respective deprotected amine analogues **1b**–**4b**. This β-propensity can be ascribed to the presence of two Val residues, as β-branched residues are known to explore more extended conformations [38]. Such an effect is also observed for the central residue upon replacement of Ser by the β-branched Thr residue and the incorporation of a trifluoromethyl group in Thr or *allo*-Thr further increases the β-propensity of these residues. The presence of self-association involving intermolecular β-sheet formation was not detectable. Nevertheless, the unique i/i+2 periodicity of amide proton temperature coefficients in peptides **4a**–**4b** incorporating the (2*S*,3*S*)-CF_3_-threonine residue might be explained by transient intermolecular β-strand contacts.

In order to gain a more detailed insight into the structural behavior of the pentapeptides according to their central fluorinated or non-fluorinated residue, all-atom molecular dynamics (MD) simulations were performed using the GROMACS 4.5 package, with the OPLS-AA force field in combination with the SPC/E water model (for a complete description of the method, see Supporting Information File 1).

The conformational ensembles generated for each of the eight pentapeptides in water, were first characterized by the average coupling constants ^3^*J*_HN-Hα_ of their five residues and then compared to available NMR measurements at 298 K (Figure S23, Supporting Information File 1). Water solvent was chosen in order to better anticipate the peptide conformations in a solvent closer to physiological conditions. Nevertheless, we verified for compounds **2b** and **4b** that the simulations conducted in MeOH and in water were very similar (Figure S23, Supporting Information File 1). Overall, the theoretical ^3^*J*_HN-Hα_ coupling values are in fair agreement with the experimental ones, indicating that the peptide conformational ensembles were sampled quite faithfully by the MD trajectories. Excepting the first residue Ala^1^, all the theoretical coupling constants have high values above 7 Hz, confirming that the pentapeptides have locally extended backbone conformations. It could be noted that the ^3^*J*_HN-Hα_ experimental value of the central residue in compounds **3a**, **3b**, and **4b** are significantly higher than in the simulations. This discrepancy between the NMR and MD ^3^*J*_HN-Hα_ coupling values for the fluorinated central residues indicates that their conformations are less frequently extended in the simulations than in experiments. However, the ^3^*J*_HN-Hα_ coupling constants alone cannot unambiguously discriminate between α- or β-structures for each residues and, above all, cannot determine the peptide global structure. In that context, MD simulations can provide useful complementary structural information.

**Figure 2:**
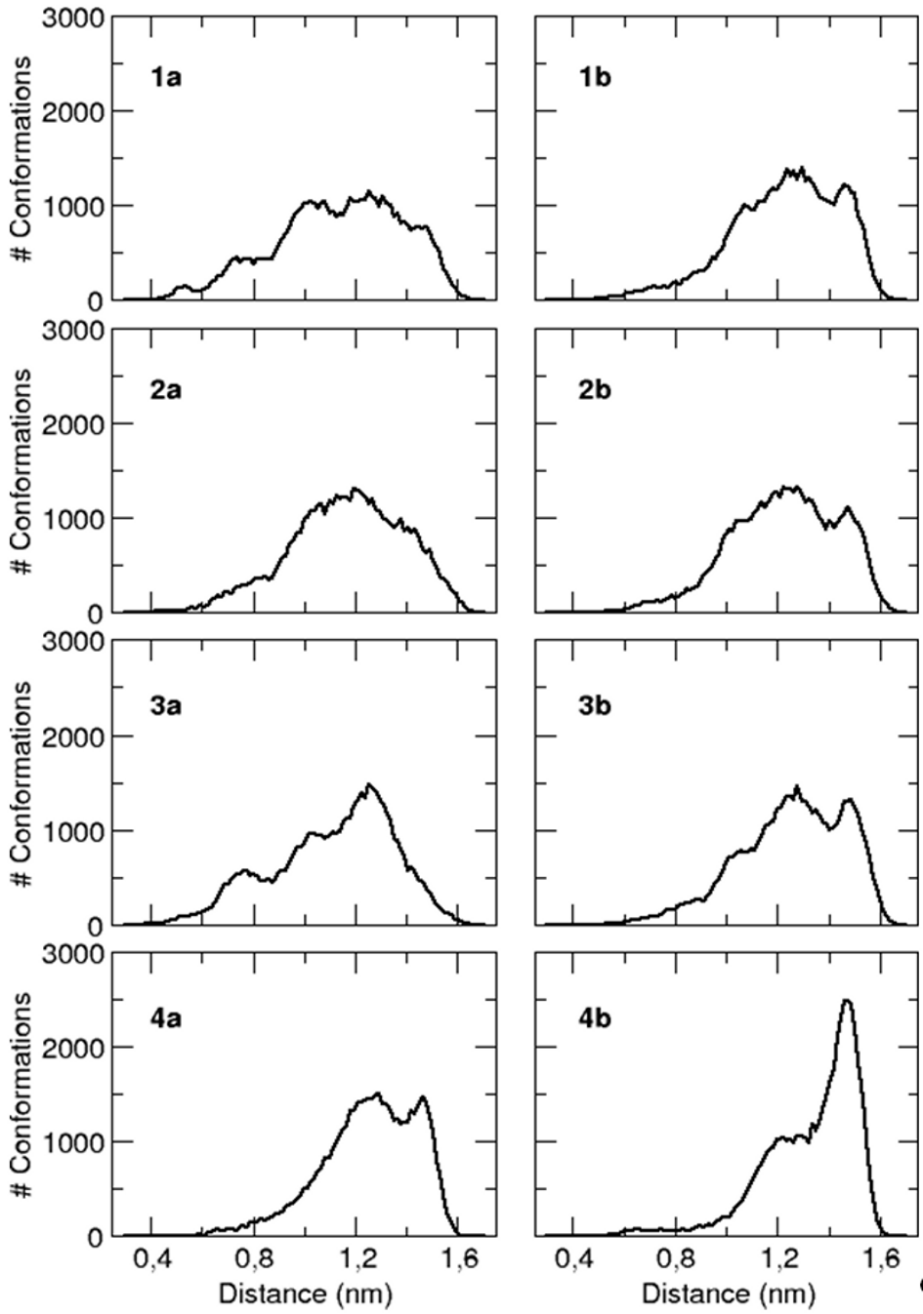
Probability distribution of the peptide conformations as a function of end-to-end distance (defined as the distance between the nitrogen of residue Ala^1^ and the carbon of the *C*-terminal carbonyl).

In particular, MD trajectories revealed significant differences between the conformations of the fluorinated and non-fluorinated peptides. Indeed, when their end-to-end distances are analyzed (Figure 2), it can be noted that both the Boc-protected and non-protected peptides **4a** and **4b** have significantly larger populations of extended conformations than the other three sequences whose distributions are broader and shifted toward lower values.

**Figure 3:**
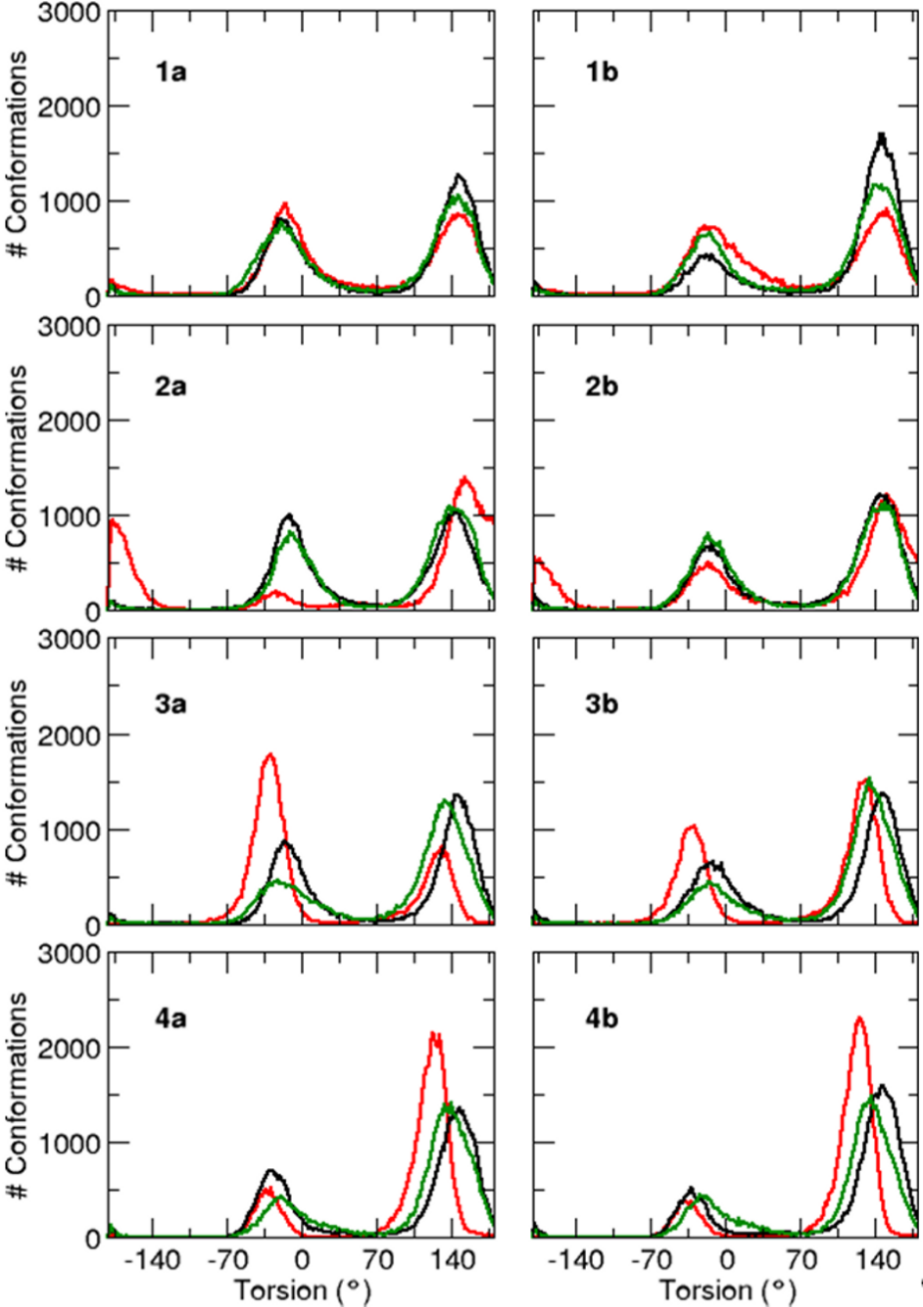
Probability distribution of the peptide dihedral angles ψ for the three central residues Val^2^ (black), X^3^ (red) and Val^4^ (green).

This global structural characteristic is reflected at a local level when the distributions of the backbone ψ dihedral angle values are examined (Figure 3). In contrast with other peptides, all the three central ψ dihedral angles of peptides **4a** and **4b** clearly have a higher propensity to populate the β basin (90° to 180°) than the α region (−70° to +40°), endowing it with the aforementioned extended conformations. More specifically, the probability of each residue to be in α- or β-conformation can be quantified by calculating the area under the peaks of the ψ distribution functions centered around −30° or +140°, respectively. The probability of the three central residues to be in β-conformation is reported in Table 6 for all studied peptides. It can be seen that, except the (2S,3*R*)-CF_3_-Thr residue in the Boc-protected peptide **3a**, all central residues predominantly adopt local β-conformations, with probabilities ranging from 50 to 92%, in agreement with the NMR CSDs and ^3^*J*_HN-Hα_ coupling constant values. The probability of each peptide to have all its three central residues in β-conformation (which is equal to the product of the three central residue probabilities) is a good indication of its propensity to adopt a global extended structure. According to this criterion, almost 50% of the **4a** and **4b** conformations are globally extended, whereas less than 30% of the other sequence conformations are in that case (Table 6).

**Table 6:** Probability (%) of the three central residues of the eight studied peptides to be in β-conformation. The column P indicates the probability for each peptide to have all its three central residues in β-conformation.

peptide	Boc-protected (**1a**–**4a**)				Non-protected (**1b**−**4b**)			

	Val^2^	**X**^3^	Val^4^	P	Val^2^	**X**^3^	Val^4^	P

RNH-Ala-Val-Ser-Val-Leu-OMe (**1**)	59.9	51.4	55.8	**17.2**	76.5	50.3	64.9	**25.0**
RNH-Ala-Val-Thr-Val-Leu-OMe (**2**)	53.5	91.1	61.0	**29.7**	64.8	73.5	59.1	**28.2**
RNH-Ala-Val-(3*R*)-CF_3_-Thr-Val-Leu-OMe (**3**)	59.7	31.7	69.9	**13.2**	63.3	59.4	74.7	**28.1**
RNH-Ala-Val-(3*S*)-CF_3_-Thr-Val-Leu-OMe (**4**)	67.1	82.7	75.6	**42.0**	77.9	85.6	74.8	**49.9**

The most prevalent conformations of each peptide were determined by clustering their conformational ensembles, using the “gromos” method implemented in GROMACS with a RMSD threshold of 0.2 nm. Visual inspections of the representative structure of the most populated clusters (Figures S24 and S25, Supporting Information File 1SI) confirm that the peptides **4a** and **4b** visit extended β-strand-like structures more frequently than the other three which have higher propensities to form compact α-helix-like conformations.

All together, the theoretical study shows that the replacement of the methyl group of the threonine side chain in the RNH-Ala-Val-Thr-Val-Leu-OMe pentapeptide by a trifluoromethyl induces an increase of the population of global extended conformations.

**Figure 4:**
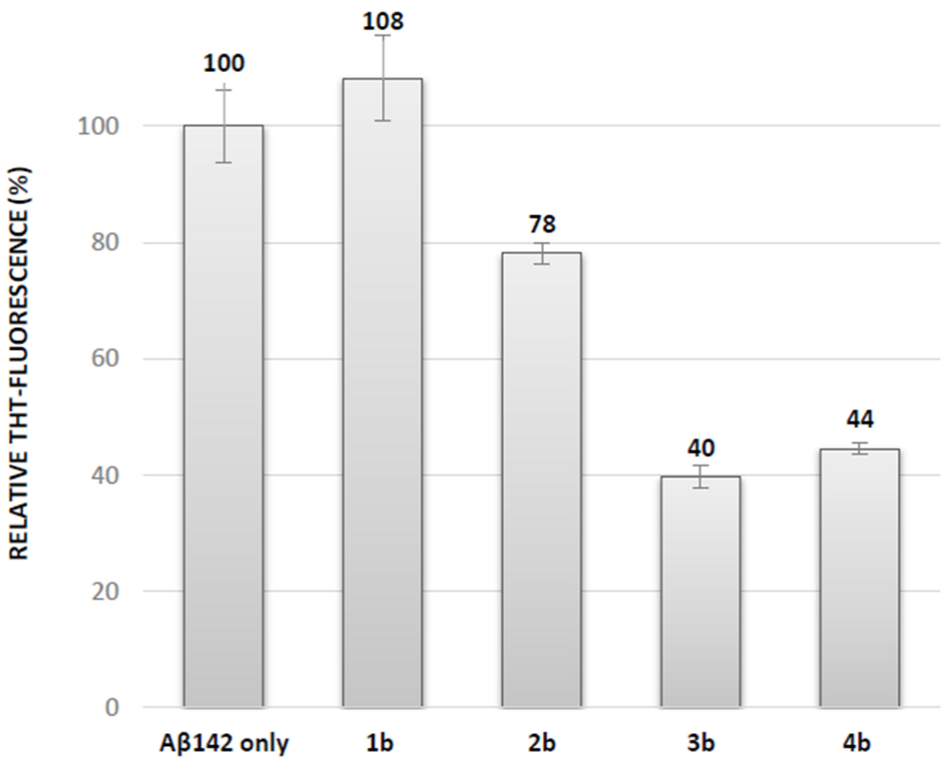
Effects of compounds **1–4** on Aβ_1-42_ fibrillization assessed by ThT-fluorescence spectroscopy at 10:1 compound/Aβ ratios (the concentration of Aβ_1-42_ is 10 μM) and compared to the values obtained for Aβ_1-42_ alone. (See Supporting Information File 1 for the calculation of the change of fluorescence intensity at the plateau).

**Inhibition of Aβ_1-42_** **fibrillization.** In the frame of our interest in modulators of protein–protein interactions involving β–sheet structures, in particular in the field of Aβ_1-42_ peptide aggregation involved in Alzheimer’s disease [15,39–42], we evaluated the activity of the pentapeptides on this process. The objective of this preliminary study was to analyze the influence of the trifluoromethyl group and of the propensity of the pentapeptides to adopt an extended structure, on their ability to modulate Aβ_1-42_ peptide aggregation. For that purpose, the classical fibrillization assay was performed using thioflavin-T (ThT) fluorescence spectroscopy [14–15,39–42]. The fluorescence curve of the control peptide (Aβ_1-42_ 10 µM, purple curve, Figure S26, Supporting Information File 1) displayed a typical sigmoid pattern with a lag phase corresponding to the nucleation process, an elongation phase and a final plateau linked to the morphology and the amount of fibrils formed at the end of the aggregation process. Compounds **1a**–**4a** and **1b**–**4b** were tested at compound/Aβ_1-42_ ratios of 10:1 and 1:1. None of the Boc-*N*-protected pentapeptides **1a**–**4a** displayed inhibitory activity even at a 10:1 compound/Aβ_1-42_ ratio (data not shown) while some *N*-deprotected compounds displayed inhibitory activity at this ratio, by decreasing the fluorescence plateau at 40 hours (see Supporting Information File 1, Table S9). This result is in accordance with our previous demonstration that a free amine is crucial to establish ionic interactions with acidic residues of Aβ_1-42_ [15,39–41]. No activity was observed at a 1:1 ratio for the fluorinated compounds **3b** and **4b**, while an increase of the fluorescence plateau was observed in the presence of the Ser and Thr containing compounds **1b** and **2b**. At a 10:1 compound/Aβ_1-42_ ratio the less extended Ser containing pentapeptide **1b** was found to be inactive (Figure 4 and Table S9, Supporting Information File 1). The Thr containing pentapeptide **2b** reduced the fluorescence plateau intensity by 22%, suggesting a slight reduction of the amount of fibrils formed after 40 hours (Figure 4 and Table S9, Supporting Information File 1). The reduction of the fluorescence intensity after 40 hours was much more pronounced for the two CF_3_-Thr derivatives **3b** and **4b**, reaching 60% (Figure 4 and Table S9, Supporting Information File 1), indicating that the presence of fluorine atoms probably increased the interaction of pentapeptides with Aβ_1-42_ and their inhibitory effect on Aβ_1-42_ aggregation.

## Conclusion

We synthesized eight pentapeptides **1a–4a** and **1b–4b** having the sequence RHN-Ala-Val-**X**-Val-Leu-OMe, where the central residue **X** is L-serine, L-threonine, (2*S*,3*R*)-L-CF_3_-threonine and (2*S*,3*S*)-L-CF_3_-threonine, respectively. The fluorinated amino acid (2*S*,3*R*)-Boc-CF_3_-Thr(Bzl) was prepared through a nucleophilic trifluoromethylation of Ruppert’s reagent on the (*R*) Garner’s aldehyde, while (2*S*, 3*S*)-Boc-CF_3_-Thr (mimic of the natural threonine) was obtained through the aldol reaction of trifluoroacetaldehyde with the Ni(II) complex of the chiral Schiff base of glycine.

The conformational analysis of these pentapeptides was conducted by the combined use of NMR spectroscopy and molecular dynamics simulations. NMR conformational studies showed that the eight pentapeptides (**1a–4a** and **1b–4b**) adopt mainly extended backbone conformations in a polar solvent (CD_3_OH). The MD simulated conformations were in fair agreement with the NMR results. Overall we conclude that the CF_3_-Thr-containing pentapeptides were experimentally found more extended than the L-Ser-, L-Thr derivatives, with the (2*S*,3*S*)-CF_3_-Thr-residue more prone to induce extended conformations than the (2*S*,3*R*)-CF_3_-Thr, as suggested by MD simulations. The temperature coefficients observed in both Boc-protected and deprotected (2*S*,3*S*)-CF_3_-Thr pentapeptides (**4a** and **4b**) suggest that these pentapeptides could transiently form intermolecular β-strand contacts. This higher propensity of **4a** and **4b** to adopt extended structures can be explained by a strong hydrophobic interaction of the trifluoromethyl group with the Ala^1^ methyl group side chain, as observed in ^1^H,^19^F heteronuclear NOEs in 1D ^1^H{^19^F} and 2D ^1^H,^19^F HOESY experiments. Thus, both conformational studies demonstrated the trifluoromethyl effect on peptide conformations that promotes an extended conformation in order to mimic a β-strand structure. Interestingly in the MD results, we found that the deprotected pentapeptides **1b**, **3b** and **4b** showed increased propensities to adopt extended conformations compared to the Boc-protected counterparts **1a, 3a** and **4a** (a similar propensity to be in β-conformation was observed for **2a** and **2b**).

The structural information obtained in this study provides valuable insights to explore novel β-strand mimics containing trifluoromethylated analogues of threonine as inhibitors of protein–protein interactions involving β-sheet structures. As a proof of concept, we demonstrated that the incorporation of the CF_3_-Thr residues in hydrophobic pentapeptides allowed their interaction with the amyloid protein Aβ_1-42,_ in order to reduce its aggregation process. The inhibitory effect seems more pronounced by combining both the use of extended pentapeptides and the introduction of fluorine atoms. This positive effect of the trifluoromethylation can be due to the increased polarity of the hydroxy group in the CF_3_-Thr residue, acting as a β-sheet breaker element and thus preventing the interactions between Aβ species [15].

The introduction of such fluorinated peptides in larger structures, such as glycopeptide or β-hairpin compounds can be envisaged. Indeed we have previously demonstrated that small peptides/peptidomimetics that displayed inhibitory activity at high ratios show greater aggregation inhibitory activity at 1:1 ratio or even less, when they are incorporated in such designed structures [15,39–41].

## Supporting Information

File 1 Description of synthetic procedures and characterization of compounds. Additional NMR data, computational methods and additional figures and tables. Experimental procedure for fluorescence-detected ThT binding assay and representative curves of ThT fluorescence assays.
