# Supplementary material for: The use of 4,4,4-trifluorothreonine to stabilize extended peptide structures and mimic β-strands
Source: Beilstein J Org Chem. 2017 Dec 21;13:2842–53. doi: 10.3762/bjoc.13.276 (PMC5753055; doi:10.3762/bjoc.13.276)
Supplement: File 1 — Description of synthetic procedures and characterization of compounds. Additional NMR data, computational methods and additional figures and tables. Experimental procedure for fluorescence-detected ThT binding assay and representative curves of ThT fluorescence assays. [file Beilstein_J_Org_Chem-13-2842-s001.pdf]

Supporting Information
for

The use of 4,4,4-trifluorothreonine to stabilize extended peptide structures and mimic β-strands

Yaochun Xu[1], Isabelle Correia[2], Tap Ha-Duong[1], Nadjib Kihal[1], Jean-Louis Soulier[1], Julia Kaffy[1], Benoît Crousse[1], Olivier Lequin*[2] and Sandrine Ongeri*[1]

Address: [1]Molécules Fluorées et Chimie Médicinale, BioCIS, Univ. Paris-Sud, CNRS, Université Paris Saclay, 5 rue Jean-Baptiste Clément, 92296 Châtenay-Malabry Cedex, France and [2]Sorbonne Universités, UPMC Univ Paris 06, Ecole Normale Supérieure, PSL Research University, CNRS, Laboratoire des Biomolécules, 4 place Jussieu, 75252 Paris Cedex 05, France.

Email: Sandrine Ongeri - Sandrine.ongeri@u-psud.fr; Olivier Lequin olivier.lequin@upmc.fr

*Corresponding author

Description of synthetic procedures and characterization of compounds. Additional NMR data, computational methods and additional figures and tables. Experimental procedure for fluorescence-detected ThT binding assay and representative curves of ThT fluorescence assays.

Table of contents

Page S3 Synthesis of compounds **1a**–**4a**, **1b**–**4b**, **6–18c**
Page S16 ^{19}F and ^{1}H NMR spectra of **7a**
Page S17 NMR data for the conformational studies of pentapeptides **1a–4a** and **1b–4b**
Page S36 Molecular dynamics studies: experimental procedure and data
Page S39 Experimental procedure for fluorescence-detected thioflavin-T binding assay (Aβ$_{1-42}$).
Page S40 Representative curves of Tht fluorescence assays over time showing Aβ$_{1-42}$ aggregation in presence of compounds **1b–4b**
Page S41 References

Synthesis of Compounds 1a–4a, 1b–4b, 6–18c

Usual solvents were purchased from commercial sources. Dimethylformamide (DMF) was distilled over $CaSO_4$, tetrahydrofuran (THF) was distilled over sodium/benzophenon. Protected amino acids, (*R*)-(+)-3-Boc-2,2-dimethyloxazolidine-4-carboxaldehyde ((*R*)-Gardner's aldehyde), O-benzotriazol-1-yl-*N*,*N*,*N*,*N*'-tetramethyuronium hexafluorophosphate (HBTU), 4-(4,6-dimethoxy-1,3,5-triazin-2-yl)-4-methylmorpholinium chloride (DMTMM(Cl^-)), and 1-hydroxybenzotriazole (HOBt) were purchased from commercial sources. Pure products were obtained after liquid chromatography using Merck silica gel 60 (40−63 µm). TLC analyses were performed on silica gel 60F-250 (0.26 mm thickness) plates. The plates were visualized with UV light (λ = 254 nm) or revealed with a 5% solution of phosphomolybdic acid in EtOH or with a solution of ninhydrin in EtOH. Melting points were determined on a Kofler melting point apparatus. Element analyses (C, H, and N) were performed on a PerkinElmer C,H,N Analyzer 2400 at the Microanalyses Service of the Faculty of Pharmacy at Châtenay-Malabry (BioCIS, France). NMR spectra were recorded on an Ultrafield Bruker AVANCE 300 (^{1}H, 300 MHz, ^{13}C, 75 MHz), a Bruker ARX 200 (^{19}F, 188 MHz), a Bruker Avance 400 (^{1}H, 400 MHz, ^{13}C, 100 MHz, ^{19}F, 376 MHz), or a Bruker Avance 500 MHz equipped with a TCI cryoprobe for the assignment of the ^{1}H and ^{13}C chemical shifts and for the conformational studies of pentapeptides **1a**–**4a** and **1b**–**4b**. ^{1}H and ^{13}C resonances were assigned using 1D ^{1}H WATERGATE, 2D ^{1}H-^{1}H TOCSY (MLEV17 isotropic scheme of 66 ms duration), 2D ^{1}H-^{1}H ROESY (300 ms mixing time), 2D ^{1}H-^{13}C HSQC and 2D ^{1}H-^{13}C HMBC spectra. 2D ^{1}H-^{19}F HOESY experiment was recorded with a mixing time of 800 ms. 1D ^{1}H{^{19}F} NOE difference experiment was recorded by using an irradiation scheme (1 ms Gaussian pulses, total duration 1 s) applied alternatively on and off resonance. ^{1}H and ^{13}C chemical shifts were calibrated using the solvent residual peak (CHD_2OH, δ ^{1}H 3.31 ppm, δ ^{13}C 49.5 ppm). The chemical shift deviations were calculated as the differences between observed chemical shifts and random coil values reported in water [1-6]. The temperature gradients of the amide proton chemical shifts were derived from 1D ^{1}H WATERGATE spectra recorded over a 25°C interval. $^3J_{HN\text{-}H\alpha}$ coupling constants were measured on 1D ^{1}H WATERGATE. Chemical shifts δ are in ppm, and the following abbreviations are used: singlet (s), doublet (d), doublet of doublet (dd), triplet (t). IR spectra were recorded on a Bruker Vector 22 FT-IR spectrometer. Mass spectra were obtained using a Bruker Esquire electrospray ionization apparatus. HRMS were obtained using a TOF LCT Premier apparatus (Waters), with an electrospray ionization source. The purity of compounds was determined by HPLC using the 1260 Infinity system (Agilent Technologies) and a Sunfire column (C_{18}, 3.5 µm, 100 mm × 2.1 mm); mobile phase, MeCN/H_2O + 0.1% formic acid from 5 to 100% in 20 min; detection at 254 nm; flow rate 0.25 mL/min. In the case of **1a**, hydrogenation was performed on an H-Cube HC-2SS Thales nanotechnology device with Pd 10% CatCart cartridge.

General procedure A for deprotection reactions of Boc protected peptides: To a solution of the *N*-Boc-protected peptide in CH_2Cl_2 (0.13 M) was added a third volume of TFA. Once the end of reaction was monitored by TLC, solvent was removed under vacuum and toluene (2×) was added followed by evaporation. Et_2O was then added and filtration of the solid afforded the corresponding TFA salt.

General procedure B for coupling reactions: To the solution of Boc-protected amino acid (1 equiv), in dry DMF (0.28 M) at 0 °C under nitrogen atmosphere, was successively added DIPEA (5 equiv), HOBt (1.1 equiv), and HBTU (1.1 equiv). The solution was stirred at 0 °C for 1 h and then the solution of the TFA salt of the peptide, prepared according to method A, was added to the reaction mixture. The reaction was further stirred at 0°C for 30 minutes to 1 h and at room temperature overnight. The solvent was evaporated under vacuum and the residue was taken up with EtOAc or CH_2Cl_2. The organic phase was successively washed with 10% aqueous citric acid (50 mL), 10% aqueous K_2CO_3 (50 mL) and brine (20 mL), dried over Na_2SO_4, filtered and concentrated under vacuum to afford the crude product which was purified by column chromatography or by crystallization.

General procedure C for coupling reactions: To a solution of Boc-protected amino acid (1 equiv) in dry DMF or CH_2Cl_2 (0.06 M), under nitrogen atmosphere and at 0 °C, were added DMTMM(Cl^-) (1,2 equiv) and NMM (6 equiv). After 30 min, a solution of the TFA salt of the peptide (1 equiv), prepared according to method A, in DMF or CH_2Cl_2 was added and the reaction mixture was stirred at 0 °C for 1 h and at room temperature overnight. The solvent was evaporated under vacuum and the residue was taken up with EtOAc or CH_2Cl_2. The organic phase was successively washed with 10% aqueous citric acid (50 mL), 10% aqueous K_2CO_3 (50 mL) and brine (20 mL), dried over Na_2SO_4, filtered and concentrated under vacuum to afford the crude product which was purified by column chromatography or by crystallization.

(*S*)-2-((*S*)-2-{(*S*)-2-[(*S*)-2-((*S*)-2-(*tert*-Butoxycarbonylamino)propionylamino)-3-methylbutyrylamino]-3-hydroxypropionylamino}-3-methylbutyrylamino)-4-methylpentanoic acid methyl ester (1a). Once H-Cube system equipped with a 10% Pd/C CatCartridge (setting the temperature to 20 °C and the hydrogen pressure to 20 bar), **18a** was dissolved in MeOH/DMF (2/0.2, v/v, 1mL) and the solution was pumped through the H-Cube system with a flow rate of 0.5 mL/min. After three passages, the solvent was removed under vacuum to yield **1a** as a white solid (0.04 g, 0.07 mmol, 92%); Mp = 226-228 °C; ^1H and ^{13}C NMR see Table S1; IR (neat): ν_{max}: 3280, 2963, 16312, 1525, 1366, 1230, 1159 cm^{-1}; HRMS (TOF ESI, ion polarity positive): m/z calcd for $C_{28}H_{51}N_5O_9$: $[M+Na]^+$: 624.3584; found: 624.3583. $C_{28}H_{51}N_5O_9$: cacld C% 55.89, H% 8.54, N% 11.64, found C% 55.49, H% 8.26, N% 11.62.

(*S*)-2-((*S*)-2-{(*S*)-2-[(*S*)-2-((*S*)-2-Aminopropionylamino)-3-methylbutyrylamino]-3-hydroxy-propionylamino}-3-methylbutyrylamino)-4-methylpentanoic acid methyl ester hydrochloride salt (1b). A solution of the pentapeptide **1a** (30 mg, 0.05 mmol) was dissolved in CH_2Cl_2 (1 mL) at 0 °C, then a solution HCl 4 M in dioxane (0.60 mL, 2.24 mmol) was added. The reaction mixture was stirred at 0 °C for 30 min, then at room temperature for 2 hours. The solvents were evaporated under vacuum, and then a little amount of Et_2O was added to the crude residue to precipitate the salt. Filtration and precipitation with Et_2O was repeated several times. The resulting hydrochloride salt **1b** (27 mg, 0.05 mmol) was obtained as a white solid in a quantitative yield; ^{1}H and ^{13}C NMR see Table S2; IR (neat): v_{max}: 3279, 2959, 1746, 1634, 1548, 1469, 1390, 1223, 1157 cm^{-1}; HRMS (TOF ESI, ion polarity positive): m/z calcd for $C_{23}H_{44}N_5O_7$: $[M+H]^+$: 502.3241; found: 502.3230; $C_{23}H_{44}ClN_5O_7$**.**$2.5H_2O$: cacld C% 47.37, H% 8.49, N% 12.01, found C% 47.46, H% 8.00, N% 11.73.

(*S*)-2-((*S*)-2-{(2*S*,3*R*)-2-[(*S*)-2-((*S*)-2-(*tert*-Butoxycarbonylamino)propionylamino)-3-methyl-butyrylamino]-3-hydroxybutyrylamino}-3-methylbutyrylamino)-4-methylpentanoic acid methyl ester (2a). To a stirred solution of the pentapeptide **18b** (100 mg, 0.142 mmol) in CH_3OH/DMF (5:0.5, v/v, 5.05 mL) was added Pd/C (20 mg, 20% mass). The reaction flask was purged three times with hydrogen, and stirring was maintained under hydrogen atmosphere at room temperature for 20 hours. Upon completion of the reaction monitored by TLC, the solution was filtered through a pad of Celite which was washed several times with CH_3OH. Then, the filtrate was concentrated under reduced pressure to provide **2a** as a white-yellowish solid in a quantitative yield; Rf (EtOAc/Cyclohexane, 70/30) = 0.10; ^{1}H and ^{13}C NMR see Table S3; IR (neat): v_{max}: 3368, 2982, 2929, 1670, 1532, 1456, 1370, 1267, 1136 cm^{-1}; HRMS (TOF ESI, ion polarity positive): m/z calcd for $C_{29}H_{53}N_5O_9Na$ $[M+Na]^+$: 638.3741, found: 638.3748; m/z calcd for $C_{29}H_{54}N_5O_9$ $[M+H]^+$: 616.3922, found: 616.3935.

(*S*)-2-((*S*)-2-{(2*S*,3*R*)-2-[(*R*)-2-((S)-2-Aminopropionylamino)-3-methyl-butyrylamino]-3-hydroxy-butyrylamino}-3-methylbutyrylamino)-4-methylpentanoic acid methyl ester trifluoroacetic salt (2b). Prepared according to the general method A, from **2a** (45 mg, 0.073 mmol), to afford **2b** as a white solid in a quantitative yield; ^{1}H and ^{13}C NMR see Table S4; IR (neat): v_{max}: 3278, 2963, 1633, 1544, 1201, 1138 cm^{-1} HRMS (TOF ESI, ion polarity positive): m/z calcd for $C_{24}H_{45}N_5O_7Na$ $[M+Na]^+$: 538.3217, found: 538.3220; m/z calcd for $C_{24}H_{46}N_5O_7$ $[M+H]^+$: 516.3397, found: 516.3384**.**

(*S*)-2-((*S*)-2-{(2*S*,3*R*)-2-[(*S*)-2-((*S*)-2-(*tert*-Butoxycarbonylamino)propionylamino)-3-methyl-butyrylamino]-4,4,4-trifluoro-3-hydroxybutyrylamino}-3-methylbutyrylamino)-4-methylpentanoic

acid methyl ester (3a).Compound **3a** was synthesized following the same procedure as described for **2a** from **18c** (0.06 g, 0.079 mmol) in (2:0.2, v/v, 1 mL) and using $Pd(OH)_2$ (40 mg, 20% mass) to afford **3a** as a white solid (0.03 g, 0.045 mmol, 60%); ^{1}H and ^{13}C NMR see Table S5; ^{19}F NMR (188 MHz, CD_3OD): -77.83 (d, *J* = 5.7 Hz) IR (neat): ν_{max}: 3258, 2956, 2900, 1637, 1544, 1366, 1172 cm^{-1}; HRMS (TOF ESI, ion polarity positive): m/z calcd for $C_{29}H_{50}F_3N_5O_9Na$ $[M+Na]^+$: 692.3458, found: 692.3463;

(*S*)-2-((*S*)-2-{(2*S*,3*R*)-2-[(*S*)-2-((*S*)-2-Aminopropionylamino)-3-methylbutyrylamino]-4,4,4-trifluoro-3-hydroxybutyrylamino}-3-methylbutyrylamino)-4-methylpentanoic acid methyl ester hydrochloride salt (3b). Compound **3b** was synthesized following the same procedure as described for **1b** from **3a** (9 mg, 0.013 mmol) to afford **3b** (8 mg, 0.013 mmol) as a white solid in a quantitative yield; ^{1}H and ^{13}C NMR see Table S6; ^{19}F NMR (188 MHz, CD_3OD): -77.34; IR (neat): ν_{max}: 2956, 2926, 2856, 1724, 1668, 1456, 1366, 1260, 1125, 1075 cm^{-1}; HRMS (TOF ESI, ion polarity positive): m/z calcd for $C_{24}H_{43}F_3N_5O_7$: $[M+H]^+$: 570.3115; found: 570.3115 ;m/z calcd for $C_{24}H_{42}F_3N_5O_7Na$: $[M+Na]^+$: 592.2934; found: 592.2941.

(*S*)-2-((*S*)-2-{(2*S*,3*S*)-2-[(*S*)-2-((*S*)-2-(*tert*-Butoxycarbonylamino)propionylamino)-3-methyl-butyrylamino]-4,4,4-trifluoro-3-hydroxybutyrylamino}-3-methylbutyrylamino)-4-methylpentanoic acid methyl ester (4a). Tripeptide **17d** (57 mg, 0.11 mmol) was deprotected according to general method A. The resulting salt was then coupled to Boc-Ala-OH (22 mg, 0.12 mmol), according to the general method C, to afford **4a** (61 mg, 0.09 mmol, 83%) as a white solid after purification by column chromatography on silica gel using using CH_2Cl_2/MeOH (98:2) as eluent; Rf (CH_2Cl_2/MeOH, 95:5) = 0.20; ^{1}H and ^{13}C NMR see Table S7; ^{19} F NMR (188 MHz, CD_3OD): -80.28 (*J* = 7.6 Hz); IR (neat): ν_{max}: 3278, 2962, 2928, 1749, 1639, 1547, 1458, 1367, 1162 cm^{-1}; HRMS (TOF ESI, ion polarity positive): m/z calcd for $C_{29}H_{50}F_3N_5O_9Na$ $[M+Na]^+$: 692.3458, found: 692.3461; $C_{29}H_{50}F_3N_5O_9.H_2O$: cacld C% 50.64, H% 7.64, N% 10.19, found C% 50.66, H% 7.26, N% 9.75

(*S*)-2-((*S*)-2-{(2*S*,3*S*)-2-[(*S*)-2-((*S*)-2-Aminopropionylamino)-3-methylbutyrylamino]-4,4,4-trifluoro-3-hydroxybutyrylamino}-3-methylbutyrylamino)-4-methylpentanoic acid methyl ester hydrochloride salt (4b). Compound **4b** was synthesized following the same procedure as described for **1b** from **4a** (25 mg, 0.037 mmol), to afford **4b** (23 mg, 0.037 mmol, quantitative yield) as a white solid; ^{1}H and ^{13}C NMR see Table S8; ^{19}F NMR (188 MHz, CD_3OD): -78.65 (d, *J* = 7.5 Hz); IR (neat): ν_{max}: 3285, 2962, 1748, 1636, 1554, 1455, 1186, 1138 cm^{-1}; HRMS (TOF ESI, ion polarity positive): m/z calcd for $C_{24}H_{43}F_3N_5O_7$ $[M+H]^+$: 570.3115, found: 570.3105; m/z calcd for $C_{24}H_{42}F_3N_5O_7Na$ $[M+H]^+$: 592.2934, found:

592.3028; $C_{24}H_{43}ClF_3N_5O_7$.$2H_2O$: cacld C% 44.89, H% 7.39, N% 10.91, found C% 44.69, H% 7.13, N% 10.85.

(*R*)-2,2-Dimethyl-4-(2,2,2-trifluro-1-hydroxyethyl)oxazolidine-3-carboxylic acid *tert*-butyl ester (6). To a cold solution of (*R*)-Garner's aldehyde (1 g, 4.36 mmol) at 0 °C in THF (5 mL) was added a TMS·CF_3 solution 2 M in THF (2.6 mL, 17.45 mmol). After stirring 2 h at room temperature, a TBAF solution 1.1 M in THF was added (0.1 mL, 0.35 mmol) then after 60 h of stirring was added again a TBAF solution 1.1 M in THF (8.80 mL, 30.53 mmol). The reaction mixture was let under agitation for just 6 h at room temperature and stopped by adding saturated aqueous $NaHCO_3$ (100 mL) and immediately after taken up with EtOAc (300 mL). The organic phase was successively washed with saturated aqueous $NaHCO_3$ (50 mL), distilled water (50 mL) and brine (50 mL), then dried over Na_2SO_4, filtered and concentrated under vacuum. The crude residue obtained was purified by column chromatography on silica gel using cyclohexane/EtOAc 92:8, then 80:20, and finally 70:30 as eluent to afford compound **6** (0.81 g, 2.71 mmol , 62%) as a white solid made of a mixture of two diasteroisomers with a ratio of 9:1 (determined by ^{19}F NMR). Rf (EtOAc/MeOH , 90:10) = 0.75; ^{1}H NMR (300 MHz, $CDCl_3$): 4.33-3.91 (m, 5H), 1.52-1.40 (m, 15H), the existence of several rotamers made it difficult to interpret the spectrum [7];^{19}F NMR (188 MHz, $CDCl_3$):-76.92 (d, *J* = 7.5 Hz), -77.68 (d, *J* = 7.5 Hz) with a ratio 9:1. MS (ESI, ion polarity negative) m/z : 298 $[M-H]^-$.

(*R*)-4-((*R*)-1-Benzyloxy-2,2,2-trifluoroethyl)-2,2-dimethyloxazolidine-3-carboxylic acid *tert*-butyl ester (7a). To a solution of compound **6** in anhydrous DMF (10mL) at 0° C was added a suspension of NaH (0.61g, 60% in mineral oil, 8 mmol). Then, benzyl bromide (0.63 mL, 5.35 mmol) was added dropwise. The reaction mixture was stirred overnight at room temperature and stopped by adding water (20 mL) and immediately after taken up with EtOAc (30 mL). The organic phase was washed with brine (30 mL) then dried over Na_2SO_4, filtered and concentrated under vacuum. The crude oil obtained was purified by column chromatography on silica gel using Cyclohexane/EtOAc 95:2 as eluent to afford separately the two diasteroisomers **7a** (0.67 g, 1.72 mmol, 64%), and **7b** (0.06 g, 0.15 mmol, 6%), as two white solids. Rf (cyclohexane/EtOAc, 60:40) = 0.75 for **7a** and 0.65 Rf (Cyclohexane/EtOAc, 60/40) = 0.65 for **7b**.

It should be noted that only diasteroisomer **7a** was used in the following steps of the synthesis, so we give only the NMR description of this diastereoisomer. ^{1}H NMR (300 MHz, $CDCl_3$): 7.41-7.31 (m, 5H), 4.94-4.60 (m, 2H), 4.20 (m, 3H), 4.02 (d, *J* = 7.6 Hz, 1H), 1.61-1.46 (m, 15H); ^{19}F NMR (188 MHz, $CDCl_3$): -74.45 (d, *J* = 7.5 Hz), -74.68 (d, *J* = 7.5 Hz) ; as two rotamers : 58/42 (%).The presence of

rotamers has been confirmed by ^{19}F NMR experiment (Fig.S1, SI). These rotamers disappeared by heating the fluor device at different temperatures or by changing the NMR solvent.

((1*R*,2*R*)-2-(Benzyloxy)-3,3,3-trifluoro-1-hydroxymethylpropyl)carbamic acid *tert*-butyl ester (8). To a solution of compound **7a** in anhydrous MeOH (50 mL) was added p-toluene sulfonic acid (0.043 g, 0.25 mmol). The reaction mixture was stirred for 5 days at room temperature. After concentration under vacuum, the resulting residue was taken up with EtOAc (30 mL) and washed successively with saturated aqueous K_2CO_3 (30 mL), distilled water (30 mL) and brine (30 mL), then dried over Na_2SO_4, filtered and concentrated under vacuum. The crude residue obtained was purified by column chromatography on silica gel using cyclohexane/EtOAc 85:15 as eluent to afford **8** (0.31 g, 0.89 mmol, 50%) as a white solid. Rf (EtOAc/Cyclohexane), 98:2) = 0.80; ^{1}H NMR (300 MHz, $CDCl_3$): 7.45-7.32 (m, 5H), 5.20 (bs, 1H), 4.91 (d, *J* = 11.2 Hz, 1H), 4.62 (d, *J* = 11.2 Hz, 1H), 4.18 (m, 1H), 4.05 (dd, *J* = 11.9, 3.0 Hz, 1H), 3.87 (dd, *J* = 11.9, 3.0 Hz, 1H), 3.74-3.64 (m, 1H), 2.25 (bs, 1H); 1.44 (s, 9H); ^{13}C NMR(75 MHz, $CDCl_3$): 157.5, 137.1, 128.8, 128.6, 128.4, 126.6, 78.1, 77.7, 75.7, 65.8, 61.8, 50.5, 28.2, 15.2; ^{19}F NMR (188MHz, $CDCl_3$): -73.40 (d, *J* = 7.5 Hz).

(2*S*,3*R*)-3-Benzyloxy-2-(*tert*-butoxycarbonylamino)-4,4,4-trifluoro-butyric acid (9). To a solution of compound **8** (0.29 g, 0.83 mmol) in acetone (50 mL) at 0° C was added a fresh Jones reagent (1.20 mL). The reaction mixture was stirred 3 h at 0 °C and stopped by adding isopropanol (5 mL) and immediately taken up with EtOAc (30 mL). The organic phase was successively washed with distilled water (30 mL), brine (30 mL) then dried over Na_2SO_4, filtered and concentrated under vacuum to yield **9** as a white solid (0.27 g, 0.74 mmol, 90%). Rf (EtOAc/MeOH, 98:2) = 0.70; ^{1}H NMR (400 MHz, CD_3OD): 7.43-7.25 (m, 5H), 4.81(d, *J* = 11.0 Hz, 1H), 4.72 (d, *J* = 11.0 Hz, 1H), 4.57 (d, *J* = 5.7 Hz, 1H), 4.37 (m, 1H), 1.45 (s, 9H); ^{13}C NMR (100 MHz, CD_3OD): 175.3, 157.5, 138.5, 129.3, 129.2, 129.0, 126.2 (q, *J* = 283.5 Hz, $\underline{C}F_3$), 80.7, 78.6 (q, *J = 28.5* Hz, $C\underline{H}CF_3$), 75.17, 55.7, 28.7; ^{19}F NMR (188 MHz, CD_3OD): -74.31 (d, *J* = 5.6 Hz); IR (neat): ν_{max}: 3531, 3377, 2982, 2189, 1732, 1608, 1480 cm^{-1}; $[\alpha]_D^{20}$ = - 13 (c = 0.75, MeOH); MS (ESI, ion polarity negative) m/z : 362 $[M-H]^-$.

(*S*)-1-Benzylpyrrolidine-2-carboxylic acid (10). To a stirred solution of (L)-proline (5.0 g, 43.4 mmol) and KOH (7.3 g, 130.3 mmol) in iPrOH (30 mL), BnBr (6.2 mL, 8.9 g, 52.1 mmol) was added dropwise at 40 °C. After the addition completed (~3 h), the stirring was continued overnight at 40 °C. The reaction mixture was neutralized by adding concentrated aqueous HCl (about 2 mL) to reach pH 5–6, and then CH_2Cl_2 (15 mL) was added to the reaction mixture with stirring. The mixture was left overnight without stirring. After filtration of the KCl salt formed and evaporation of the CH_2Cl_2, acetone (30 mL) was added to the residue obtained. After a slow precipitation, compound **10** was

filtered and dried over P_2O_5 under vacuum and obtained as a white solid (6.31g, 30.8 mmol, 71%). Mp = 178-180 °C; ^{1}H NMR (300 MHz, DMSO-d_6): 7.47-7.36 (m, 5H), 4.24 (d; J = 13.2 Hz, 1H), 3.99 (d, J = 13.2Hz, 1H), 3.69 (dd, J = 9.0, 6.2 Hz, 2H), 3.29–3.13 (m,1H), 2.86 (dd, J = 17.0, 9.2 Hz, 1H), 2.29-2.19 (m, 1H), 1.94-1.75 (m, 3H); ^{13}C NMR (75 MHz, CD_3OD): 172.7, 132.1, 131.8, 131.1, 130.4, 69.5, 59.6, 55.5, 29.9, 23.9; HRMS (TOF ESI, ion polarity positive): calcd for $C_{12}H_{16}NO_2$ $[M+H]^+$: 206.1181, found: 206.1183; m/z calcd for $C_{12}H_{15}NO_2Na$ $[M+Na]^+$: 228.1000, found: 228.0999.

(*S*)-1-Benzylpyrrolidine-2-carboxylic acid (2-benzoylphenyl)amide (11). To a stirred solution of compound **10** (4.11 g, 20 mmol) in CH_2Cl_2 (10 mL) was slowly added over a period of 10 min at –20 ^{o}C $SOCl_2$ (2.85 g, 24 mmol, 1.8 mL). The stirring was continued at −10 °C until the reaction mixture became almost transparent. At this moment, a solution of 2-aminobenzophenone (3.94 g, 20 mmol) in CH_2Cl_2 (10 mL) was added to the reaction mixture at −30 °C and 5 min later, NEt_3 (6.6 mL) was then introduced. The stirring was continued at room temperature for 20 hours. After adding 10% aqueous Na_2CO_3 (30 mL) to the reaction mixture at 0 °C, the organic layer was separated and the aqueous layer extracted with CH_2Cl_2 (2 × 50 mL). The organic phases were combined, dried over Na_2SO_4, filtered and concentrated under reduced pressure. The crude residue obtained was purified by column chromatography on silica gel using cyclohexane/EtOAc 95:5 as eluent to afford **11** (5.72 g, 14.9 mmol, 77%) as a yellow solid. Mp = 96-98 ^{o}C; Rf (cyclohexane/EtOAc, 6:3) = 0.55; ^{1}H NMR (300 MHz, $CDCl_3$): 11.54 (s, 1H), 8.58 (d, J = 8.2Hz, 1H), 7.80-7.06 (m, 14H), 3.84 (d; J = 13.2 Hz, 1H), 3.54 (d, J = 13.2Hz, 1H)3.35-3.22 (m, 2H), 2.41 (dd, J =16.4, 9.0 Hz, 1H), 2.30-2.24 (m, 1H), 2.00-1.78 (m, 2H); ^{13}C NMR (75 MHz, $CDCl_3$): 198.1, 174.8, 139.3, 138.6, 138.2, 133.5, 132.7, 132.6, 130.2, 129.2, 128.4, 128.3, 127.2, 125.4, 122.3, 121.6, 68.4, 59.9, 54.0, 31.1, 24.3; $[\alpha]_{589}^{18}$ = -125.7 (c, 0.5, MeOH) (literature report [8,9] $[\alpha]_{589}^{25}$= -134.5); HRMS (TOF ESI, ion polarity positive): calcd for $C_{25}H_{25}N_2O_2$ $[M+H]^+$: 385.1916, found: 385.1917.

Complex 12. A solution of KOH (8.4 g, 150 mmol) in MeOH (20 mL) was poured into a stirred mixture of **11** (5.76 g, 15 mmol), $Ni(NO_3)_2{\cdot}6H_2O$ (8.72 g, 30 mmol), and glycine (5.63 g, 75 mmol) in MeOH (50 mL), under argon, at 60 °C. The resulting mixture was stirred at 60 °C for 2 hours and then neutralized with AcOH (16 mL). After concentration under vacuum, the residue was taken up with EtOAc (50 mL) and 10% aqueous $NaHCO_3$ (50 mL). The organic phase was separated, washed with brine (50 mL), dried over Na_2SO_4, filtered and concentrated under vaccum. The crude residue was purified by column chromatography on silica gel using EtOAc/MeOH 91:9 as eluent to give the Ni(II) complex **12** (5.34 g, 10.7 mmol, 71%) as a red solid. Mp = 218-220 ^{o}C; Rf (EtOAc/MeOH, 95/5) = 0.2;

^{1}H NMR (300 MHz, $CDCl_3$): 8.27 (d, *J* = 8.6 Hz, 1H), 8.07 (d, *J* = 7.0 Hz, 2H), 7.50 (m, 3H), 7.42 (t, *J* = 7.5 Hz, 2H), 7.31 (m, 1H), 7.20 (ddd, *J* = 8.7, 6.9, 1.7 Hz, 1H), 7.09 (d, *J* = 7.9 Hz, 1H), 6.97 (m, 1H), 6.79 (dd, *J* = 8.2, 1.7 Hz, 1H), 6.73–6.65 (m, 1H), 4.47 (d, *J* = 12.6 Hz, 1H), 3.82–3.62 (m, 4H), 3.47 (dd, *J* = 10.7, 5.5 Hz, 1H), 3.34 (m, 1H), 2.62–2.35 (m, 2H), 2.14 (m, 2H); ^{13}C NMR (75 MHz, $CDCl_3$): 181.5, 177.5, 171.8, 142.6, 134.7, 133.4, 133.3, 132.3, 131.8, 129.9, 129.7, 129.5, 129.2, 129.0, 126.4, 125.8, 125.3, 124.4, 121.0, 70.0, 63.3, 61.4, 57.6, 30.8, 23.8; $[\alpha]_{589}^{19}$= +2160 (c, 1.0, MeOH) (literature report [8,9] $[\alpha]_{589}^{25}$= +2006); HRMS (TOF ESI, ion polarity positive): calcd for $C_{27}H_{26}N_3NiO_3$ $[M+H]^+$: 498.1328, found: 498.1348; m/z calcd for $C_{27}H_{25}N_3NiO_3Na$ $[M+Na]^+$: 520.1147, found: 520.116

Complex 13. To the mixture of Ni(II) complex **12** (5.12 g, 10 mmol) and MeONa (6.7 mL, 36 mmol) in MeOH (15 mL) was added CF_3CHO (4.0 mL, 15 mmol). The reaction mixture was heated and refluxed for 1 hour, and then was quenched with AcOH (4 mL) in H_2O (10 mL). After concentration under vacuum, the crude residue obtained was taken up with EtOAc (2 × 50 mL) and the organic phase was successively washed with 10% aqueous $NaHCO_3$ (50 mL) and brine, dried over Na_2SO_4, and filtered. After concentration under vacuum, the residue was purified by column chromatography on silica gel using EtOAc/MeOH 90:10 as eluent to yield the Ni(II) complex **13** (3.92 g, 6.58 mmol, 66%) obtained as a red solid. Mp = 148-150 °C; Rf (EtOAc/MeOH, 95/5) = 0.3; ^{1}H NMR (300 MHz, $CDCl_3$): 8.23 (d, *J* = 8.7 Hz, 1H), 8.06 (d, *J* = 7.3 Hz, 2H), 7.55 (m, 3H), 7.36 (m, 3H), 7.19 (m, 2H), 6.92 (d, *J* = 7.4 Hz, 1H), 6.72–6.56 (m, 2H), 5.58 (d, *J* = 10.0 Hz, 1H), 4.37 – 4.22 (m, 2H), 3.66 (d, *J* = 8.9 Hz, 1H), 3.58 (d, *J* = 12.7 Hz, 1H), 3.51–3.34 (m, 3H), 2.88-2.83 (m, 1H), 2.63–2.44 (m, 1H), 2.08-2.03 (m, 2H); ^{13}C NMR (75 MHz, $CDCl_3$): 180.5, 178.2, 173.9, 143.2, 133.8, 133.6, 133.4, 133.3, 131.6, 130.8, 129.6, 129.1, 129.1, 127.9, 126.7, 126.3, 126.2, 123.8, 122.9, 120.9, 70.9, 68.8 (q, *J* = 30 Hz, $\underline{C}HCF_3$), 66.6, 63.7, 57.4, 30.8, 22.8; ^{19}F NMR (188 MHz, $CDCl_3$): -72.84 (*J* = 7.6 Hz); HRMS (TOF ESI, ion polarity positive): calcd for m/z calcd for $C_{29}H_{27}F_3N_3NiO_4$ $[M+H]^+$: 596.1307, found: 596.1309; m/z calcd for $C_{29}H_{26}F_3N_3NiO_4Na$ $[M+Na]^+$: 618.1127, found: 618.1145.

(2*S*,3*S*)-2-(*tert*-Butoxycarbonylamino)-4,4,4-trifluoro-3-hydroxybutyric acid (14). A solution of the Ni(II) complex **13** (3.6 g, 6 mmol) in MeOH (30 mL) was added to a diluted aqueous HCl solution (1 mL of conc. HCl and 2 mL H_2O for 1 g of the complex). The mixture was refluxed for 15–30 min and then cooled to room temperature. Most of the MeOH was then evaporated. EtOAc (50 mL) and water (50 mL) were added, and after separation, the organic phase was washed with 10% aqueous $NaHCO_3$ (50 mL), and brine, dried over Na_2SO_4, filtered and concentrated under vacuum. The crude residue obtained was the chiral auxiliary **11**, which could be reused. To the aqueous phase put aside were

added 2.0 equiv of NaSCN and 4.0 equiv of pyridine. The $Ni(Py)_4(SCN)_2$ precipitate immediately formed was filtered and then were added to the filtrate, dioxane (50 mL), Na_2CO_3 (1.27 g, 12 mmol) and Boc_2O (1.57 g, 7.2 mmol). The reaction mixture was stirred overnight at room temperature. After concentration under vacuum, the crude residue was taken up with CH_2Cl_2 (50 mL) and water (50 mL). The organic phase was separated and washed with 10% aqueous $NaHCO_3$ (50 mL) and brine, dried over Na_2SO_4, filtered and concentrated under vacuum. The crude residue obtained was purified by column chromatography on silica gel using ($MeOH/EtOAc/CH_3COOH$, 5/94/1) as eluent to afford **14** (368 mg, 1.35 mmol, 43%) as a colorless oil. ^1H NMR (300 MHz, $CDCl_3$): 7.77 (s, 1H), 6.32 (d, *J* = 9.5 Hz, 1H), 5.72 (d, *J* = 9.0 Hz, 1H), 4.67 (d, *J* = 9.1 Hz, 1H), 1.43 (s, 9H); ^{13}C NMR (75 MHz, CD_3OD): 172.7, 157.9, 125.9 (q, *J* = 280.5 Hz, $\underline{C}F_3$), 81.2, 70.4 (q, *J* = 30.8 Hz, $\underline{C}HCF_3$), 54.7, 28.8; ^{19}F NMR (188 MHz, $CDCl_3$): δ -76.14 (*J* = 7.6 Hz); HRMS (TOF ESI, ion polarity positive): calcd for $C_9H_{14}F_3NO_5Na$ $[M+Na]^+$: 296.0722, found: 296.0723; $[\alpha]_{589}^{17}$= - 4.9 (c, 1.02, MeOH) (to our knowledge not described in the literature).

(*S*)-2-((*S*)-2-(*tert*-Butoxycarbonylamino)-3-methylbutyrylamino)-4-methylpentanoic acid methyl ester (15).To a solution of Boc-L-Val-OH (4.0 g, 18.4 mmol) in anhydrous CH_2Cl_2 (20 mL) at −10 °C, were added *N*-methylmorpholine (4.4 mL, 22.1 mmol). After 30 min. of stirring, isobutylchloroformate (2.63 mL, 20.3 mmol) was added dropwise and the mixture was stirred at −10 °C for 45 min before the addition of H-Leu-OMe (3.7 g, 20.3 mmol). The reaction mixture was stirred at room temperature for an additional 24 h. After concentration under vacuum, the crude residue was taken up with EtOAc (40 mL). The organic phase was successively washed with 10% aqueous citric acid (50 mL), 10% aqueous K_2CO_3 (20 mL) and brine (20 mL), dried over Na_2SO_4, filtered and concentrated under vacuum. The oily crude residue obtained was purified by column chromatography on silica gel using cyclohexane/EtOAc 7:3 as eluent to afford the dipeptide **15** (5.2 g, 15.2 mmol, 83%) as a white solid. Mp = 142-144 °C; Rf (cyclohexane/EtOAc 7:3) = 0.35; ^1H NMR (300 MHz, $CDCl_3$): 6.25 (d, *J* = 6.9 Hz, 1H, NH), 5.05 (sl, 1H NH), 4.65 (m, 1H), 3.90 (dd, *J* = 9.0, 6.3 Hz, 1H), 3.74 (s, 3H), 2.11 (m, 1H), 1.66 (m, 3H), 1.44 (s, 9H), 1.00 - 0.93 (m, 12H); IR (neat): ν_{max}: 3335, 3263, 2960, 2052, 1757, 1686, 1650 cm^{-1}; MS (ESI, ion polarity positive): m/z 367.21 $[M+Na]^+$.

(*S*)-2-[(S)-2-((*S*)-3-Benzyloxy-2-*tert*-butoxycarbonylaminopropionylamino)-3-methylbutyrylamino]-4-methylpentanoic acid methyl ester (16a). Dipeptide **15** (0.5 g, 1.40 mmol) was deprotected according to general method A. The resulting salt was then coupled to Boc-L-Ser(Bzl)-OH (0.3 g, 1.53 mmol) according to general procedure B. After purification by column chromatography on silica gel

using using cyclohexane/EtOAc (70:30) then EtOAc (100%) as eluent, **16a** (0.35 g, 0.67 mmol, 48%) was obtained as a white solid. Mp = 94-96 °C; Rf (cyclohexane/EtOAc, 40:60) = 0.65; ^{1}H NMR (300 MHz, $CDCl_3$): 7.39-7.20 (m, 5H), 6.96 (d, *J* = 8.7 Hz, 1H), 6.61 (d, *J* = 7.4 Hz, 1H), 5.42 (m, 1H), 4.64-4.47 (m, 3H), 4.31 (m, 2H), 3.97-3.85 (m, 1H), 3.70 (s, 3H), 3.61 (dd, *J* = 9.2, 6.2 Hz, 1H), 2.17 (m, 1H), 1.67-1.49 (m, 3H), 1.44 (s, 9H), 0.97-0.79 (m, 12H); ^{13}C NMR (75 MHz, $CDCl_3$): 173.1, 170.6, 170.3, 155.6, 137.3, 128.4, 127.9, 127. 8, 80.4, 73.5, 69.8, 58.5, 52.2, 50.7, 41.2, 30.6, 28.2, 24.6, 22.7, 21.8, 19.1, 17.6; IR (neat): ν_{max}: 3435, 3308, 2961, 1749, 1643, 1524 cm^{-1}; MS (ESI, ion polarity positive): m/z 544[M+Na]$^+$; $C_{27}H_{43}N_3O_7$: cacld C% 62.17, H% 8.31, N% 8.06, found C% 61.84, H% 8.52, N% 7.92.

(*S*)-2-[(*S*)-2-((2*S*,3*R*)-3-Benzyloxy-2-(*tert*-butoxycarbonylamino)butyrylamino)-3-methyl-butyrylamino]-4-methylpentanoic methyl ester (16b). Dipeptide **15** (1.0 g, 3 mmol) was deprotected according to general method A. The resulting salt was then coupled to Boc-*L*-Thr(Bzl)-OH (950 mg, 3.07 mmol), according to general procedure B, to afford **16b** (1.23 g, 2.30 mmol, 83%) as a white solid after purification by column chromatography on silica gel using using cyclohexane/AcOEt (70:30) as eluent. Rf (cyclohexane/EtOAc, 3:7) = 0.8; ^{1}H NMR (300 MHz, $CDCl_3$) : 7.35 - 7.29 (m, 5H), 6.97 (d, *J* = 9.0 Hz, 1H), 6.36 (d, *J* = 8.2 Hz, 1H), 5.48 (d, *J* = 6.0 Hz, 1H), 4.68 - 4.53 (m, 3H), 4.30 - 4.20 (m, 3H), 3.73 (s, 3H), 2.19 (s, 1H), 1.66 - 1.50 (m, 3H), 1.48 (s, 9H), 1.22 (d, *J* = 6.3 Hz, 3H), 0.94 - 0.82 (m, 12H); ^{13}C NMR (75 MHz, $CDCl_3$): 173.0, 170.5, 170.1, 137.8, 128.4, 127.9, 127.8, 80.3, 74.7, 71.7, 58.7, 58.0, 52.2, 50.7, 41.2, 30.3, 28.2, 24.7, 22.7, 21.8, 19.2, 17.6, 15.3.

(*S*)-2-[(*S*)-2-((2*S*,3*R*)-3-Benzyloxy-2-(*tert*-butoxycarbonylamino)-4,4,4-trifluorobutyrylamino)-3-methyl-butyrylamino]-4-methylpentanoic methyl ester (16c). Dipeptide **15** (0.16 g, 0.41 mmol) was deprotected according to general method A. The resulting salt was then coupled to (2*S*,3*R*)-Boc-CF_3-Thr(Bzl)-OH (0.15 g, 0.45 mmol) to afford **16c** (0,22g, 0.27 mmol, 67%) as a white powder, after purification by column chromatography on silica gel using using first CH_2Cl_2 100% and then CH_2Cl_2/MeOH, (90:10) as eluent, and after crystallization from EtOAc/MeOH. Mp = 227-229 °C; Rf (cyclohexane/EtOAc, 40/60) = 0.8; ^{1}H NMR (400 MHz, $CDCl_3$): 7.42-7.32 (m, 5H), 6.82 (d, *J* = 8.3 Hz, 1H), 6.38 (d, *J* = 5.9 Hz, 1H), 5.24 (d, *J* = 7.2 Hz, 1H), 4.84-4.73 (m, 2H), 4.70-4.56 (m, 2H), 4.42 (m, 1H), 4.33 (dd, *J* = 8.4, 5.9 Hz, 1H), 3.77 (s, 3H), 2.32-2.19 (m, 1H), 1.74-1.51 (m, 3H), 1.48 (s, 9H), 1.04-0.88 (m, 12H); ^{13}C NMR (101 MHz, $CDCl_3$): 173.0, 170.2, 167.8, 167.78, 136.1, 128.5, 128.4, 128.3, 124.4 (q, *J* = 284 Hz, $\underline{C}F_3$), 81.1 ,75.9 (q, *J* = 27.3 Hz, $\underline{C}HCF_3$), 74.7, 58.6, 54.4, 52.3, 50.8, 41.2, 30.7, 28.2, 24.8, 22.7, 21.8, 18.9, 17.6; ^{19}F NMR (188MHz, CDl_3): -72.66 (d, *J* = 7.5 Hz); IR (neat): ν_{max}: 3600, 3554, 3465, 3326, 3271, 2981, 2409, 1750, 1693, 1641, 1532 cm^{-1}; MS (ESI, ion polarity positive): m/z 612

$[M+Na]^+$; MS (ESI, ion polarity negative): m/z 588 $[M-H]^-$; $C_{28}H_{42}F_3N_3O_7$: cacld C% 57.03, H% 7.18, N% 7.13, found C% 56.96), H% 7.17, N% 7.12.

(S)-2-[(S)-2-((2S,3S)-2-(*tert*-Butoxycarbonylamino)-4,4,4-trifluoro-3-hydroxybutyrylamino)-3-methylbutyrylamino]-4-methylpentanoic methyl ester (16d). Dipeptide **15** (1.03 g, 3 mmol) was deprotected according to general method A. The resulting salt was then coupled to (2*S*,3*S*)-Boc-CF_3-Thr-OH **14** (164 mg, 0.6 mmol) according to general procedure B, to afford **16d** (198 mg, 0.40 mmol, 73%) as a white solid, after purification by column chromatography on silica gel using using cyclohexane/EtOAc (2:2) as eluent; Rf (CH_2Cl_2/MeOH, 95:5) = 0.35; ^1H NMR (300 MHz, $CDCl_3$) : 7.41 (d, *J* = 8.9 Hz, 1H), 7.01 (d, *J* = 7.6 Hz, 1H), 5.61 (d, *J* = 8.4 Hz, 1H), 4.96 (s, 1H), 4.68–4.50 (m, 3H), 4.25 (t, *J* = 7.6 Hz, 1H), 3.71 (s, 3H), 2.14 (m, 1H), 1.67-1.55 (m, 3H), 1.45 (s, 9H), 0.94-0.90 (m, 12H); ^{13}C NMR (75 MHz, $CDCl_3$) : 172.0, 166.3, 142.4, 136.5, 126.7, 122.9, 62.6, 51.0, 50.1, 49.8, 37.6, 28.3, 24.9, 14.2; ^{19} F NMR (188 MHz, $CDCl_3$) : -77.19 (*J* = 7.6 Hz); IR (neat): ν_{max}: 3297, 2961, 2930, 1748, 1643, 1536, 1369, 1159, 1135 cm^{-1}; HRMS (TOF ESI, ion polarity positive): m/z calcd for $C_{21}H_{37}F_3N_3O_7$ $[M+H]^+$: 500.2584, found: 500.2584; m/z calcd for $C_{21}H_{40}F_3N_4O_7$ $[M+NH_4]^+$: 517.2865, found: 517.2842.

(*S*)-2-{(S)-2-[(*S*)-2-[(*S*)-3-Benzyloxy-2-((*S*)-2-(*tert*-butoxycarbonylamino)-3-methylbutyrylamino)-propionylamino]-3-methylbutyrylamino}-4-methylpentanoic methyl ester (17a). Tripeptide **16a** (0.31 g, 0.59 mmol) was deprotected according to general method A. The resulting salt was then coupled to Boc-Val-OH (0.14 g, 0.63 mmol), according to method B, to afford **17a** (0.23 g, 0.38 mmol, 64%) as a white solid after purification by column chromatography on silica gel using using cyclohexane/EtOAc (70:30) as eluent; Mp = 178-180 °C; Rf (cyclohexane/EtOAc, 20:20) = 0.80; ^1H NMR (300 MHz, $CDCl_3$) : 7.37-7.28 (m, 5H), 6.96 (m, 2H), 6.70 (d, 1H), 4.94 (d, 1H), 4.62-4.47 (m, 4H), 4.43-4.35 (m, 1H), 3.94 (m, 2H), 3.70 (s, 3H), 3.61 (dd, *J* = 9.4, 6.1 Hz, 1H), 2.34-2.15 (m, 2H), 1.64-1.59 (m, 3H), 1.40 (s, 9H), 1.02-0.86 (m, 18H); ^{13}C NMR (75 MHz, $CDCl_3$): 173.1, 171.7, 167.3, 136.3, 128.6, 128.3, 128.00, 73.8, 69.00, 59.9, 52.319, 51.1, 40.3, 30.7, 24.8, 22.5, 21.5, 18.7, 18.00; IR (neat): ν_{max}: 3297, 2961, 2930, 1748, 1643, 1536, 1369, 1159, 1135 cm^{-1}; ESI, ion polarity positive): m/z 643 $[M+Na]^+$.

(*S*)-2-{(*S*)-2-[(2*S*,3*R*)-3-Benzyloxy-2-((*S*)-2-(*tert*-butoxycarbonylamino)-3-methylbutyrylamino)-butyrylamino]-3-methylbutyrylamino}-4-methylpentanoic methyl ester (17b). Tripeptide **16b** (600 mg, 1.09 mmol) was deprotected according to general method A. The resulting salt was then coupled

to Boc-Val-OH (260 mg, 1.20 mmol), according to method B, to afford **17b** (420 mg, 0.66 mmol, 61%) as a white solid after purification by column chromatography on silica gel using CH_2Cl_2/MeOH (97:3) as eluent; Rf (CH_2Cl_2/MeOH, 95:5) = 0.6); ^{1}H NMR (300 MHz, $CDCl_3$): 7.35 - 7.28 (m, 5H), 7.11-7.05 (m, 2H), 6.71 (d, *J* = 7.8 Hz, 1H), 5.00 (d, *J* = 5.7 Hz, 1H), 4.69 - 4.54 (m, 3H), 4.40 (m, 1H), 4.23 (m, 1H), 4.03 (m, 1H), 3.72 (s, 3H), 2.23 (s, 2H), 1.61 (m, 4H), 1.41 (s, 9H), 1.19 (d, *J* = 6.3 Hz, 3H), 1.02-0.86 (m, 18H); ^{13}C NMR (75 MHz, $CDCl_3$): 173.1, 172.1, 170.8, 169.6, 156.1, 137.7, 128.4, 127.8, 127.8, 80.4, 74.1, 71.7, 60.3, 58.8, 57.0, 52.1, 50.7, 40.9, 30.7, 30.1, 28.2, 24.7, 22.8, 21.7, 19.4, 19.1, 17.6, 17.5, 15.6; IR (neat): ν_{max}: 3373, 2961, 2873, 1752, 1689, 1637, 1521, 1299, 1244, 1159 cm^{-1} HRMS (TOF ESI, ion polarity positive): m/z calcd for $C_{33}H_{54}N_4O_8$ Na[M+Na]$^+$: 657.3839, found: 657.3829

(*S*)-2-{(*S*)-2-[(2*S*,3*R*)-3-Benzyloxy-2-((*S*)-2-(*tert*-butoxycarbonylamino)-3-methylbutyrylamino)-4,4,4-trifluorobutyrylamino]-3-methylbutyrylamino}-4-methylpentanoic methyl ester (17c). Tripeptide **16c** (0.12 g, 0.20 mmol) was deprotected according to general method A. The resulting salt was then coupled to Boc-Val-OH (0.05 g, 0.22 mmol), according to method B, to afford **17c** (0.12 g, 0.17 mmol, 87%) as a white powder from crystallization in EtOAc/CH_2Cl_2; Mp = 262-264 °C; Rf (EtOAc/MeOH, 90:10) = 0.9; ^{1}H NMR (400 MHz, DMSO-*d$_6$*): 8.32 (m, 2H), 8.17 (d, *J* = 9.2 Hz, 1H), 7.36-7.22 (m, 5H), 6.66 (d, *J* = 9.0 Hz, 1H), 5.01 (dd, *J* = 8.9 Hz, 1H), 4.68 (d, *J* = 10.9 Hz, 1H), 4.58 (d, *J* = 10.9 Hz, 1H), 4.31-4.19 (m, 3H), 3.81 (dd, *J* = 18.8, 10.9 Hz, 1H), 3.58 (s, 3H), 2.02-1.83 (m, 2H), 1.65-1.44 (m, 3H), 1.37 (s, 9H), 0.90-0.70 (m, 18H); ^{13}C NMR (101 MHz, DMSO-*d$_6$*): 172.7, 170.8, 170.6, 167.5, 155.2, 136.8, 128.04, 128.0, 127.8, 125.00, 78.0, 75.6(m, <u>C</u>HCF_3), 74.5, 59.7, 57.7, 51.7, 51.0, 50.3, 39.5, 30.9, 30.4, 28.1, 24.1, 22.6, 21.2, 19.1, 18.9, 18.1, 18.0 ; IR (neat): ν_{max}: 3580, 3282, 2960, 1672, 1639, 1529, 1255 cm^{-1}; ESI (ion polarity positive): m/z 711.6 [M+Na]$^+$.

(*S*)-2-{(S)-2-[(2*S*,3*S*)-2-(*tert*-bButoxycarbonylamino)-3-methylbutyrylamino)-4,4,4-trifluoro-3-hydroxybutyrylamino]-3-methylbutyrylamino}-4-methylpentanoic methyl ester (17d). Tripeptide **16d** (113 mg, 0.22 mmol) was deprotected according to general method A. The resulting salt was then coupled to Boc-Val-OH (44 mg, 0.20 mmol), according to method C, to afford **17d** (86 mg, 0.14 mmol, 72%) as a white solid after purification by column chromatography on silica gel using CH_2Cl_2/MeOH (98/2) as eluent; Rf (CH_2Cl_2/MeOH, 95:5) = 0.20; ^{1}H NMR (300 MHz, DMSO-*d$_6$*): 8.31 (d, *J* = 7.2 Hz, 1H), 8.16 (d, *J* = 9.0 Hz, 1H), 7.48 (d, *J* = 8.8 Hz, 1H), 6.84 (d, *J* = 8.7 Hz, 2H), 4.86 (s, 1H), 4.73 (d, *J* = 8.7 Hz, 1H), 4.52 – 4.40 (m, 1H), 4.29-4.21 (m, 2H), 4.10-4.05 (m, 1H), 3.91 (t, *J* = 8.1 Hz, 1H), 3.59 (s, 3H), 1.98-1.95 (m, 2H), 1.64 – 1.44 (m, 3H), 1.37 (s, 9H), 0.91 – 0.77 (m, 18H); ^{13}C NMR (75 MHz, DMSO-*d$_6$*): 172.7, 171.7, 170.4, 167.8, 155.5, 126.6, 78.1, 68.3, 67.9, 59.8, 57.2, 51.8, 51.7,

50.3, 31.1, 30.1, 28.1, 24.2, 22.7, 21.2, 19.2, 19.0, 18.0, 17.7; ^{19}F NMR (188 MHz, CD_3OD): -80.44 (J = 7.6 Hz); IR (neat): v_{max}: 3279, 2961, 2930, 1753, 1639, 1547, 1368, 1176, 1160 cm^{-1}; HRMS (TOF ESI, ion polarity positive): m/ calcd for $C_{26}H_{46}F_3N_4O_8$ $[M+H]^+$: 599.3268, found: 599.3286; m/z calcd for $C_{26}H_{49}F_3N_5O_8$ $[M+NH_4]^+$: 616.3533, found: 616.3528.

(*S*)-2-((*S*)-2-{(*S*)-3-Benzyloxy-2-[(*S*)-2-((*S*)-2-(*tert*-butoxycarbonylamino)propionylamino)-3-methylbutyrylamino]propionylamino}-3-methylbutyrylamino)-4-methylpentanoic acid methyl ester (18a). Tetrapeptide **17a** (0.19 g, 0.30 mmol) was deprotected according to general method A. The resulting salt was then coupled to Boc-Ala-OH (0.06 g, 0.33 mmol), according to method B, to afford **18a** (0.13 g, 0.19 mmol, 63%) as a white powder after purification by column chromatography on silica gel using cyclohexane/EtOAc (70:30) as eluent; Mp = 237-239 °C; Rf (cyclohexane/EtOAc, 40:60) = 0.3; ^{1}H NMR (300 MHz, $CDCl_3$): 8.75 (m, 1H), 8.43 (m, 1H), 8.03 (m, 2H), 7.17 (m, 5H), 6.15 (m, 1H), 5.34 (m, 1H), 4.98 (m, 1H), 4.77-4.54 (m, 3H), 4.40-4.19 (m, 2H), 3.69 (s, 3H), 3.46 (m, 2H), 2.06 (m, 1H), 1.93 (m, 1H), 1.70-1.47 (m, 3H), 1.42 (s, 9H), 1.29 (d, J = 12.8, 5.9 Hz, 3H), 1.02-0.68 (m, 18H). ^{13}C NMR (75 MHz, $CDCl_3$): 173.2, 173.1, 171.4, 171.3, 170.1, 155.6, 138.0, 128.2, 127.2, , 79.1, 72.7, 70.3, 58.6, 58.1, 52.1, 51.9, 50.7, 40.5, 32.5, 31.5, 28.4, 24.9, 22.5, 22.0, 18.8, 18.7; IR (neat): v_{max}: 3479, 3447, 3264, 2970,1715, 1632 cm^{-1}; (ESI, ion polarity positive): m/z 692 $[M+H]^+$.

(*S*)-2-((*S*)-2-{(2*S*,3*R*)-3-Benzyloxy-2-[(*S*)-2-((*S*)-2-(*tert*-butoxycarbonylamino)propionylamino)-3-methylbutyrylamino]butyrylamino}-3-methylbutyrylamino)-4-methylpentanoic acid methyl ester (18b). Tetrapeptide **17b** (380 mg, 0.59 mmol) was deprotected according to general method A. The resulting salt was then coupled to Boc-Ala-OH (122 mg, 0.64 mmol), according to method B, to afford **18b** (115 mg, 0.163 mmol, 28%) as a yellowish powder which being very poorly soluble could not be purified. Rf (cyclohexane/EtOAc, 70:30) = 0.85; ^{1}H NMR (300 MHz, DMSO-d_6): 8.28 (d, J = 7.5 Hz, 1H): 8.01 (d, J = 8.7 Hz, 1H), 7.65 (d, J = 9.3 Hz, 2H), 7.31 - 7.27 (m, 5H), 7.00 (d, J = 7.3 Hz, 1H), 4.56-4.23 (m, 5H), 3.95 (m, 2H), 3.57 (s, 3H), 1.90 (m, 2H), 1.47 (m, 2H), 1.35 (s, 9H), 1.21 (m, 2H), 1.15 (d, J = 7.2 Hz, 3H), 1.05 (d, J = 5.7 Hz, 3H), 0.88-0.74 (m, 18H); ^{13}C NMR (75 MHz, DMSO-d_6): 173.5, 172.7, 171.2, 170.8, 169.2, 155.0, 138.5, 128.0, 127.6, 127.2, 78.0, 75.0, 70.5, 57.2, 56.4, 51.7, 50.1, 49.7, 30.8, 28.1, 24.1, 22.6, 21.1, 19.1, 18.9, 17.9, 16.9, 16.2; HRMS (TOF ESI, ion polarity positive): m/z calcd for m/z calcd for $C_{36}H_{59}N_5O_9Na$ $[M+Na]^+$: 728.4210, found: 728.4214; m/z calcd for $C_{36}H_{60}N_5O_9$ $[M+H]^+$: 706.4391, found: 706.4376.

(*S*)-2-((*S*)-2-{(2*S*,3*R*)-3-Benzyloxy-2-[(*S*)-2-((*S*)-2-(*tert*-butoxycarbonylamino)propionylamino)-3-methylbutyrylamino]-4,4,4-trifluorobutyrylamino}-3-methylbutyrylamino)-4-methylpentanoic acid methyl ester (18c). Tetrapeptide **17c** (0.1 g, 0.14 mmol) was deprotected according to general method A. The resulting salt was then coupled to Boc-Ala-OH (0.03 g, 0.16 mmol), according to method B, to afford **18c** (0.09 g, 0.12 mmol, 83%) as a white solid. A slightly modified procedure was followed compared to method B: water was added at the end of reaction and the crude precipitate obtained was washed successively with ether, CH_2Cl_2, acetone, EtOAc and MeOH; Mp > 260 °C; Rf (cyclohexane) = 0.65; ^{1}H NMR (300 MHz, DMSO-d_6): 8.43-8.25 (m, 3H), 7.53 (d, J = 9.0 Hz, 1H), 7.32-7.24 (m, 5H), 7.05 (d, J = 7.7 Hz , 1H), 4.98 (t, J = 9.1 Hz), 4.65-4.56 (m, 2H), 4.25 (m, 3H), 4.05-3.90 (m, 1H), 3.58 (s, 3H), 1.97 (m, 2H), 1.64-1.42 (m, 3H), 1.37 (s, 9H), 1.14 (d, J = 7.1 Hz, 3H), 0.80 (m, 18H); ^{13}C NMR (101MHz, DMSO-d_6): 172.6, 172.4, 170.7, 170.2, 167.6, 155.1, 136.8, 128.1, 124.8 (m, $\underline{C}F_3$),, 78.1, 74.7, 57.7, 56.9, 51.6, 51.2, 50.3, 49.8, , 31.1, 30.9, 28.1, 24.1, 22.6, 21.2, 18.9, 18.9, 18.1, 17.8, 17.7 ; RMN ^{19}F (188 MHz, $CDCl_3$): 72.82 (d, J = 5.6 Hz); IR (neat): ν_{max}: 3573, 3273, 2959, 2900, 1720, 1636, 1549, 1453 cm^{-1}; (ESI, ion polarity negative): 758[M-H]$^-$; $C_{36}H_{56}F_3N_5O_9$•2 C_3H_6O: cacld C% 57.59, H% 7.84, N% 8.00, found C% 57.12, H% 7.43, N% 7.65

^{19}F and ^{1}H NMR spectra of 7a

NMR spectra of diasteroisomer **7a** showed the presence of two rotamers : 58/42 as confirmed by ^{19}F NMR experiment ^{19}F NMR (188MHz, $CDCl_3$): -74.45 (d, J = 7.5 Hz), -74.68 (d, J = 7.5 Hz). These rotamers disappeared by heating the fluor device at different temperatures (see C, D, E) or by changing the NMR solvent (A and B).

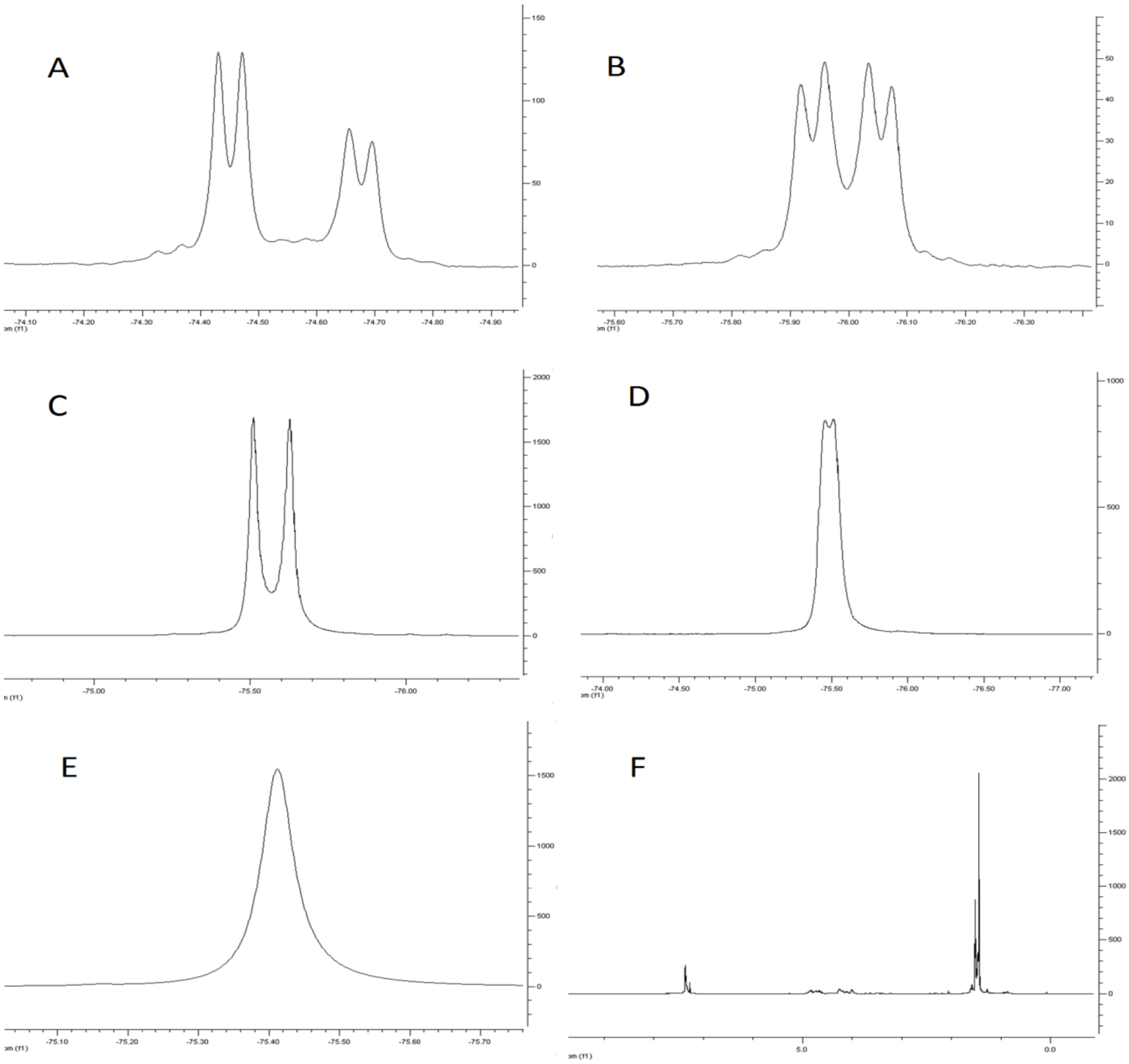

Figure. S1 : NMR spectra of ^{1}H-^{19}F coupling of **7a**, **A** : in $CDCl_3$, **B** : in CD_3OD. NMR spectrums of ^{19}F decoupling from ^{1}H of **7a** in CD_3OD at 300 K (**C**); at 313 K (**D**); and at 323 K (**E**). ^{1}H NMR spectrum of **7a** in $CDCl_3$ (**F**).

NMR data for the conformational studies of pentapeptides 1a–4a and 1b–4b

NMR study of pentapeptide **1a** in CD_3OH:

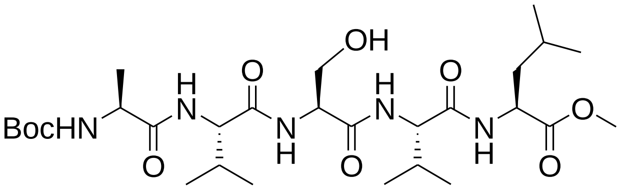

1a

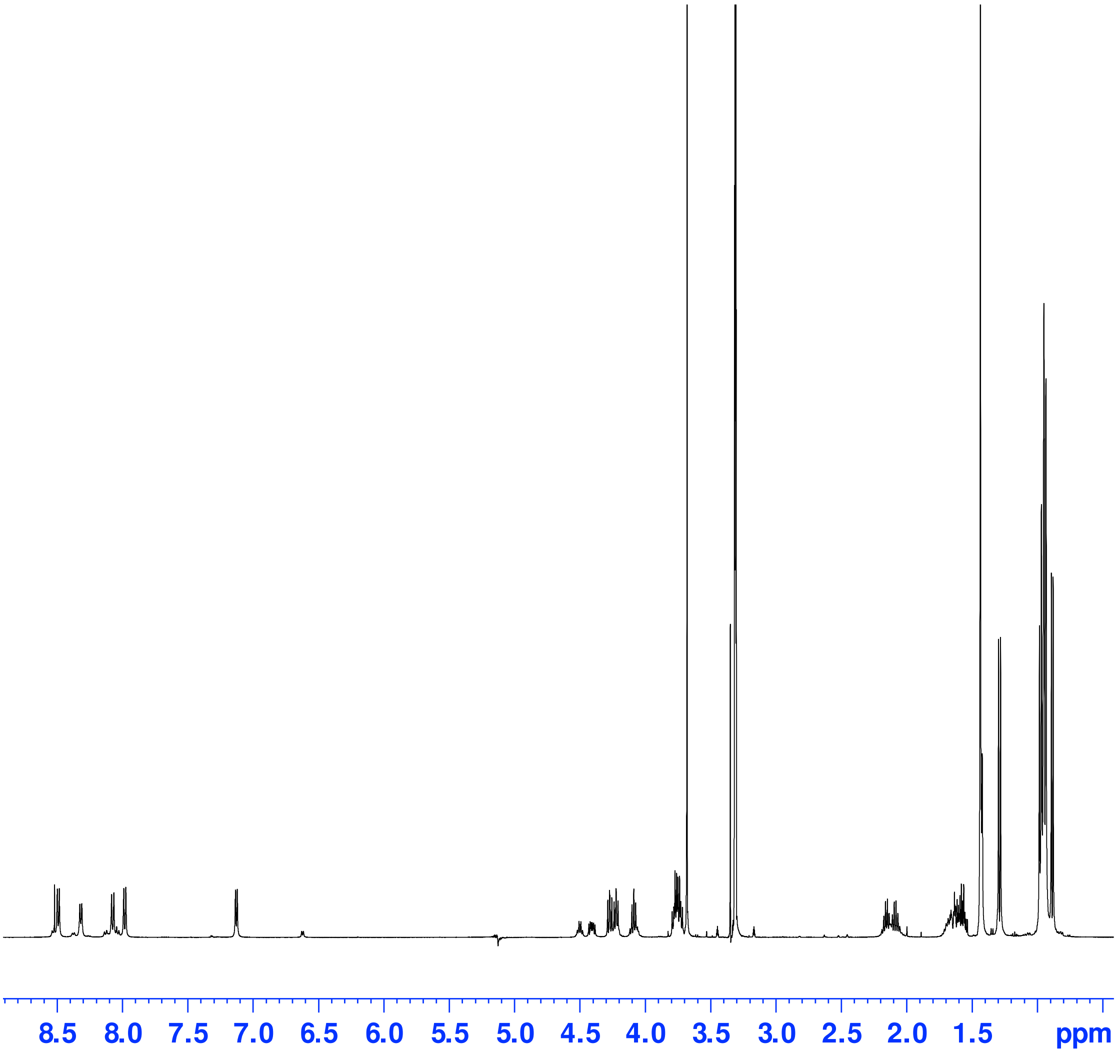

Figure S2 1D ^{1}H NMR spectrum of pentapeptide **1a** in CD_3OH (271 K)

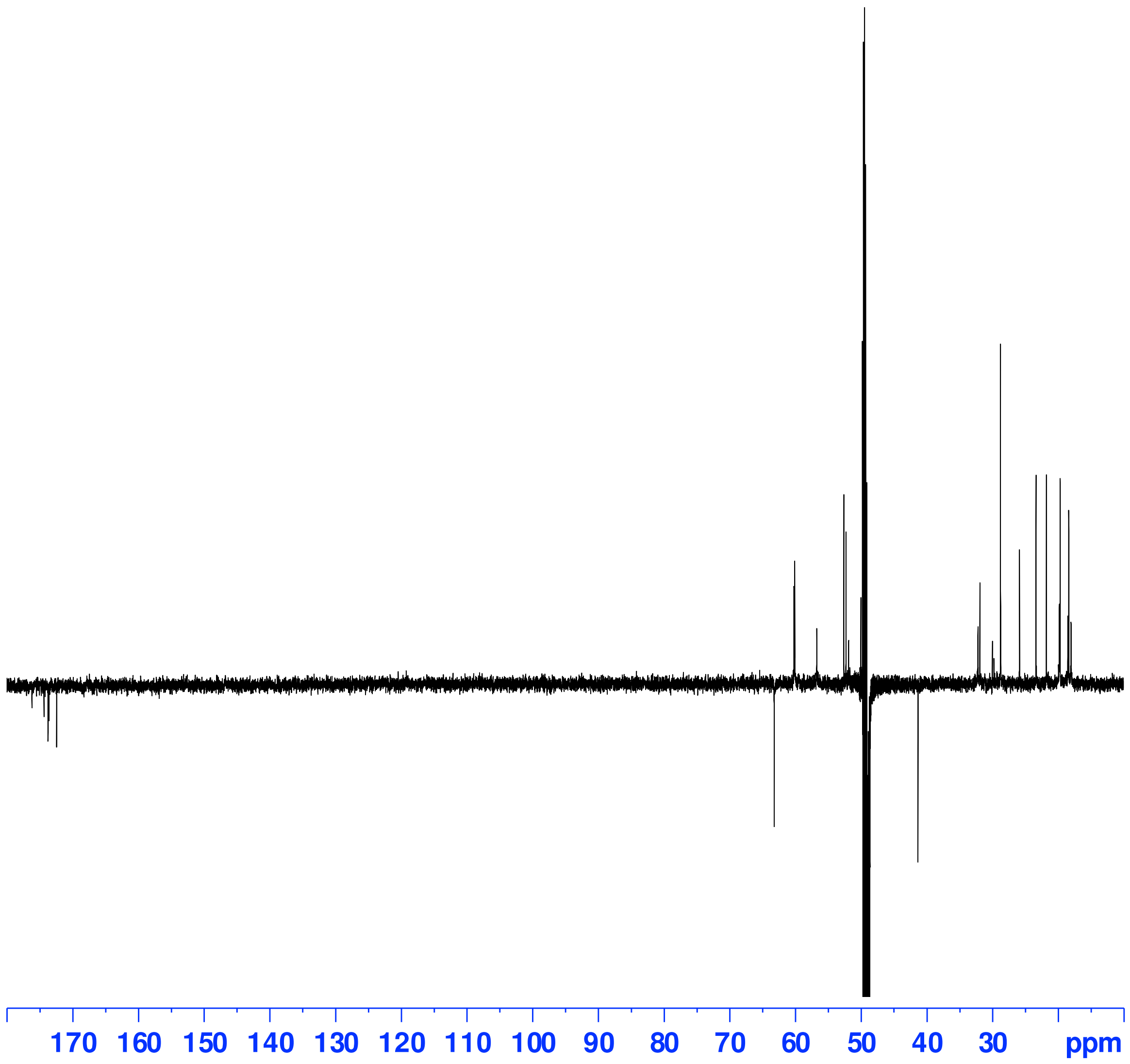

Figure S3 1D ^{13}C DEPTQ NMR spectrum of pentapeptide **1a** in CD_3OH (298 K)

Table S1 ^{1}H and ^{13}C NMR chemical shifts of pentapeptide **1a** in CD_3OH (298 K) [a]

Residue	δ NH (ppm)	δ H$^{\alpha}$ (ppm)	δ H$^{\beta}$ (ppm)	δ H$^{\gamma}$ & H$^{\delta}$ (ppm)	δ CO (ppm)	δ C$^{\alpha}$ (ppm)	δ C$^{\beta}$ (ppm)	δ C$^{\gamma}$ & C$^{\delta}$ (ppm)
Ala[1 b]	6.94	4.09	1.30		176.2	51.9	18.0	
Val[2]	7.82	4.21	2.10	0.97, 0.95	173.6	60.2	32.2	19.8, 18.5
Ser[3]	8.12	4.48	3.78, 3.74		172.4	56.7	63.2	
Val[4]	7.87	4.27	2.15	0.97, 0.95	173.7	60.1	31.9	19.7, 18.3
Leu[5]	8.31	4.43	1.60, 1.60	1.68, 0.94, 0.89	174.4	52.3	41.3	25.8, 23.3, 21.8

[a] The reported assignments correspond to the major Boc *anti* rotamer.

[b] Boc assignment: ^{1}H 1.44 ppm; ^{13}C 28.8, 80.8, 157.7 ppm. [c] OMe: ^{1}H 3.68 ppm; ^{13}C 52.6 ppm.

NMR study of pentapeptide **1b** in CD_3OH:

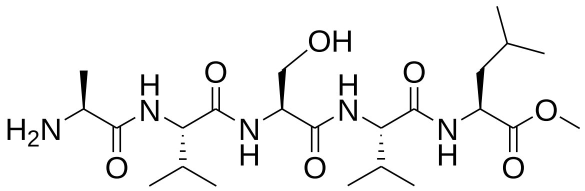

1b

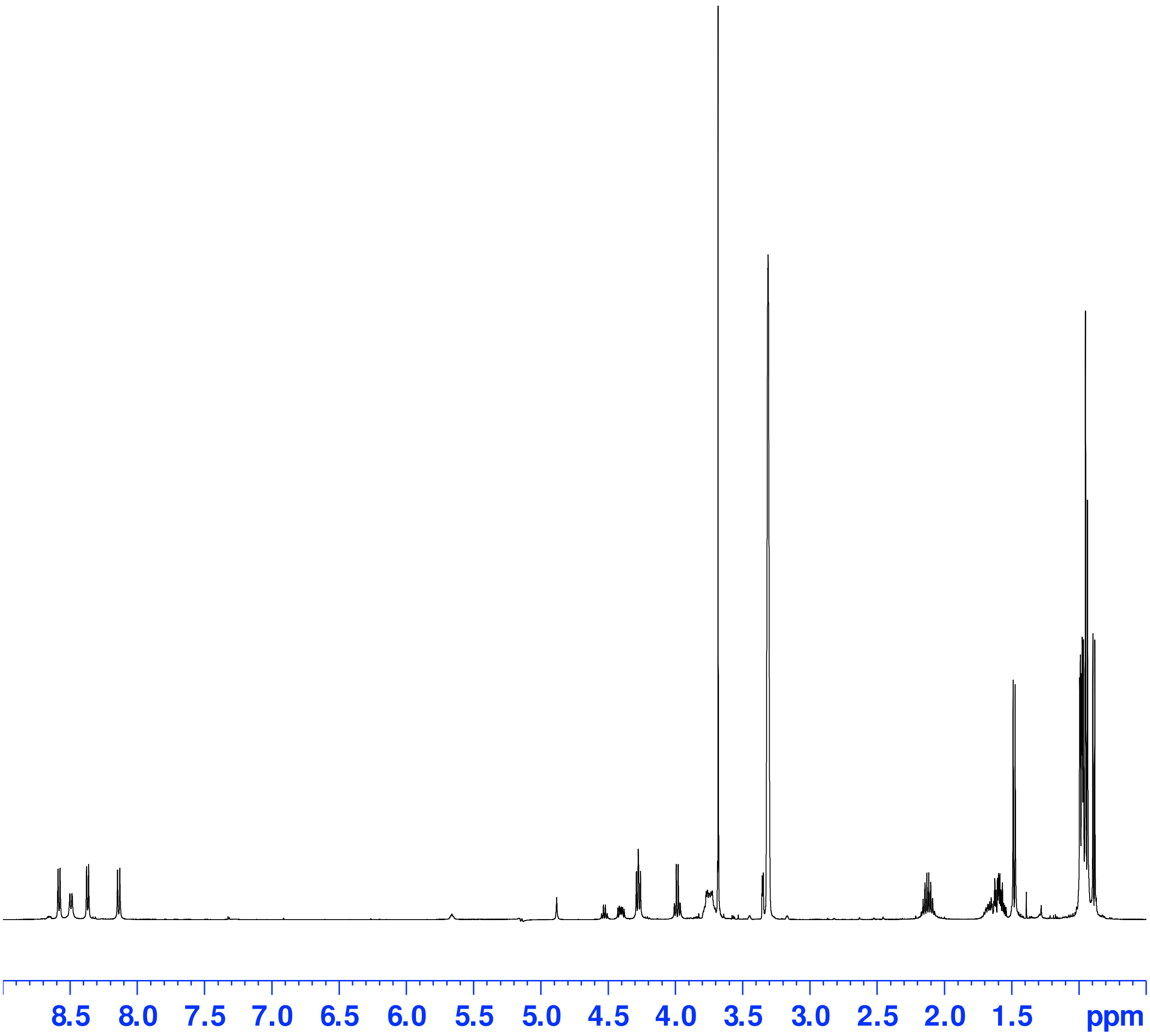

Figure S4 1D ^{1}H NMR spectrum of pentapeptide **1b** in CD_3OH (271 K)

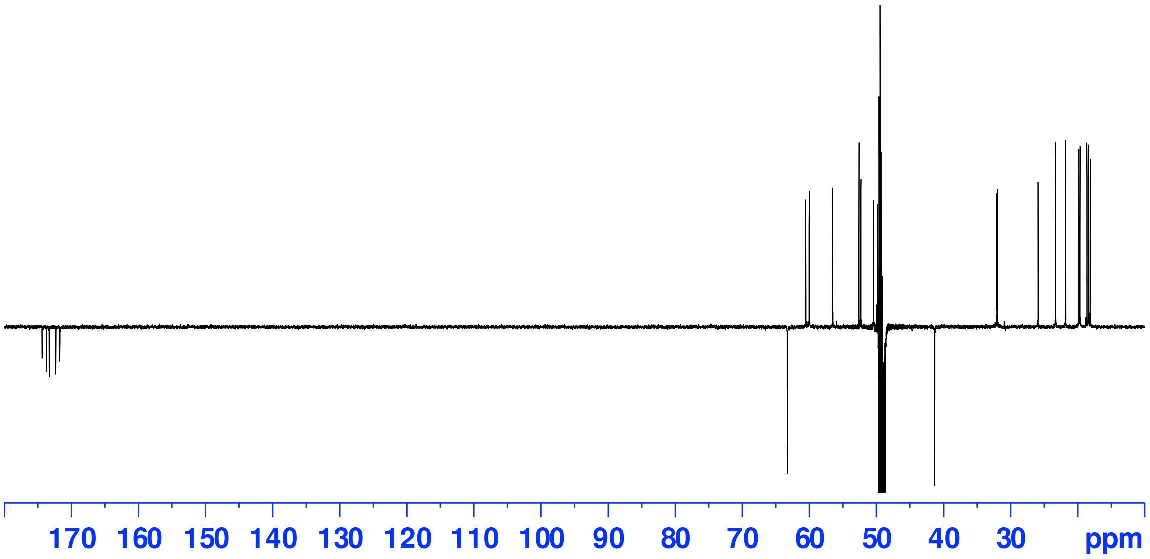

Figure S5 1D ^{13}C DEPTQ NMR spectrum of pentapeptide **1b** in CD_3OH (298 K)

Table S2 ^{1}H and ^{13}C NMR chemical shifts of pentapeptide **1b** in CD_3OH (298 K)

Residue	δ NH (ppm)	δ H$^{\alpha}$ (ppm)	δ H$^{\beta}$ (ppm)	δ H$^{\gamma}$ & H$^{\delta}$ (ppm)	δ CO (ppm)	δ C$^{\alpha}$ (ppm)	δ C$^{\beta}$ (ppm)	δ C$^{\gamma}$ & C$^{\delta}$ (ppm)
Ala[1]	-	3.97	1.51	-	171.7	50.4	18.1	
Val[2]	8.33 [a]	4.26	2.12	0.99, 0.97	173.3	60.5	32.0	19.8, 18.6
Ser[3]	8.15	4.51	3.78, 3.74	-	172.3	56.5	63.3	
Val[4]	7.91	4.27	2.13	0.98, 0.94	173.7	60.0	32.0	19.6, 18.3
Leu[5] [b]	8.39	4.42	1.61	1.67, 0.94, 0.89	174.4	52.3	41.3	25.9, 23.3, 21.8

[a] broad signal ; [b] OMe assignment: ^{1}H 3.68 ppm; ^{13}C 52.6 ppm.

NMR study of pentapeptide (2*S*,3*R*)-**2a** in CD_3OH:

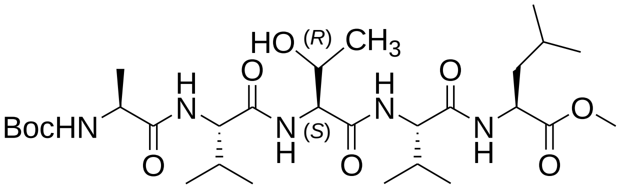

2a

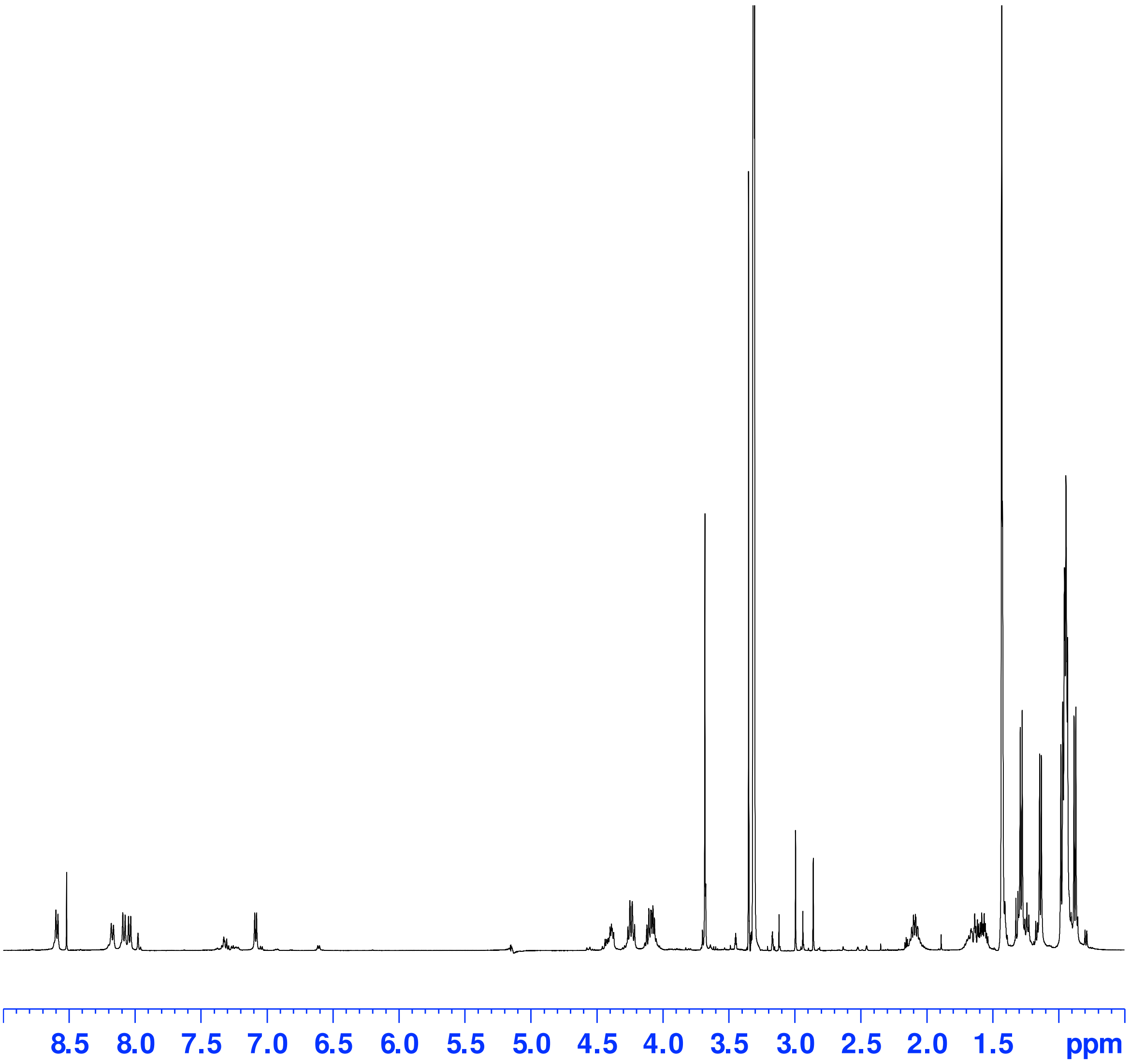

Figure S6 1D ^1H NMR spectrum of pentapeptide **2a** in CD_3OH (271 K)

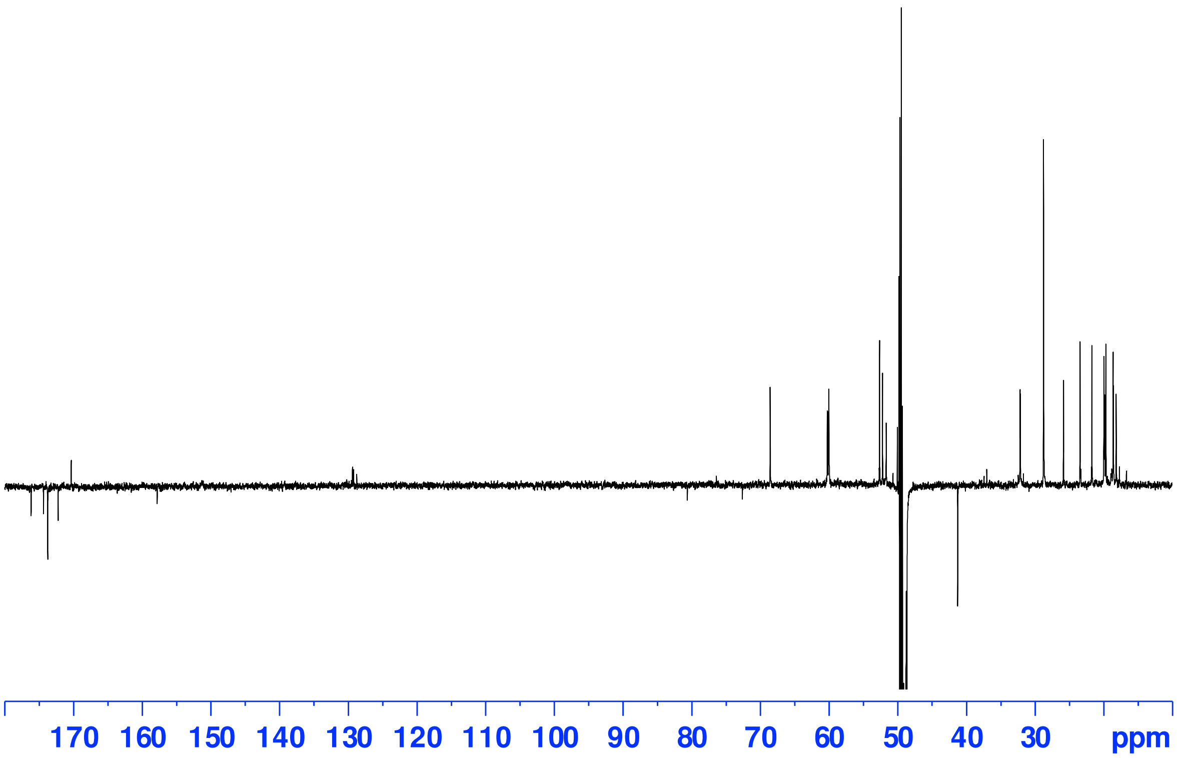

Figure S7 1D ^{13}C DEPTQ NMR spectrum of pentapeptide **2a** in CD_3OH (288 K)

Table S3 ^{1}H and ^{13}C NMR chemical shifts of pentapeptide **2a** in CD_3OH (298 K) [a]

Residue	δ NH (ppm)	δ H$^{\alpha}$ (ppm)	δ H$^{\beta}$ (ppm)	δ H$^{\gamma}$ & H$^{\delta}$ (ppm)	δ CO (ppm)	δ C$^{\alpha}$ (ppm)	δ C$^{\beta}$ (ppm)	δ C$^{\gamma}$ & C$^{\delta}$ (ppm)
Ala[1] [b]	7.09	4.10	1.29		176.1	51.6	18.1	
Val[2]	8.04	4.25	2.07	0.96, 0.95	173.7	60.2	32.1	19.8, 18.6
Thr[3]	8.17	4.39	4.07	1.13	172.2	60.0	68.5	19.9
Val[4]	8.08	4.23	2.08	0.98, 0.96	173.7	60.0	32.1	19.7, 18.6
Leu[5] [c]	8.59	4.40	1.63, 1.57	1.67, 0.94, 0.88	174.3	52.2	41.2	25.8, 23.4, 21.7

[a] The reported assignments correspond to the major Boc *anti* rotamer.

[b] Boc assignment: ^{1}H 1.43 ppm; ^{13}C 28.7, 80.6, 157.8 ppm. [c] OMe: ^{1}H 3.68 ppm; ^{13}C 52.6 ppm.

NMR study of pentapeptide (2*S*,3*R*)-**2b** in CD_3OH:

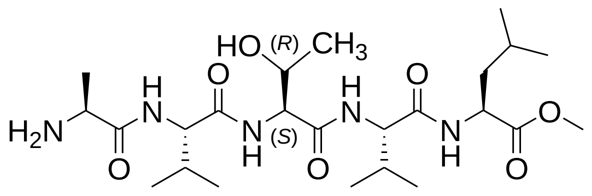

2b

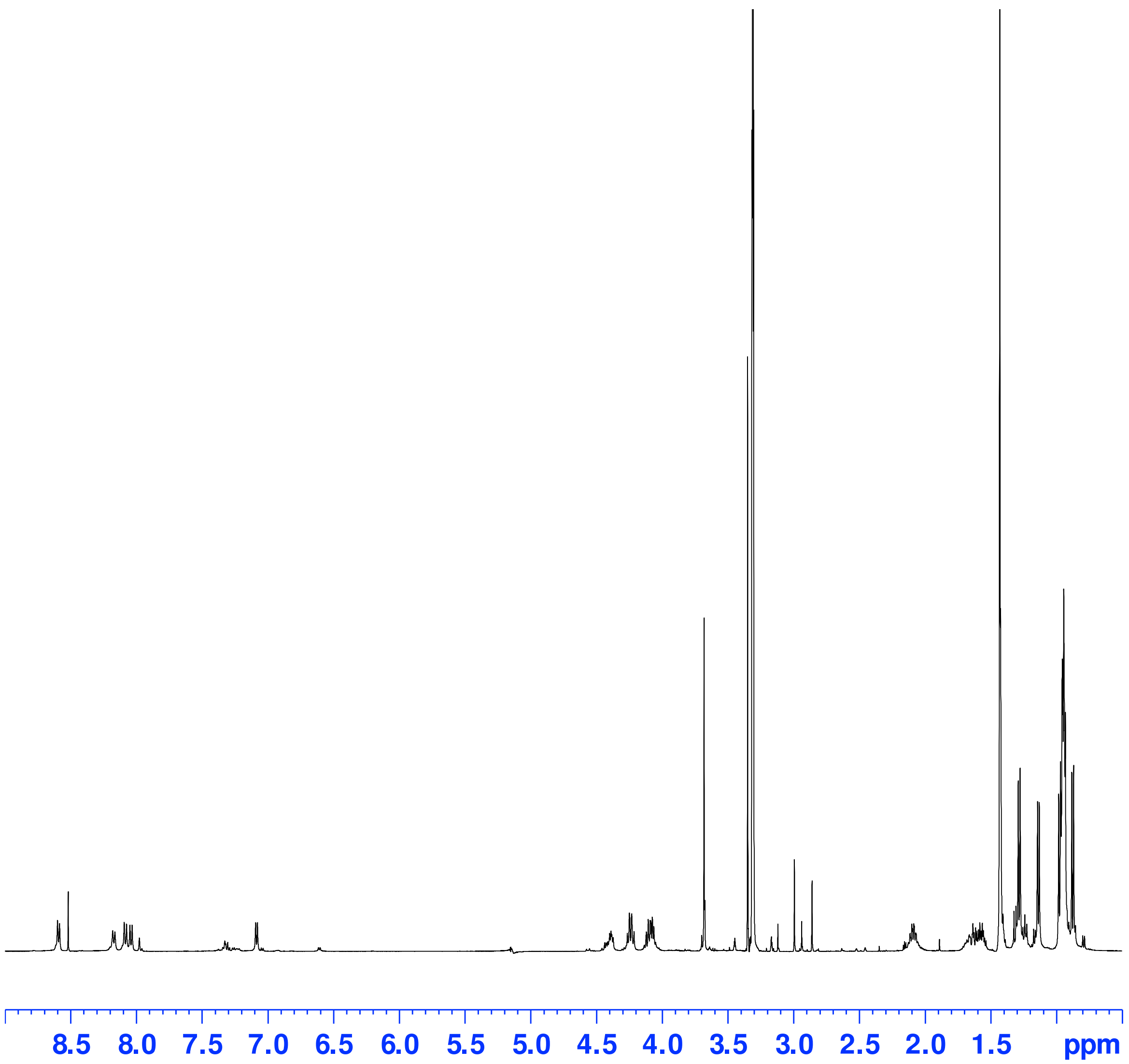

Figure S8 1D ^1H NMR spectrum of pentapeptide **2b** in CD_3OH (271 K)

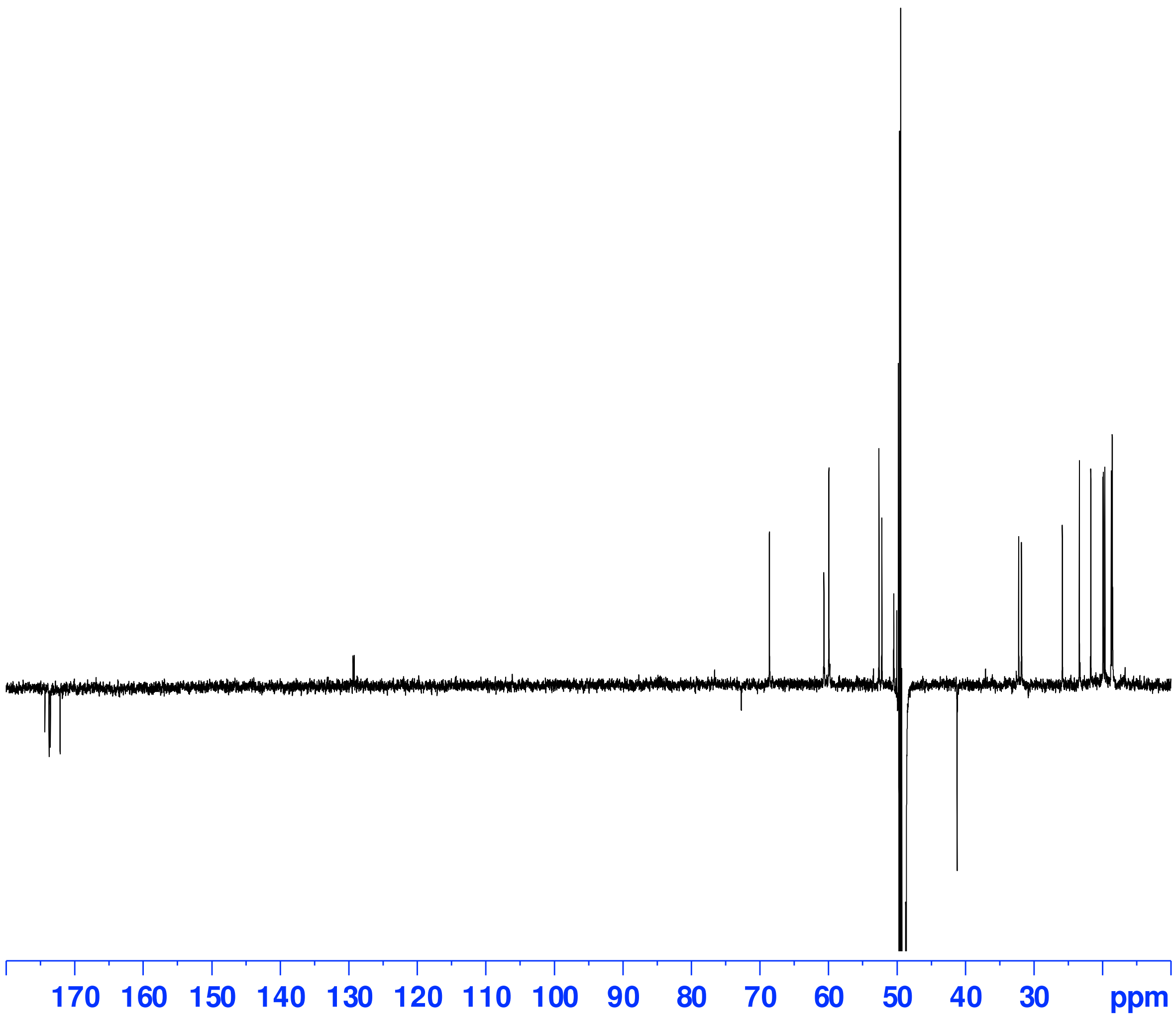

Figure S9 1D ^{13}C DEPTQ NMR spectrum of pentapeptide **2b** in CD_3OH (288 K)

Table S4 ^{1}H and ^{13}C NMR chemical shifts of pentapeptide **2b** in CD_3OH (288 K)

Residue	δ NH (ppm)	δ H$^{\alpha}$ (ppm)	δ H$^{\beta}$ (ppm)	δ H$^{\gamma}$ & H$^{\delta}$ (ppm)	δ CO (ppm)	δ C$^{\alpha}$ (ppm)	δ C$^{\beta}$ (ppm)	δ C$^{\gamma}$ & C$^{\delta}$ (ppm)
Ala[1]	-	3.91	1.45		171.4	50.4	18.5	
Val[2]	8.42	4.26	2.12	0.98, 0.95	173.5	60.6	31.8	19.8, 18.5
Thr[3]	8.08	4.40	4.10	1.14	172.1	59.9	68.6	19.9
Val[4]	7.99	4.24	2.10	0.98, 0.97	173.7	59.9	32.2	19.6, 18.7
Leu[5] [a]	8.53	4.41	1.60	1.67, 0.94, 0.88	174.3	52.2	41.2	25.8, 23.4, 21.7

[a] OMe assignment: ^{1}H 3.68 ppm; ^{13}C 52.6 ppm.

NMR study of pentapeptide (2*S*,3*R*)-**3a** in CD_3OH:

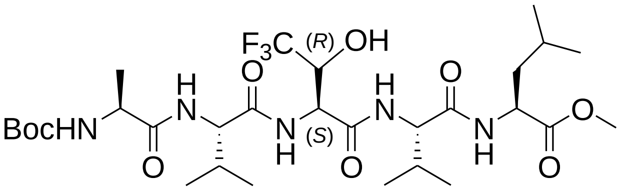

3a

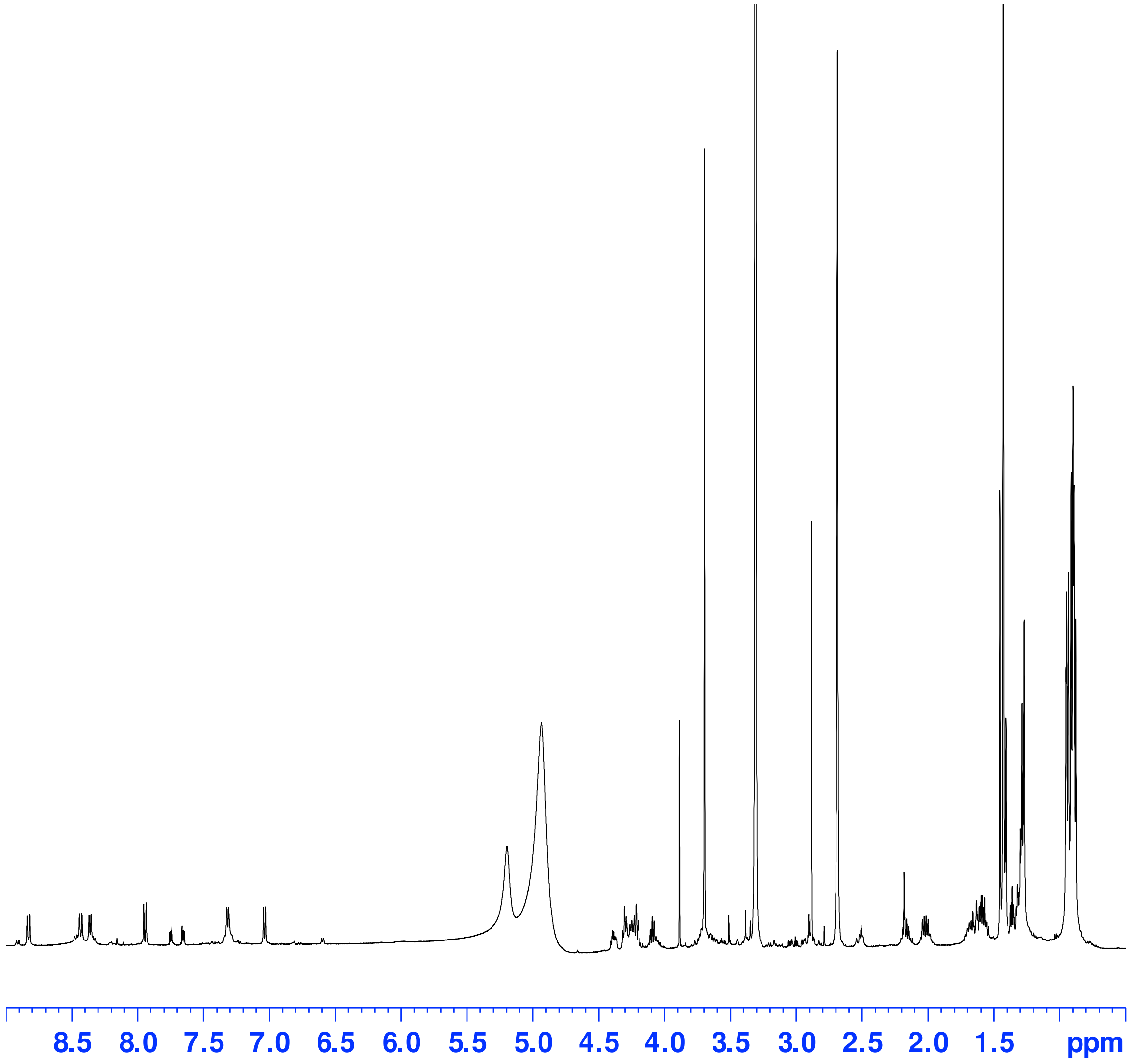

Figure S10 1D ^{1}H NMR spectrum of pentapeptide **3a** in CD_3OH (271 K)

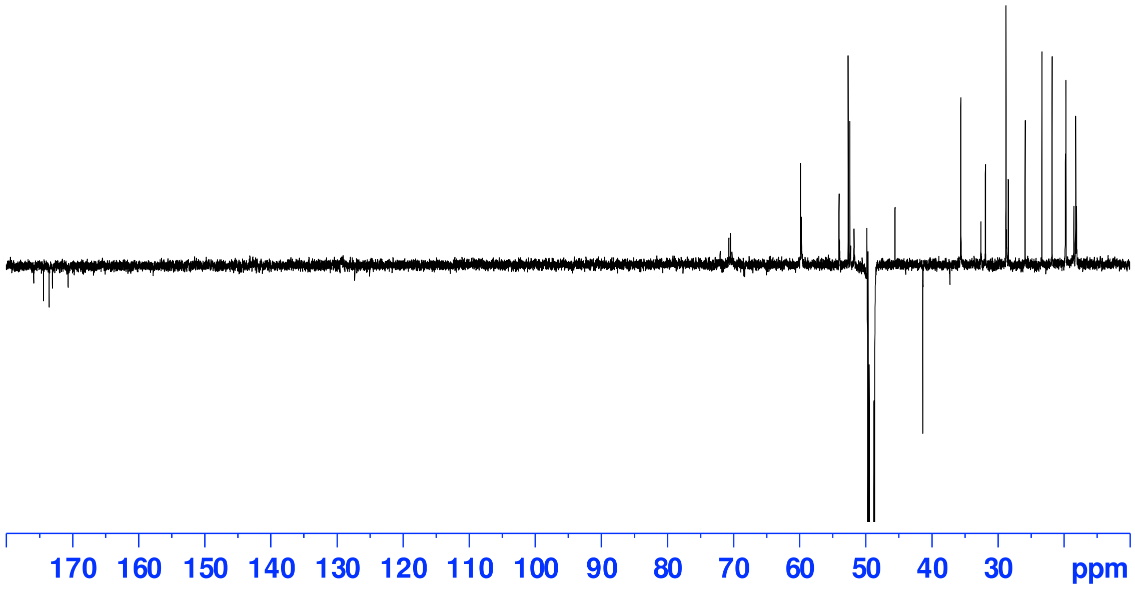

Figure S11 1D ^{13}C DEPTQ NMR spectrum of pentapeptide **3a** in CD_3OH (298 K)

Table S5 ^{1}H and ^{13}C NMR chemical shifts of pentapeptide **3a** in CD_3OH (298 K) [a]

Residue	δ NH (ppm)	δ H$^{\alpha}$ (ppm)	δ H$^{\beta}$ (ppm)	δ H$^{\gamma}$ & H$^{\delta}$ (ppm)	δ CO (ppm)	δ C$^{\alpha}$ (ppm)	δ C$^{\beta}$ (ppm)	δ C$^{\gamma}$ & C$^{\delta}$ (ppm)
Ala[1 b]	6.89	4.10	1.28		175.8	51.7	18.0	
Val[2]	7.78	4.21	2.04	0.91 , 0.90	173.0	59.7	32.5	19.7, 18.4
CF_3-Thr[3]	8.59	4.83	4.23	6.98	170.6	54.0	70.6	126.1
Val[4]	8.19	4.29	2.15	0.95, 0.92	173.5	59.8	31.8	19.7, 18.2
Leu[5 c]	8.20	4.41	1.59, 1.59	1.99	174.4	52.4	41.3	25.8,
				0.94, 0.89				23.3, 21.8

[a] The reported assignments correspond to the major Boc *anti* rotamer.

[b] Boc assignment: ^{1}H 1.43 ppm; ^{13}C 28.7, 80.7, 157.8 ppm. [c] OMe: ^{1}H 3.69 ppm; ^{13}C 52.6 ppm.

NMR study of pentapeptide **3b** in CD_3OH at 298 K

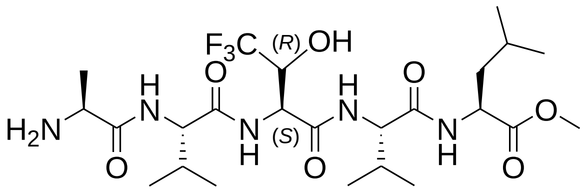

3b

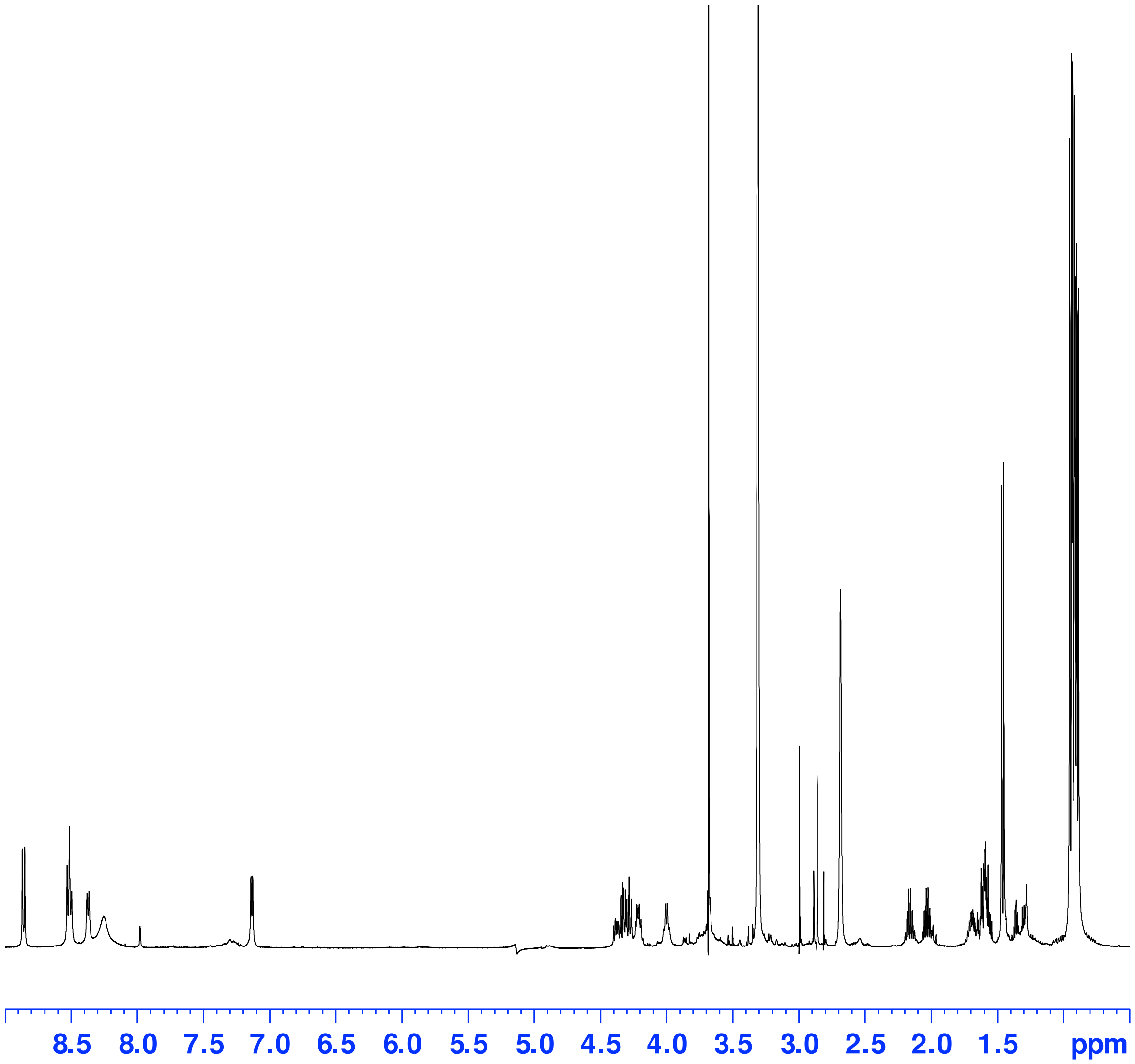

Figure S12 1D ^1H NMR spectrum of pentapeptide **3b** in CD_3OH (271 K)

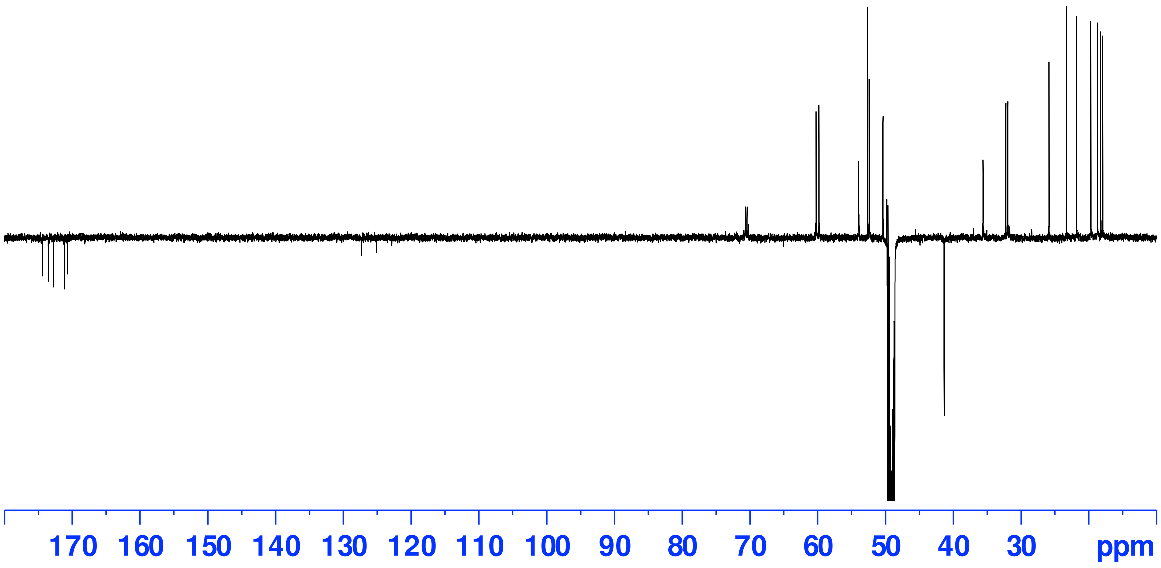

Figure S13 1D ^{13}C DEPTQ NMR spectrum of pentapeptide **3b** in CD_3OH (298 K)

Table S6 ^{1}H and ^{13}C NMR chemical shifts of pentapeptide **3b** in CD_3OH (298 K)

Residue	δ HN (ppm)	δ H^{α} (ppm)	δ H^{β} (ppm)	δ H^{γ} & H^{δ} (ppm)	δ CO (ppm)	δ C^{α} (ppm)	δ C^{β} (ppm)	δ C^{γ} & C^{δ} (ppm)
Ala^1	6.96	3.99	1.47		171.1	50.3	17.9	
Val^2	8.34	4.26	2.05	0.93	172.7	60.2	32.2	19.7, 18.7
CF_3-Thr^3	8.63	4.86	4.22		170.7	53.9	70.5	126.2
Val^4	8.24	4.30	2.15	0.95, 0.91	173.5	59.8	31.9	19.7, 18.2
Leu^5 [a]	8.22	4.41	1.60	1.69	174.4	52.4	41.3	25.8,
				0.94, 0.89				23.3, 21.8

[a] OMe assignment: ^{1}H 3.69 ppm; ^{13}C 52.6 ppm.

NMR study of pentapeptide (2*S*,3*S*)-**4a** in CD_3OH:

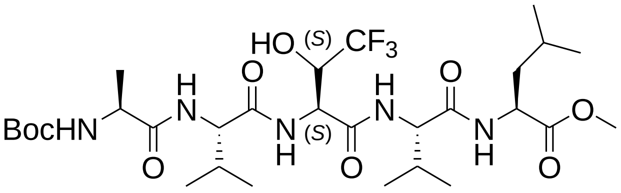

4a

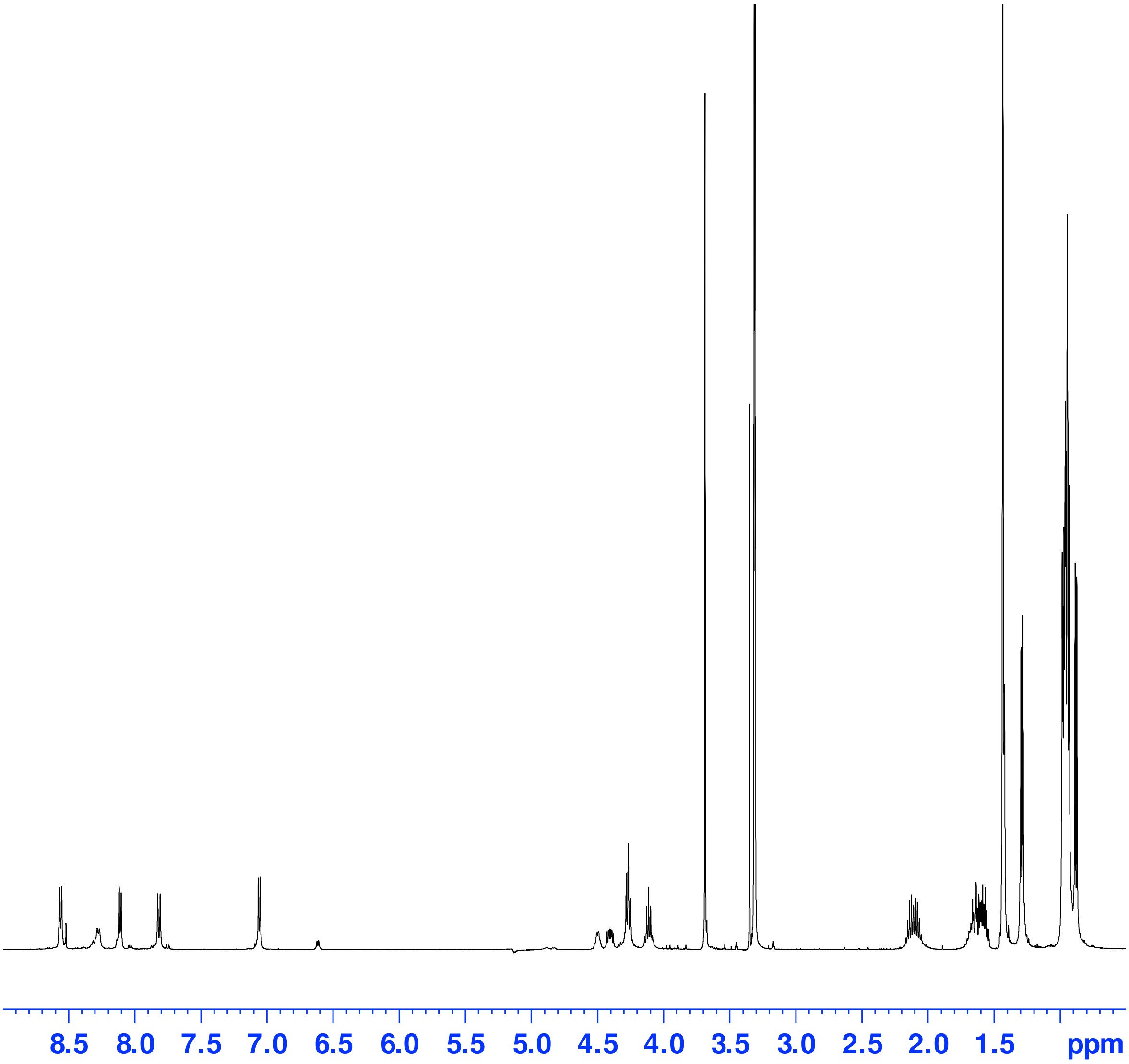

Figure S14 1D ^{1}H NMR spectrum of pentapeptide **4a** in CD_3OH (271 K)

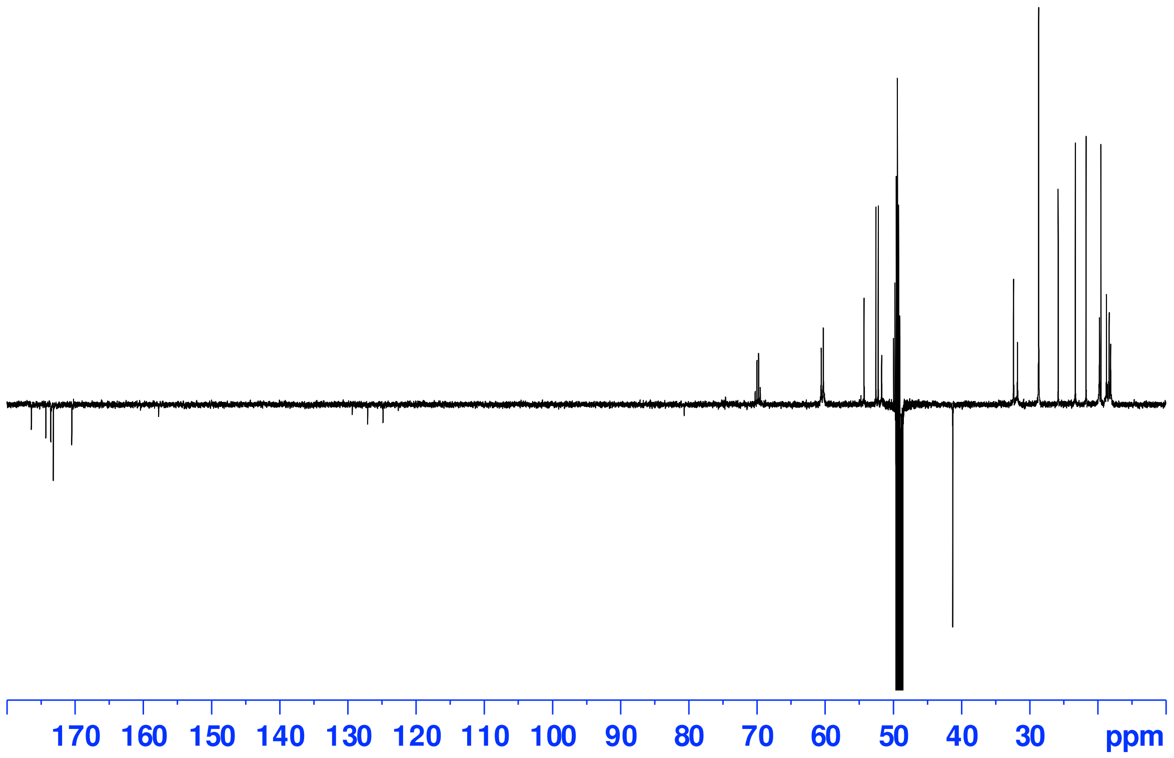

Figure S15 1D ^{13}C DEPTQ NMR spectrum of pentapeptide **4a** in CD_3OH (298 K)

Table S7 ^{1}H and ^{13}C NMR chemical shifts of pentapeptide **4a** in CD_3OH (298K) [a]

Residue	δ NH (ppm)	δ H^{α} (ppm)	δ H^{β} (ppm)	δ H^{γ} & H^{δ} (ppm)	δ CO (ppm)	δ C^{α} (ppm)	δ C^{β} (ppm)	δ C^{γ} & C^{δ} (ppm)
Ala[1 b]	6.86	4.13	1.30		176.4	51.7	18.1	
Val[2]	7.95	4.25	2.15	0.95, 0.98	173.6	60.6	31.8	18.3, 19.8
CF_3-Thr[3]	[c]	4.80	4.52		170.5	54.3	69.9	125.9
Val[4]	7.69	4.27	2.11	0.95, 0.97	173.2	60.3	32.4	18.8, 19.6
Leu[5 d]	8.31	4.43	1.61	1.67, 0.94, 0.88	174.3	52.2	41.3	25.8, 23.3, 21.7

[a] The reported assignments correspond to the major Boc *anti* rotamer.
[b] Boc assignment: ^{1}H 1.43 ppm; ^{13}C 28.7, 80.7, 157.7 ppm. [c] broad signal at 298 K. [d] OMe: ^{1}H 3.69 ppm; ^{13}C 52.6 ppm.

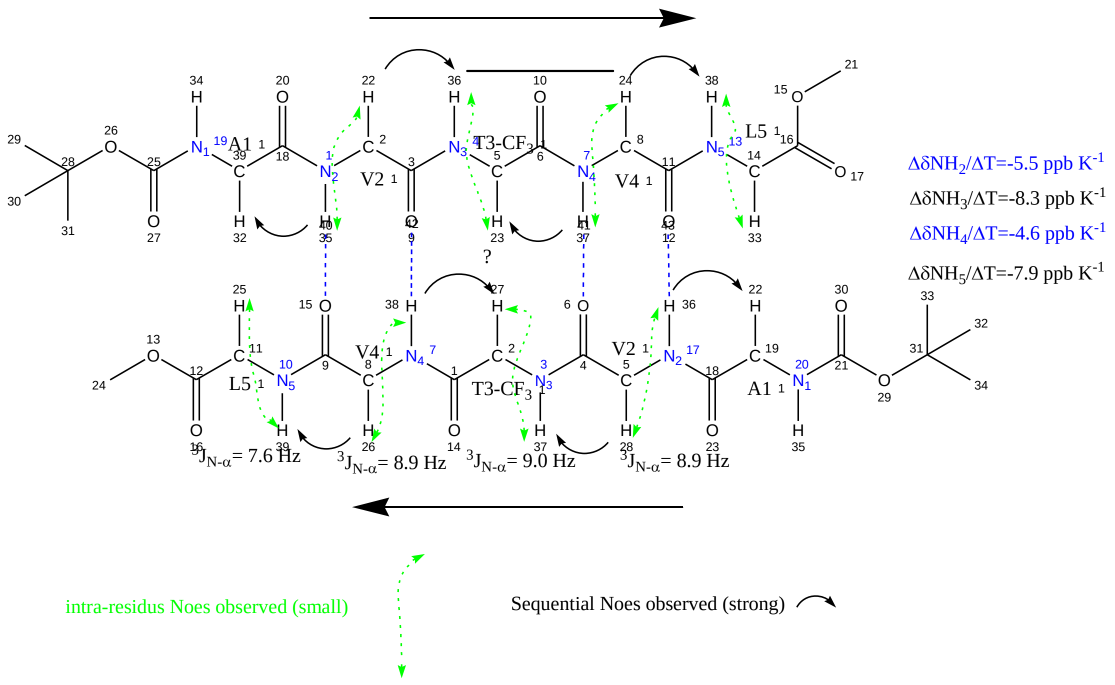

Figure S16 Hypothesis of anti-parallel β-sheet conformation in equilibrium with extended monomer of (2*S*,3*S*)-**4a**

NMR study of pentapeptide (2*S*,3*S*)-**4b**:

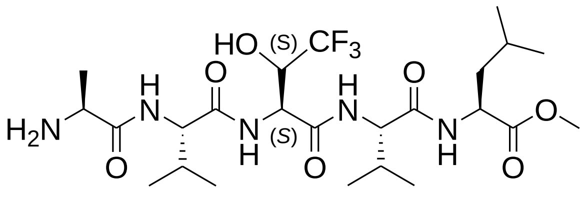

4b

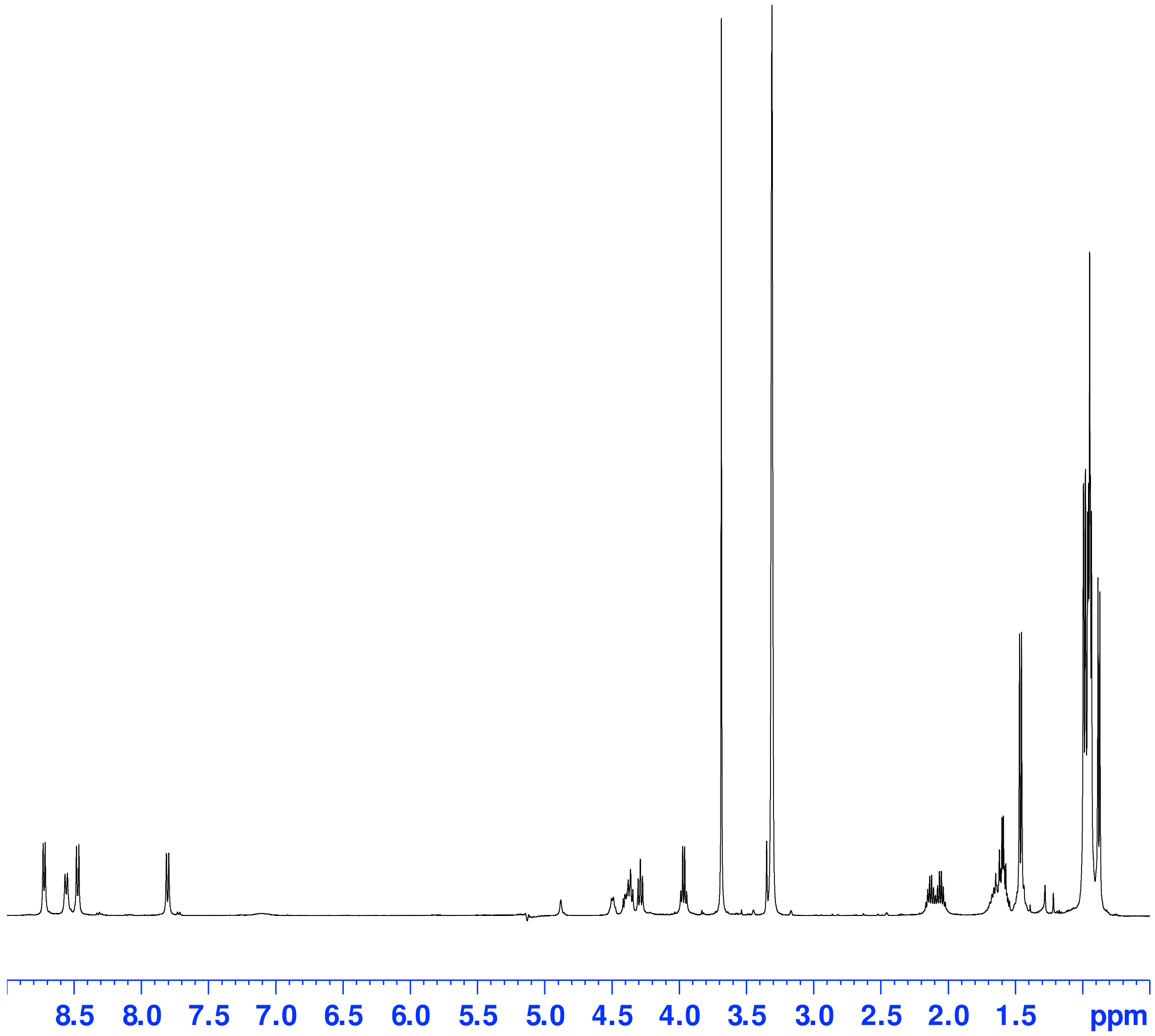

Figure S17 1D ^{1}H NMR spectrum of pentapeptide **4b** in CD_3OH (271 K)

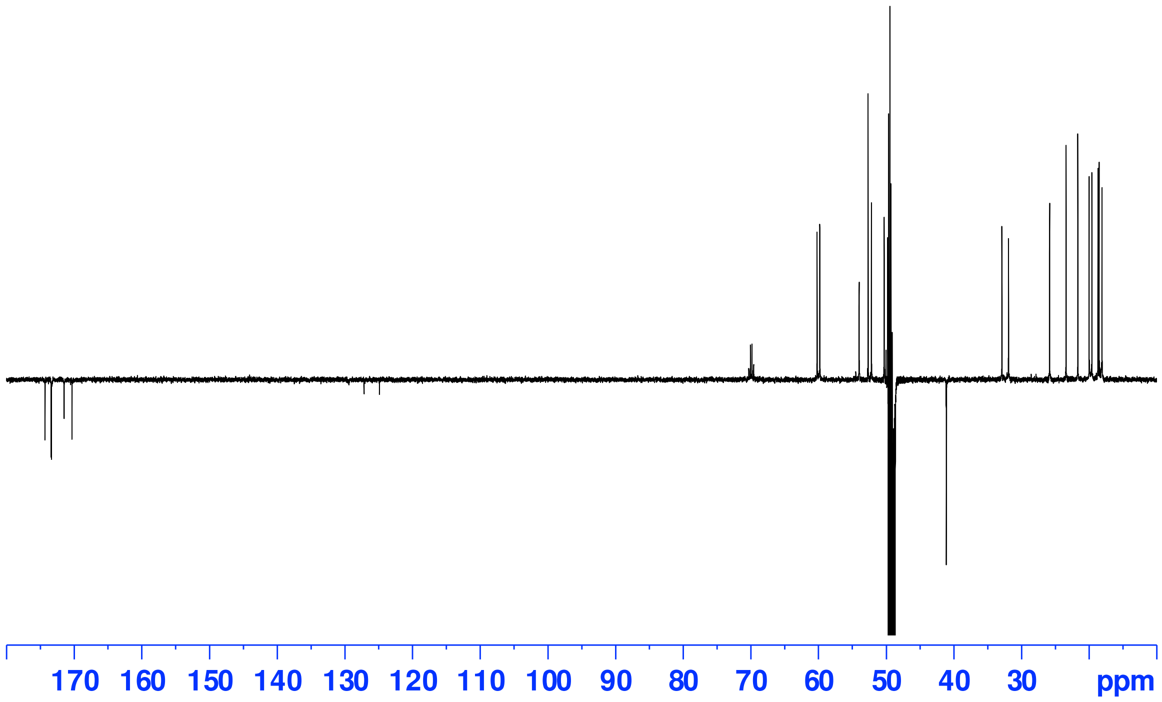

Figure S18 1D ^{13}C DEPTQ NMR spectrum of pentapeptide **4b** in CD_3OH (271 K)

Table S8 ^{1}H and ^{13}C NMR chemical shifts of pentapeptide **4b** in CD_3OH (298 K)

Residue	δ HN (ppm)	δ H^{α} (ppm)	δ H^{β} (ppm)	δ H^{γ} & H^{δ} (ppm)	δ CO (ppm)	δ C^{α} (ppm)	δ C^{β} (ppm)	δ C^{γ} & C^{δ} (ppm)
Ala^1	-	3.97	1.48		171.7	50.4	18.1	
Val^2	8.42	4.34	2.15	0.96, 0.99	173.3	60.2	31.7	18.5, 19.9
CF_3-Thr^3	8.24	4.84	4.52		170.3	54.1	70.0	126.0
Val^4	7.67	4.29	2.07	0.95, 0.98	173.2	59.8	32.8	18.6, 19.5
Leu^5 [a]	8.51	4.42	1.60	1.66, 0.93, 0.87	174.3	52.3	41.3	25.9, 23.3, 21.8

[a] OMe assignment: ^{1}H 3.69 ppm; ^{13}C 52.7 ppm.

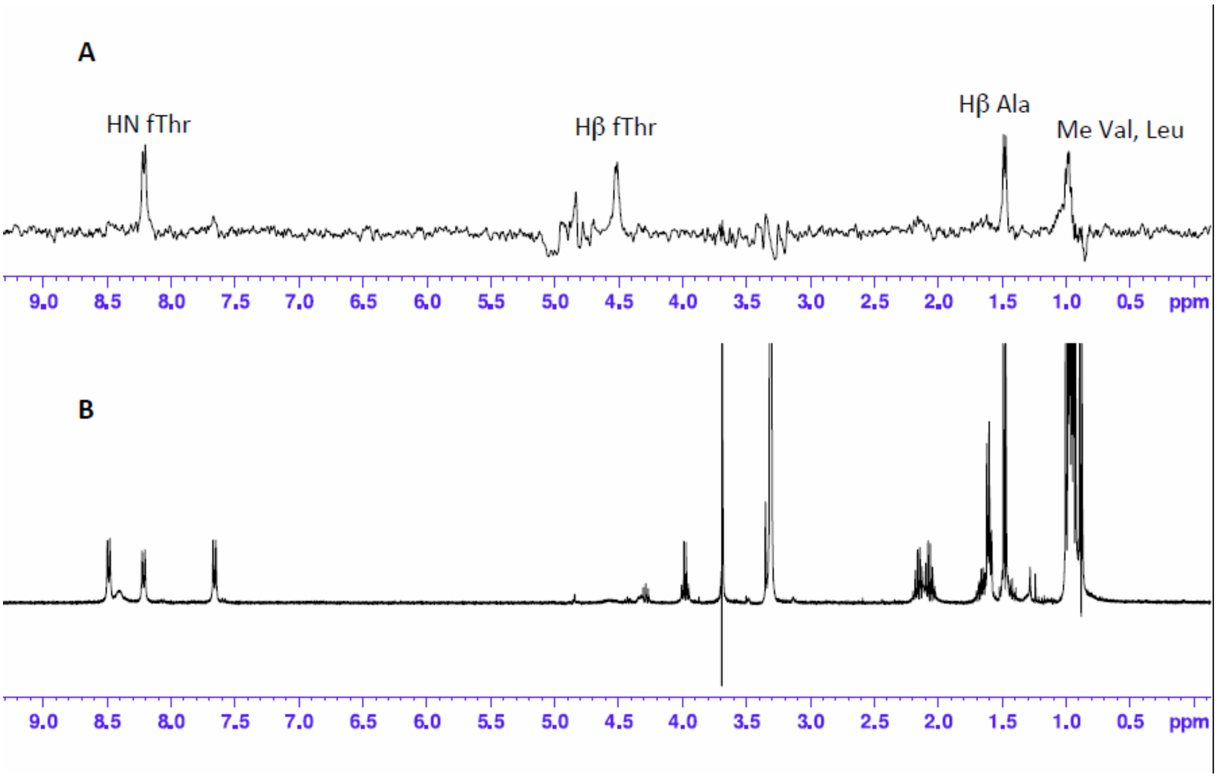

Figure S19 1D NOE $^{1}H\{^{19}F\}$ (A) and 1D ^{1}H Watergate (B) NMR spectra of pentapeptide **4b** in CD_3OH (298 K)

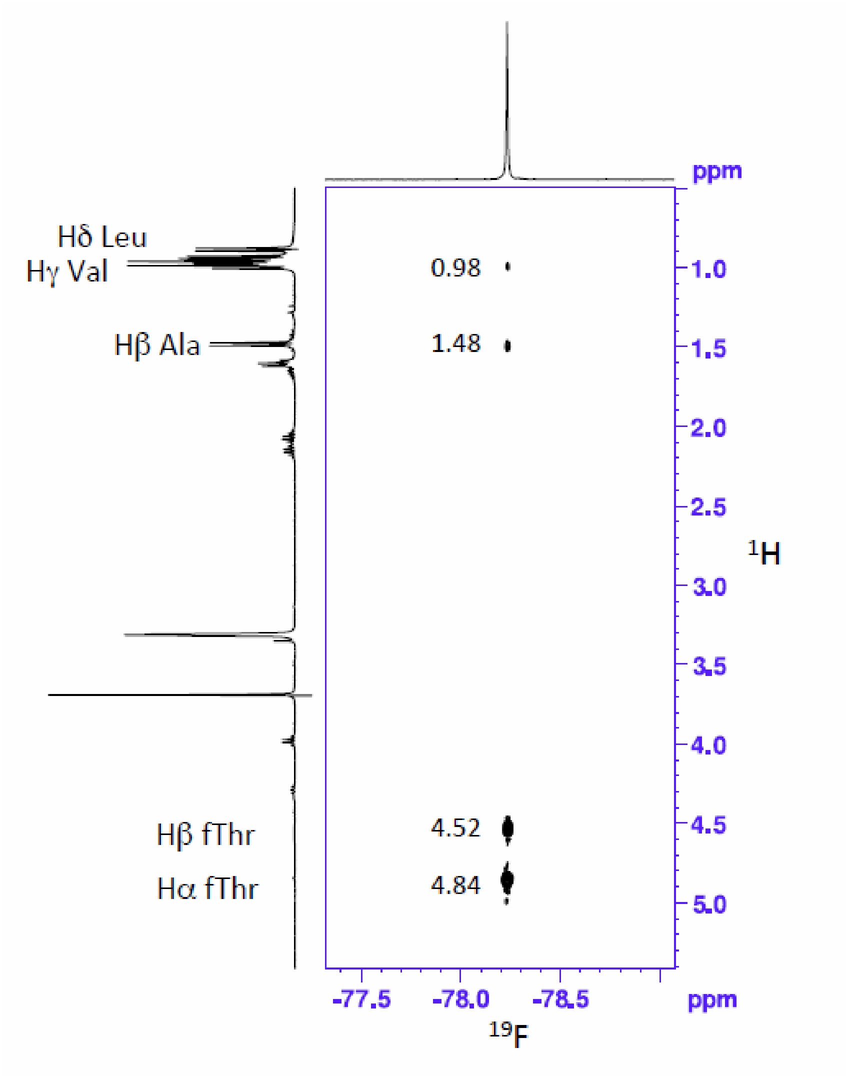

Figure S20 2D ^{1}H-^{19}F HOESY spectrum of pentapeptide **4b** in CD_3OH (298 K)

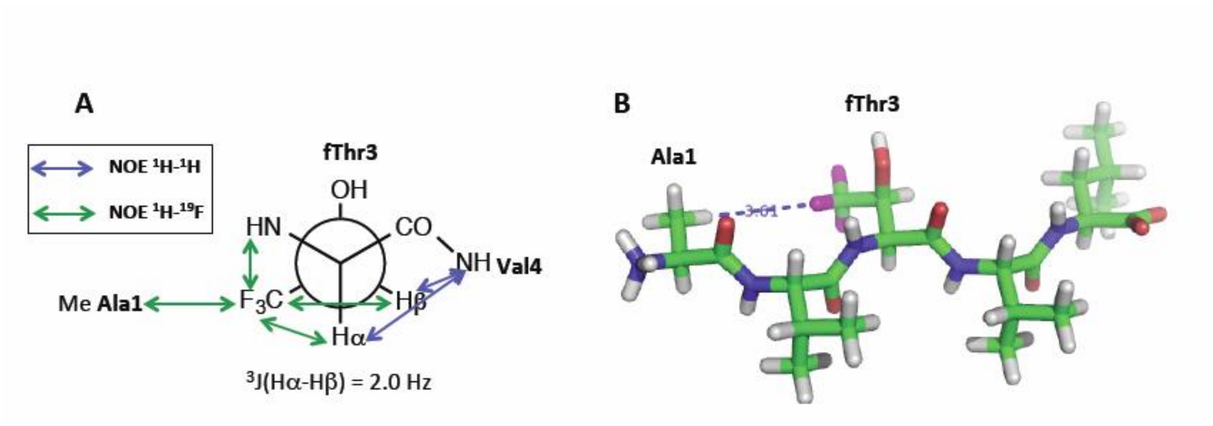

Figure S21 (A) Summary of NMR conformational parameters for the assignment of $\chi 1$ *gauche*+ rotamer of CF_3-Thr[3] in peptide **4b**. (B) Modelled β-conformation of peptide **4b** showing the close proximity of CH_3-Ala[1] and CF_3-Thr[3] groups.

Molecular dynamics studies: experimental procedure and data

All-atom MD simulations were performed using the GROMACS 4.5 package [10,11], with the OPLS-AA force field [12] for pentapeptides and MeOH, in combination with the SPC/E water model [13]. The extended conformation of each of the eight studied pentapeptides, in which all residues were in β-conformations (Figure S22), was initially placed in a 4.3 × 4.3 × 4.3 $Å^3$ box filled with about 2600 water molecules and one chloride ion to neutralize the systems in the case of the protonated free amine forms. The non-bonded interactions were treated using the smooth PME method [14] for the electrostatic terms and a cut-off distance of 1.2 nm for the van der Waals potentials. The covalent bonds length was kept constant using the LINCS [15] and SETTLE [16] procedures, allowing the use of a 2 fs time step. The simulated systems were first equilibrated using two short MD simulations of a 2 ns duration, the first one with the V-scale and Berendsen coupling algorithms [17] to rapidly reach the target temperature T = 300 K and pressure P = 1 bar respectively, and a second one with the Nose-Hoover and Parrinello-Rahman coupling methods [18-20] to generate correct temperature and pressure fluctuations around the previous equilibrium values. Finally, the systems were simulated without any constraints during 500 ns in the NPT ensemble. Trajectories coordinates were saved every 10 ps for subsequent analysis.

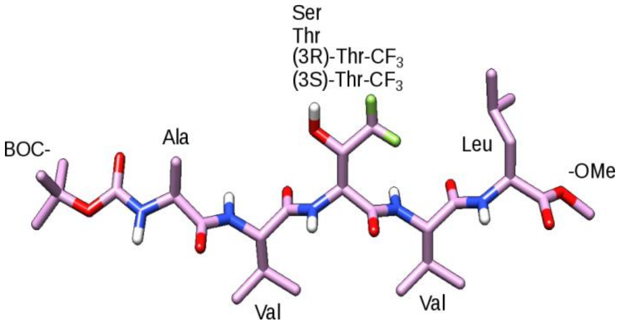

Figure S22: Initial conformations of the eight studied pentapeptides: Boc-AV**X**VL-OMe and NH_3-AV**X**VL-OMe, where X = Ser, Thr, (2*S*,3*R*)-CF_3-Thr and (2*S*,3*S*)-CF_3-Thr).

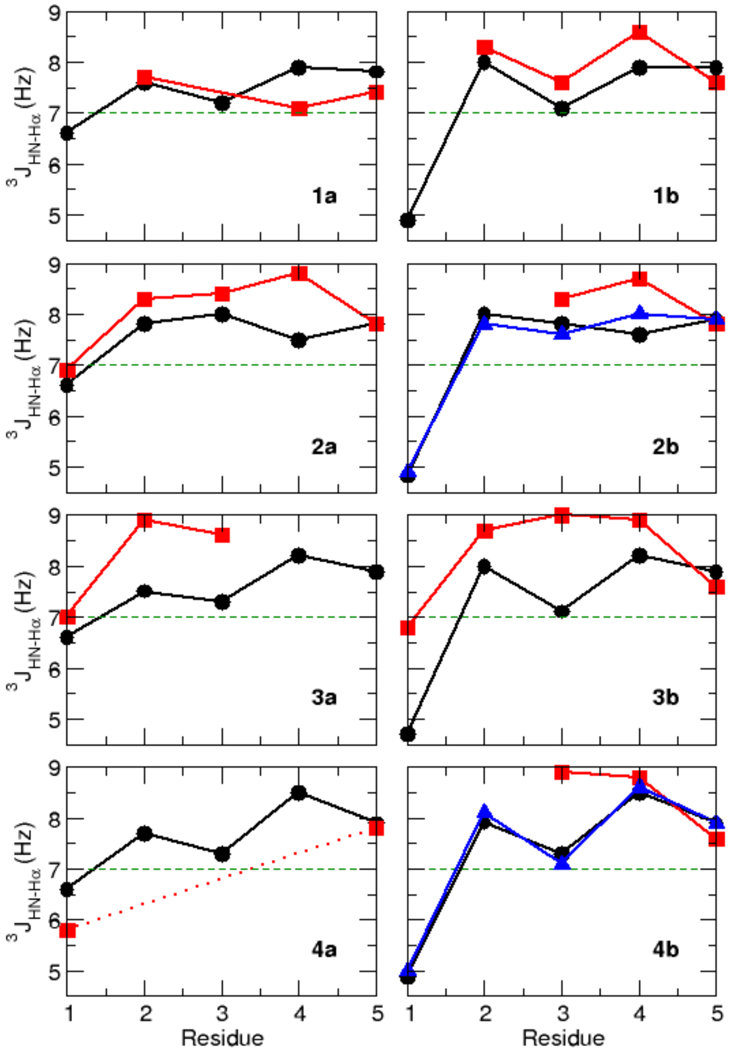

Figure S23 Comparison between the theoretical (black) and the NMR measured (red) $^3J_{HN\text{-}H\alpha}$ coupling constants as a function of the peptide residues. Theoretical $^3J_{HN\text{-}H\alpha}$ couplings in MeOH for **2b** and **4b** are shown in blue.

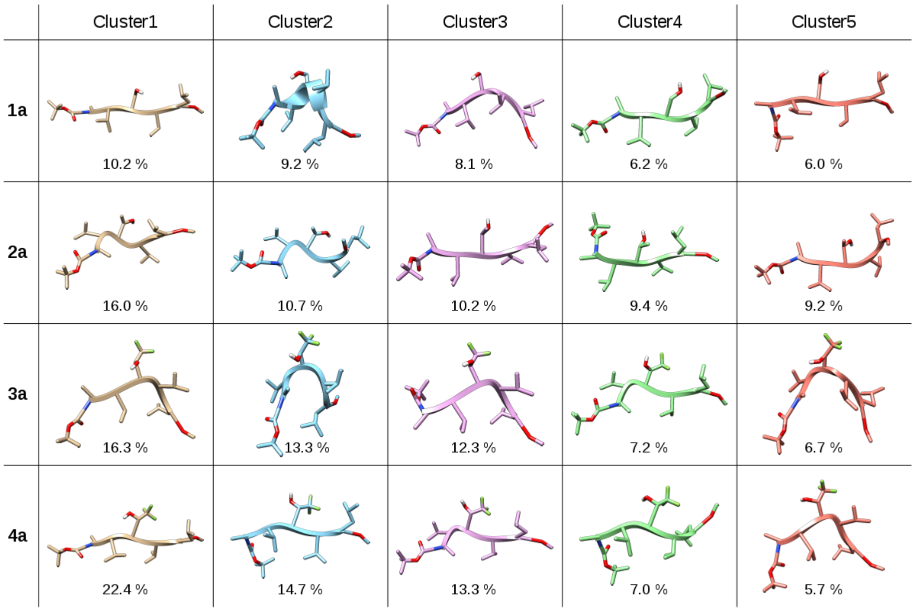

Figure S24 Representative structures of the five most populated clusters of the BOC-protected peptides.

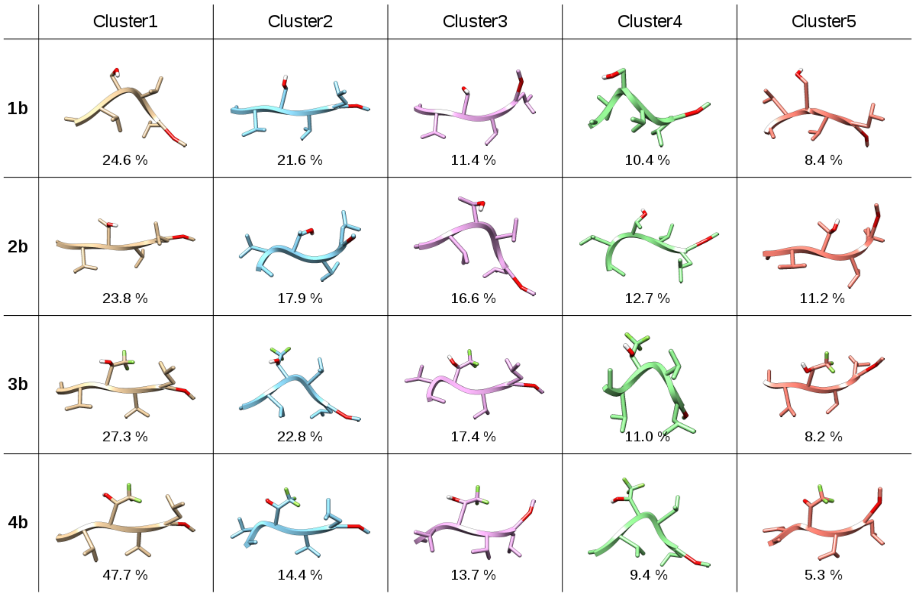

Figure S25 Representative structures of the five most populated clusters of the non-protected peptides.

Thioflavin-T fluorescence assays : Inhibition of A$\beta_{1\text{-}42}$ fibrillization

Experimental procedure for fluorescence-detected thioflavin-T binding assay (A$\beta_{1\text{-}42}$)

Thioflavin T was obtained from Sigma. A$\beta_{1\text{-}42}$ was purchased from American Peptide. The peptide was dissolved in an aqueous 1% ammonia solution to a concentration of 1 mM and then, just prior to use, was diluted to 0.2 mM with 10 mM Tris-HCl, 100 mM NaCl buffer (pH 7.4). Stock solutions of β-hairpin mimics were dissolved in DMSO with the final concentration kept constant at 0.5% (v/v).

Thioflavin-T fluorescence was measured to evaluate the development of $A\beta_{1\text{-}42}$ fibrils over time using a fluorescence plate reader (Fluostar Optima, BMG labtech) with standard 96-wells black microtiter plates. Experiments were started by adding the peptide (final $A\beta_{1\text{-}42}$ concentration equal to 10 µM) into a mixture containing 40 µM Thioflavin T in 10 mM Tris-HCl, 100 mM NaCl buffer (pH 7.4) with and without the tested compounds at two different concentrations (100 and 10 µM) at room temperature. The Th-T fluorescence intensity of each sample (performed in duplicate or triplicate) was recorded with 440/485 nm excitation/emission filters set for 42 hours performing a double orbital shaking of 10 s. before the first cycle. The fluorescence assays were performed between 2 and 4 times on different days, with two different batches of peptide. The ability of compounds to inhibit $A\beta_{1\text{-}42}$ aggregation was assessed considering the intensity of the experimental fluorescence plateau (F). The change of fluorescence intensity at the plateau is defined as the intensity of experimental fluorescence plateau observed with the tested compound relative to the value obtained without the compound and is evaluated as the following percentage : $(F_{A\beta+compound} - F_{A\beta})/ F_{A\beta} \times 100)$.

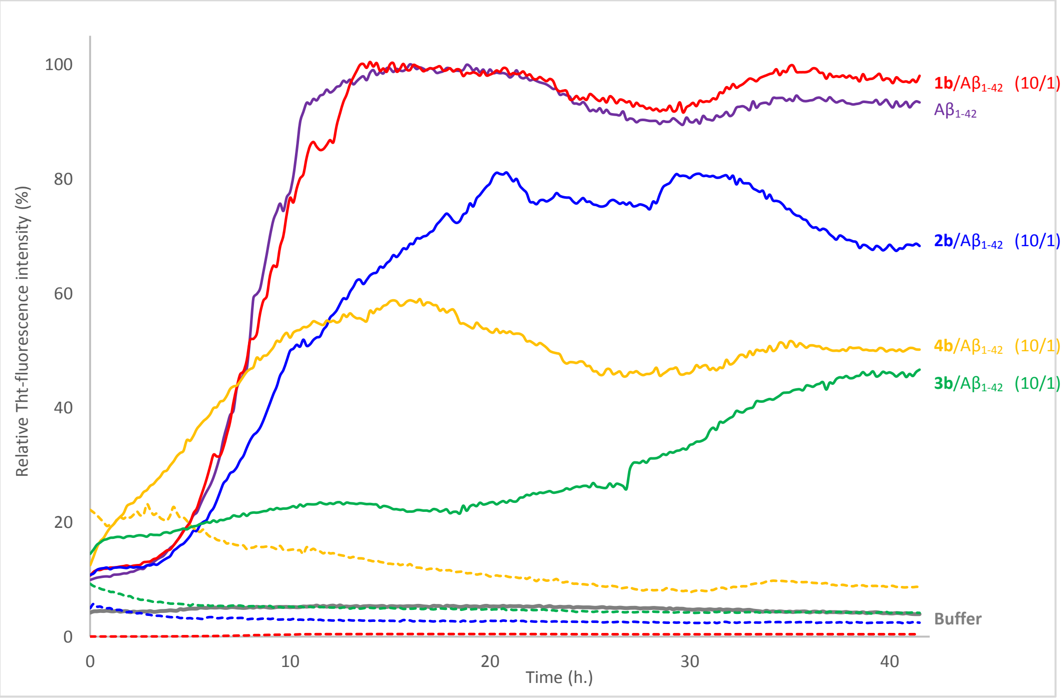

Figure S26. Representative curves of ThT fluorescence assays over time showing $A\beta_{1\text{-}42}$ (10 µM) aggregation in the absence (purple curve) and in the presence of compounds **1b** (red curve), **2b** (blue curve), **3b** (green curve) and **4b** (yellow curve) at compound/$A\beta_{1\text{-}42}$ ratios of 10/1. The control curves are represented in dotted lines (**1b** in red, **2b** in blue, **3b** in green, **4b** in yellow and buffer in grey).

Compound	Change of the fluorescence intensity at the plateau
**1b** 10:1	ne
**1b** 1:1	50 ± 23%
**2b** 10:1	−22 ± 5%
**2b** 1:1	35 ± 24%
**3b** 10:1	−60 ± 22%
**3b** 1:1	ne
**4b** 10:1	−56 ± 28%
**4b** 1:1	ne

Table S9. Effects of compounds **1b–4b** on $A\beta_{1-42}$ fibrillization assessed by the change of the fluorescence intensity at the plateau at 40 hours in the ThT-fluorescence assays, at 10:1 and 1:1 compound/Aβ ratios (the concentration of $A\beta_{1-42}$ is 10 μM) and compared to the values obtained for $A\beta_{1-42}$ alone.

The change of fluorescence intensity at the plateau is defined as the intensity of experimental fluorescence plateau observed with the tested compound relative to the value obtained without the compound and is evaluated as the following percentage : ($F_{A\beta+compound}$ − $F_{A\beta}$)/ $F_{A\beta}$ X 100).

ne = no effect, parameters are expressed as mean ± SE, *n* = 3.

References

1 Szilagyi, L.; Jardetzky, O. *J. Magn. Reson*. **1989**, *83*, 441–449.

2 Spera S.; Bax A. *J. Am. Chem. Soc.* **1991**, *113*, 5490–5492.

3 Wishart D. S.; Sykes, B. D. *Methods Enzymol.* **1994**, *239*, 363-392.

4 Wishart, D. S.; Sykes, B. D. *J. Biomol. NMR.* **1994**, *4*, 171–180.

5 Wishart, D. S.; Bigam, G. C.; Holm, A.; Hodges, R. S.; Sykes, B. D. *J. Biomol. NMR.* **1995**, *5*, 67-81.

6 Smith, L. J.; Fiebig, K. M.; Schwalbe, H.; Dobson, C.M. *Fold. Des*. **1996**, *1*, R95–R106.

7 Zeng, C. M.; Kerrigan, S. A.; Katzenellenbogen, J. A.; Slocum, C.; Gallacher, K.; Shomalic, M.; Lyttle, R.; Hattersley G.; Miller, C. P. *Tetrahedron Lett.* **2010**, *51*, 5361-5363.

8 Belokon, Y. N.; Tararov, V. I.; Maleev, V. I.; Savel'eva, T. F.; Ryzhov M. G. *Tetrahedron: Asymmetry* **1998**, *9*, 4249-4252.

9 Soloshonok, V. A.; Avilov, D. V.; Kukhar, V. P.; Tararov, V. I.; Savel'eva, T. F.; Churkina, T. D.; Ikonnikov, N. S.; Kochetkov, K. A.; Orlova, S. A.; Pysarevsky, A. P.; Struchkov, Y. T.; Raevsky N. I.; Belokon, Y. N. *Tetrahedron: Asymmetry* **1995**, *6*, 1741-1756.

10 Van Der Spoel, D.; Lindahl, E.; Hess, B.; Groenhof, G.; Mark, A.E.; Berendsen, H.J.C. *J. Comput. Chem.* **2015**, *26*, 1701-1718.

11 Hess, B.; Kutzner, C.; Van der Spoel, D.; Lindahl, E. *J. Chem. Theory Comput.* **2008**, *4*, 435-447.

12 Kaminski, G.A.; Friesner, R.A.; Tirado-Rives, J.; Jorgensen, W.L. *J. Phys. Chem. B* **2001**, *105*, 6474-6487.

13 Berendsen, H.J.C.; Grigera, J.R.; Straatsma, T.P. *J. Phys. Chem.* **1987**, *91*, 6269-6271.

14 Essmann, U.; Perera, L.; Berkowitz, M.L.; Darden, T.; Lee, H.; Pedersen, L.G. *J. Chem. Phys.* **1995**, *103*, 8577-8593.

15 Hess, B. *J. Chem. Theory Comput.* **2008**, *4*, 116-122.

16 Miyamoto, S.; Kollman, P.A. *J. Comput. Chem.* **1992**, *13*, 952-962.

17 Berendsen, H.J.C.; Postma, J.P.M.; Van Gunsteren, W.F.; DiNola, A.; Haak, J.R. *J. Chem. Phys.* **1984**, *81*, 3684-3690.

18 Nosé, S. *J. Chem. Phys.* **1984**, *81*, 511-519.

19 Hoover, W.G. *Phys. Rev. A* **1985**, *31*, 1695-1697.

20 Parrinello, M.; Rahman, A. *J. Appl. Phys.***1981**, *52*, 7182-7190.
